# Transmission routes of antibiotic resistant bacteria: a systematic review

**DOI:** 10.1186/s12879-022-07360-z

**Published:** 2022-05-20

**Authors:** Noortje G. Godijk, Martin C. J. Bootsma, Marc J. M. Bonten

**Affiliations:** 1 Julius Center for Health Sciences and Primary Care, University Medical Center Utrecht, Utrecht University, Utrecht, The Netherlands; 2 Department of Mathematics, Faculty of Sciences, Utrecht University, Utrecht, The Netherlands

**Keywords:** Antibiotic resistance, Antibiotic resistant bacteria, Transmission, Systematic review, *Escherichia coli*, *Staphylococcus aureus*, Enterobacteriaceae

## Abstract

**Background:**

Quantification of acquisition routes of antibiotic resistant bacteria (ARB) is pivotal for understanding transmission dynamics and designing cost-effective interventions. Different methods have been used to quantify the importance of transmission routes, such as relative risks, odds ratios (OR), genomic comparisons and basic reproduction numbers. We systematically reviewed reported estimates on acquisition routes’ contributions of ARB in humans, animals, water and the environment and assessed the methods used to quantify the importance of transmission routes.

**Methods:**

PubMed and EMBASE were searched, resulting in 6054 articles published up until January 1st, 2019. Full text screening was performed on 525 articles and 277 are included.

**Results:**

We extracted 718 estimates with *S. aureus* (*n* = 273), *E. coli* (*n* = 157) and Enterobacteriaceae (*n* = 99) being studied most frequently. Most estimates were derived from statistical methods (*n* = 560), mainly expressed as risks (*n* = 246) and ORs (*n* = 239), followed by genetic comparisons (*n* = 85), modelling (*n* = 62) and dosage of ARB ingested (*n* = 17). Transmission routes analysed most frequently were occupational exposure (*n* = 157), travelling (*n* = 110) and contacts with carriers (*n* = 83). Studies were mostly performed in the United States (*n* = 142), the Netherlands (*n* = 87) and Germany (*n* = 60). Comparison of methods was not possible as studies using different methods to estimate the same route were lacking. Due to study heterogeneity not all estimates by the same method could be pooled.

**Conclusion:**

Despite an abundance of published data the relative importance of transmission routes of ARB has not been accurately quantified. Links between exposure and acquisition are often present, but the frequency of exposure is missing, which disables estimation of transmission routes’ importance. To create effective policies reducing ARB, estimates of transmission should be weighed by the frequency of exposure occurrence.

**Supplementary Information:**

The online version contains supplementary material available at 10.1186/s12879-022-07360-z.

## Background

Infections caused by antibiotic-resistant bacteria (ARB) are a major global problem. Antibiotic resistance is as ubiquitous as bacteria are, and resistance genes can be found in humans, animals, water and the environment and can be transmitted between and within these reservoirs. The relative attribution of transmission routes differs between bacterial species and resistance elements [1].

While many studies have determined risk factors for acquisition, infection or colonisation with ARB, the global urgency to reduce the prevalence of antibiotic resistance calls for accurate quantification of transmission routes and their relative importance [2]. Chatterjee et al. [2] reviewed studies in which transmission routes were quantified based on odds ratios (OR), and concluded that uniform quantification of relationships between risk factors are required to better understand acquisitions of ARB in humans. Yet the contribution of transmission routes to the total number of acquisitions of ARB bacteria remains unknown. A better understanding of transmission routes and the One Health interplay of ARB is pivotal for developing targeted and cost-effective interventions to effectively reduce ARB transmission. 

Accurate quantification of the contribution of different transmission routes to the total burden of ARB firstly requires both the probability to become colonised when exposed as well as the frequency of exposure. For instance, a route with a low acquisition probability per exposure that occurs frequently, may contribute more to the total number of acquisitions than routes with infrequent exposure but with high transmission probability per exposure. The probability to become colonised has been estimated with different methods. Some studies use a risk estimate for colonisation with ARB when exposed and in others the genetic similarity of ARB was derived between source and receiver to establish transmission probability. Another approach is to use mathematical modelling to quantify the basic reproduction number, *R*_*0*_, defined as the expected number of secondary cases caused by a typical infected individual during its period of infectiousness in a completely susceptible population [3]. Such methodological differences may lead to different conclusions on the relative importance of transmission routes. We aimed to systematically review the literature for data on both the probability of acquisition when exposed and frequency of exposure, in order to quantify the importance of different transmission routes of ARB. Yet, despite an abundance of published data, there were no studies providing high-quality data on both parameters. Moreover, heterogeneity in study designs, methods and analytical approaches among studies investigating the same ARB was too high to perform pooled analyses in sensible manner. We, therefore, limit the reporting to a descriptive analysis of the studies included in the systematic review.

## Methods

### Search strategy

We performed a systematic review in accordance with the Preferred Reporting Items for Systematic Reviews and Meta-Analyses (PRISMA) protocol [4]. The protocol for this systematic review was registered on PROSPERO (CRD42019136298) and can be accessed at http://www.crd.york.ac.uk/PROSPERO/display_record.php?ID=CRD42019136298 [5]. 

We identified quantified estimates of transmission routes from humans, animals and environmental reservoirs that resulted in an acquisition of ARB. An acquisition is conceptualized as one or more of: exposure, intake, infection, acquisition, carriage and colonisation. Antibiotic resistance is defined as the resistance of bacteria to one or more antibiotics for which the bacterium is not intrinsically resistant.

**Fig. 1:**
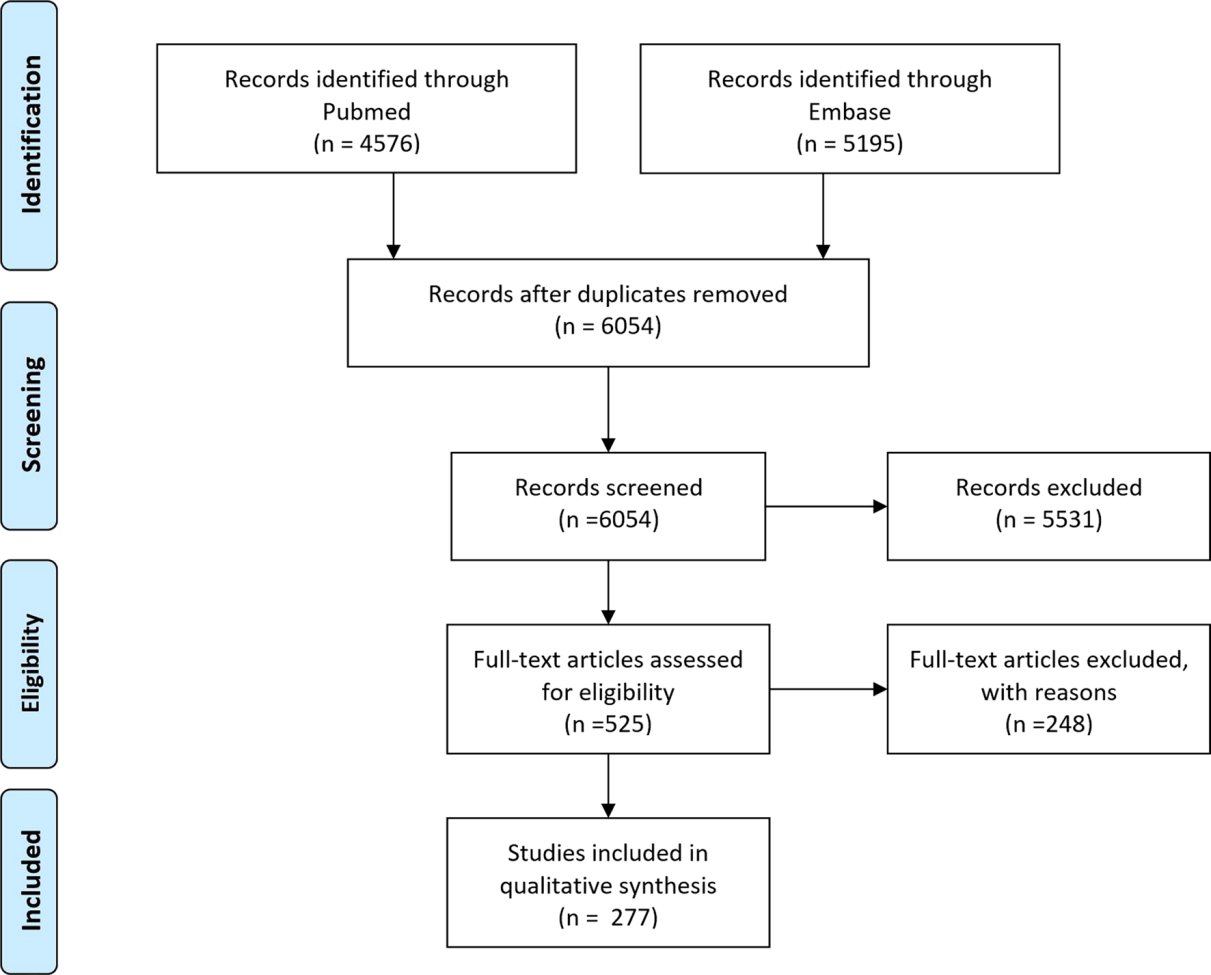
Prisma flow diagram of articles during the review

PubMed was searched using the search terms shown in Additional file 1: S1 Appendix, resulting in 4576 articles published until January 1st 2019. Secondly, Embase was searched using search terms shown in Additional file 1: S1 Appendix, which resulted in 5195 articles. Articles were not excluded based on publication date, publication type, sample size, significance level or quality Removing duplicates in Endnote and Rayyan resulted in 6054 articles for title/abstract screening. Two researchers separately in- or excluded a random subset of 50 of the 6054 articles, there was an agreement of 100%. Thereafter, one researcher performed the title/abstract screening in Rayyan [6]. In cases of doubt on in- or excluding an abstract/article, the second researcher was consulted. Five-hundred-and-twelve articles were included for full-text screening. After the full-text screening, 277 articles were included. Figure 1 shows a flow diagram of the articles in the review process. The PRISMA 2009 Checklist [4] can be found in Additional file 1: S2 Appendix.

### Study selection

Inclusion criteria for the final set of articles were (1) the presence of quantified estimates of (2) a clear acquisition route (3) of ARB. If the study included resistant and non-resistant pathogens the notation “rs” is placed after the transmission estimate to indicate so in the Additional file 1: Table S3. 

Studies were excluded if they only reported estimates of the percentage of the source colonised/infected with ARB, for example 10% of the cows on the farm were colonised with MRSA, as this is does not quantify how the cows acquired colonization. We excluded studies on horizontal plasmid transfer, within-host transfer of ARB, for instance from nasal to anal colonization within the same person, and antibiotics as a source of acquisition, because these routes of acquisition do not comprise transmission between different sources. Colonization pressure was excluded because we considered this transmission route too indirect. After applying these inclusion and exclusion criteria, 277 studies and 718 estimates remained.

### Data extraction

We extracted the method of estimation, transmission estimate, 95% CI, transmission route, pathogen, whether only resistant pathogens or a pooled estimated for resistant and susceptible pathogens was provided, country where study was performed, size of the study population, author, year of publication, title of manuscript, animal involved in the transmission route and in case of travelling as a transmission route, whether pre-travel screening had been performed. Study characteristics and outcomes were saved in a data extraction table using Microsoft Excel. If estimates based on multivariable analysis were available, these where extracted instead of estimates based on bivariate analysis.

### Quality assessment

Only the quality of travel studies was assessed by using a + or − to indicate whether pre-travel screening was performed or not.

### Data synthesis and analyses

*Reservoirs *Twelve reservoirs and cross-reservoirs in which transmission occurs were identified, (1) animal, (2) animal and food, (3) animal and human, (4) animal, human and water, (5) animal and environment, (6) animal and water, (7) human, (8) human and environment, (9) human and food, (10) human and water, (11) environment, and (12) environment and water. Travelling and studies researching an intervention to reduce transmission, e.g., handwashing, were not categorized as a reservoir. Although travelling frequently is considered a “route”, multiple transmission routes, such as food, people and environment, could cause acquisitions during travelling and therefore, we did not appoint travelling to a reservoir. 

*Transmission routes* In all 48 transmission routes were identified and frequencies are shown in the Additional file 1: Table S1. 

*Methods of estimation* Four methods for quantifying transmission routes were identified; [1] based on genetic similarity between ARB or resistance genes in source and receiver; [2] quantifying the dosage of ARB ingested by humans; [3] mathematical modelling to estimate transmission rates; and [4] statistical inference. The latter included calculations of OR, risks, prevalence ratio’s (PR), risk ratio’s (RR) and transmission rates (TR). Methods using genetic similarity estimated percentages of transmissions that had occurred (T, transmission percentage) or estimated a risk being the percentage in which acquisition with a genetically identical strain to the source had occurred from the total sample at risk (R, risk percentage). Modelling methods included *R0*, attributable percentage of the total number of cases to this route, transmission rates (TRs), cases per day, incidence, incidence rate ratios (IRRs) and acquisition rates (ARs). 

*Pathogens* To estimate transmission routes per ARB, each estimate was categorized per bacterial species if two or more estimates on this species were present, otherwise the estimate was categorized as “other”. The following 19 groups were identified *Staphylococcus aureus (S. aureus), Escherichia coli (E. coli),* Enterobacteriaceae (estimates pooled for multiple species of Enterobacteriaceae)*,* vancomycin resistant enterococci (VRE), staphylococci, *Pseudomonas aeruginosa* (*P. aeruginosa), Acinetobacter baumannii (A. baumannii), Acinetobacter calcoaceticus (A. calcoaceticus), Campylobacter, Enterococcus faecalis (E. faecalis), Enterococcus faecium (E. faecium), Streptococcus pneumoniae (S. pneumoniae),* group B streptococci, salmonella, *Staphylococcus epidermidis (S. epidermidis), Staphylococcus pseudintermedius (S. pseudintermedius), Staphylococcus haemolyticus (S. haemolyticus)* and other (*Enterococci, Mycoplasma hominis, Ureaplasma urealyticum, Klebsiella pneumoniae, Staphylococcus hominis, Staphylococcus cohnii, Klebsiella oxytoca, Haemophilus influenzae,* genes of a bacterial species (e.g., blaTEM). 

*Travelling* For travelling countries of destination were grouped in one of the following 11 groups; South Asia (Afghanistan, Bangladesh, Bhutan, Maldives, Nepal, India, Pakistan and Sri Lanka), South East Asia (Indonesia, Cambodia, Laos, Myanmar, Malaysia, Thailand, Vietnam, Australia, Brunei, the Philippines and Singapore), West Asia (Bahrain, Iran, Israel, Jordan, Kuwait, Lebanon, Oman, Qatar, Palestinian territories, Syria, Turkey, Iraq, Saudi Arabia, United Arab Emirates, and Yemen), Central & East Asia (China, Hong Kong, Mongolia, Uzbekistan, Turkmenistan, Tajikistan, Kyrgyzstan, Kazakhstan, East Turkestan, North Korea, South Korea, Japan, and Taiwan), Asia unspecified, Latin America (central Amerika, Mexico and south Amerika), North America, America unspecified, Africa (entire African continent), Europe and multiple regions (multiple of the above regions). 

*Meta-analysis* The initial plan was to perform random effects meta-analyses using the maximum-likelihood estimator per methodology per transmission route per pathogen for studies with the same outcome measure or outcome measures that could be lumped. Yet, as there were not enough studies providing high-quality data on the frequency of exposure and the probability of colonisation, we were unable to quantify the link between probability of colonisation and frequency of exposure. In addition, high heterogeneity in methods, study designs, and analysis performed within categories of ARB transmission routes investigated precluded performance of pooled analyses in sensible manner. We, therefore, limit the reporting to a descriptive analysis of studies included in the systematic review. Our script for the initial meta-analysis in R version 3.6.1. and the datasets generated during the current study are available in the GitHub repository, https://github.com/NoorGo/Transmission_routes_of_antibiotic_resistant_bacteria_systematic_review and as R files and Excel files in Additional file 2, Additional file 3 and Additional file 4.

## Results

### Study population

**Fig. 2:**
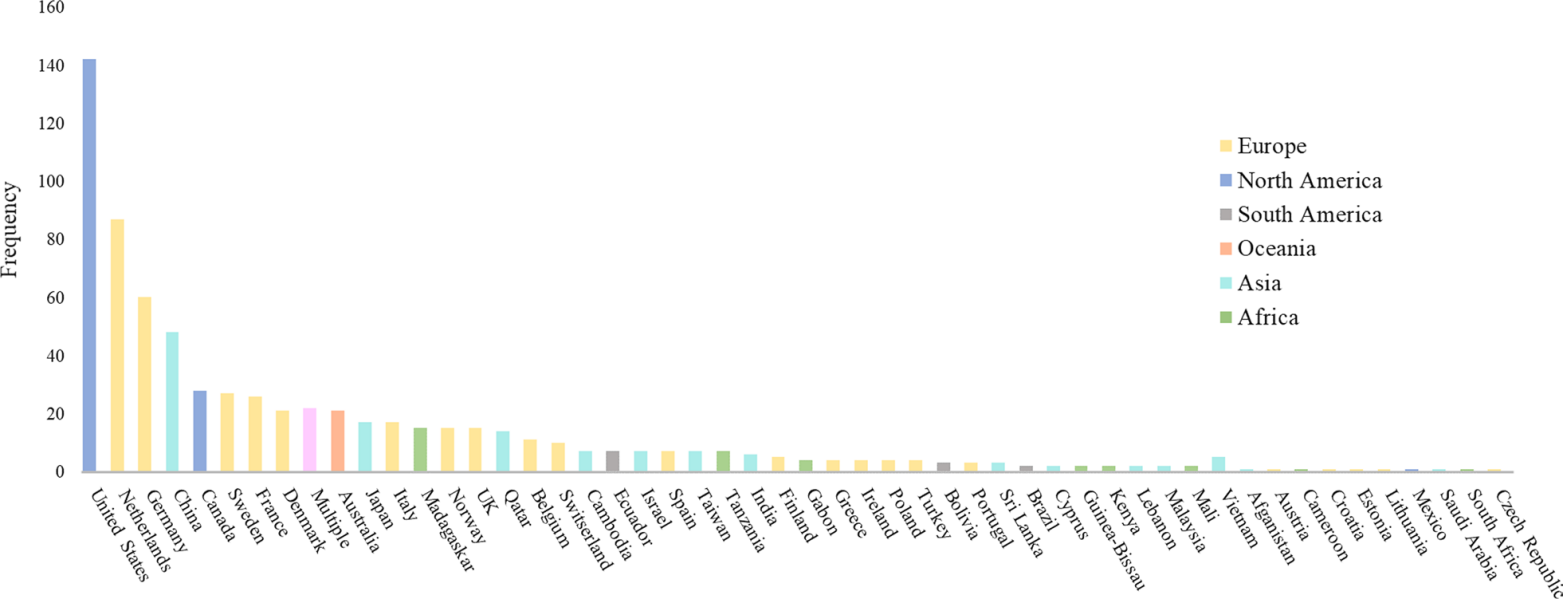
Countries per transmission route estimate *(n* = 718). Note: 5 studies were executed in laboratories and 8 were simulation studies and, therefore, not displayed in this figure

**Fig. 3:**
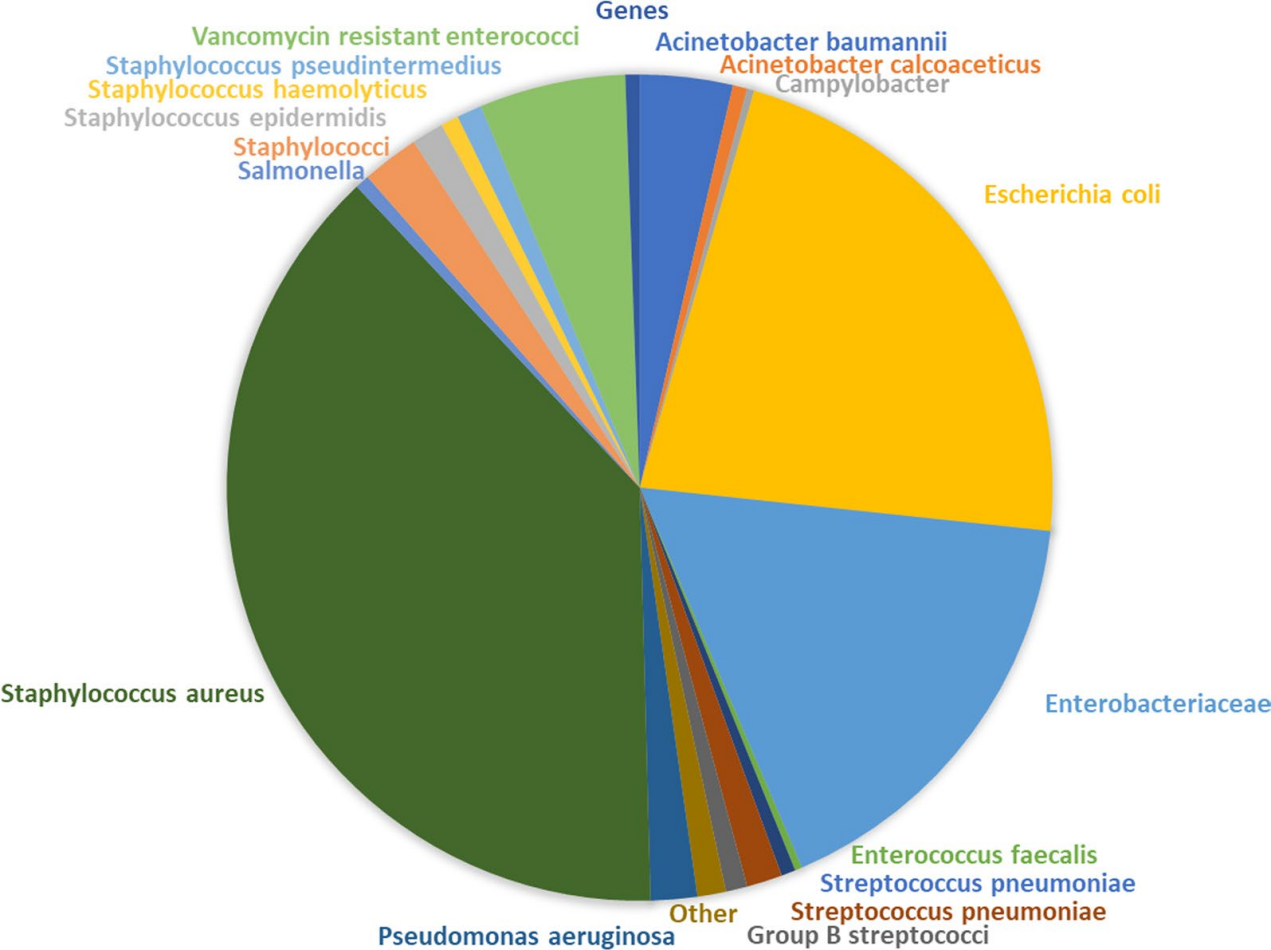
Chart of all bacteria studied

The 277 studies included yielded 718 transmission estimates of 40 transmission routes. Most studies had been performed in the United States (*n* = 142), the Netherlands (*n* = 87) and Germany (*n* = 60) (Fig. 2). Most estimates (*n* = 560, 78%) were based on statistical inference, predominantly yielding risks (*n* = 246, 44%) or ORs (*n* = 239, 43%) (Table 1). Seventeen studies (2%) quantified the dosage of ARB ingested by humans. The most commonly studied transmission routes were occupational exposure (*n* = 157, 22%), travelling (*n* = 110, 15%) and contact with carriers (*n* = 83, 12%) as can be seen in the Additional file 1: Table S1. Most estimates based on travelling (*n* = 70, 64%) included pre- and post-travel screening. The bacteria most commonly studied were *S. aureus* (*n* = 275, 38%), *E. coli* (*n* = 160, 22%) and Enterobacteriaceae (*n* = 121, 14%) as can be seen in Fig. 3 and Additional file 1: Table S2. A list of included articles is reported in the Additional file 1: Table S3. Additional file 1: Table S3 also states which method of estimation was performed, which bacteria and which transmission route was studied for each article.

**Table 1:** Frequencies of methods of estimation

Method of estimation (*n)*		Specification (*n*)	
Statistics	560	Odds ratio	239
		Risk	246
		Prevalence ratio	63
		Transmission rate	5
		Risk ratio	3
		Risk difference	4
Genes	85	Identical overlap	85
Modelling	56	R0	21
		Attributable % (Importance of route)	10
		Transmission rate	8
		Cases per day	6
		Incidence	4
		Incidence rate ratio	6
		Acquisition rate	1
Bacteria intake	17		
Total	718

### Cross-reservoir

**Fig. 4:**
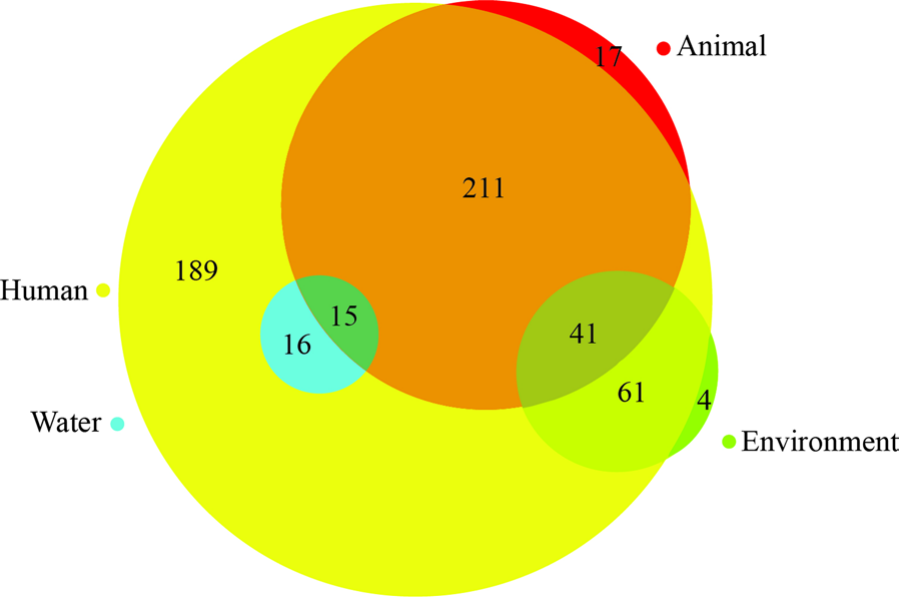
Reservoirs and cross-reservoir transmission in the study

Cross-reservoir transmission between animals and humans was studied most frequently (*n* = 211, 29%). Other routes studied were transmission between environments, such as soil, rooms or utensils, and humans (*n* = 61, 9%), between water and humans (*n* = 16, 2%), between water and animals (*n* = 15*,* 2%), and between animals and the environment (*n* = 41, 6%) (Fig. 4).

### The influence of methodologies on the comparability transmission route importance

Determination of the importance of transmission routes estimated with the above mentioned methodologies was difficult, because not a single transmission route was estimate by all methodologies. This review found that, when applied to the same dataset, risk-based estimates which did not use available genetic data, such as whole-genome sequencing (WGS), tend to overestimate transmission risk compared to estimates including this genetic data as indicated in multiple studies [7–14]. This should be the case, as the genetic data narrow the definition of a potential transmission event, not only should the donor and the recipient have an epidemiological link, both should have the same or a genetically closely related variant of the strain as well. 

### Importance of transmission routes

Chatterjee et al. [2] provided an overview of ARB transmission to humans estimated by OR, and suggest that uniformly quantifying relationships between risk factors will help to understand the process of ARB acquisition in humans. Here we take all methods to estimate ARB transmission into account, but this still does not allow a proper quantification of the importance of different ARB acquisition routes. We detect a missing link between the risk of acquisition when exposed and frequency of exposure. We aimed to include studies quantifying sources of infections in humans, but only found 1 study which attempted to do so. Cuny et al. [15] take a first step in creating a bigger picture of transmission sources by estimating that, of a large sample of MRSA infections in humans in Germany from 2006 to 2014, 0.14% originated from horses.

We will briefly discuss the transmission routes with the highest acquisition risk given exposure found with the different methods for the largest bacteria groups; *S. aureus, E. coli* and Enterobacteriaceae.

*S. aureus* Exposure to pigs and close contact with infected people were frequently identified by multiple methods as high risk transmission routes. For OR, transmission in the human reservoir yielded multiple high estimates, specifically within close contact such as in families. Although OR are high, the absolute risk was estimated low. Based on genetic comparisons contact with a household member yielded high risks for transmission of *S. aureus*. In other studies, genetical overlap was used to determine the number of acquisitions that could be correctly attributed to the source. Co-occurrence in humans showed the highest proportion of genetically identical strains. When comparing statistically calculated risk estimates to genetical analysis we found that risk estimates of human-to-human transmission based on genetic overlap were the closest to mere statistical risk estimates compared to other transmission routes. For other routes the difference between the estimated risks by these two methods was larger. For example, those thought to be infected by a family member had in 55% [26–80%] an identical strain, which means that in 45% it could not be demonstrated that an infected family member was the source. In this case, a simple risk estimate would have been an overestimation as genetical analysis shows that not colonisations could be attributed to the colonised household member. 

Moreover, transmission between the human and animal reservoir, specifically pigs resulted in high OR, usually with large confidence intervals, the highest PR, high risks and the highest risk estimates based on genetic overlap. *R*_*0*_ estimates also indicated pigs as an imported transmitter as pig to pig transmission of *S. aureus* studied in the Netherlands also had the highest pooled *R*_*0*_ (4.04, 95% CI [2.78–5.86]) [16].

*E. coli *The human reservoir is a likely route of high *E. coli* transmission. Close contact with infected people was identified by OR and risk estimates as a high risk *E. coli* transmission route. Risk estimates based on genetic overlap showed that almost halve of the household members at risk had an *E. coli* acquisition genetically identical to their household member. OR and risks also indicate animal to environment and animal to human transmission, especially poultry, to be a higher risk route. Travelling to Asia was also identified by OR as high risk. 

*Enterobacteriaceae* Close contact was indicated by OR and PR to be high risk routes. Travelling to Asia was indicated to be high risk by OR and risk estimates. Figures on risk and OR when travelling to regions across the world can be found on GitHub (https://github.com/NoorGo/Transmission_routes_of_antibiotic_resistant_bacteria_systematic_review/tree/Travelling-Figures-ORs-and-Risks).

## Discussion

Despite a large number of studies which investigated the probability of acquisition when exposed, we did not identify data that link the probability of acquisition to a frequency of exposure. Information to provide an accurate qualification of the importance of transmission routes was not found.

Nevertheless, some examples allowing quantification do exist. Cuny et al. [15] estimated 0.14% of MRSA infections in humans in Germany between 2006 and 2014 originated from horses. Furthermore, several studies yielded high genetic overlap, OR and PR for bacterial transmission between mother and child during breastfeeding. Yet, as this only occurs in the first years of life this route will not contribute to the total number of transmissions. Regarding the missing link between frequency of exposure and probability of acquisition, multiple studies reported that increased frequency of exposure to animals increased risk of acquisition of MRSA for humans. Increasing hours per day [17, 18], days per week [18, 19], number of animals [19, 20] and number of years of occupational exposure [18, 20] are indicated to increase colonization of farmers which supports our view that a bridge between frequency of exposure and risk of transmission is important.

This systematic review shows that statistical methods, especially ORs and risks, are most often used to estimate transmission routes. Cross-reservoir transmission between humans and animals has been studied most commonly, whereas studies on transmission from the environment are scarce, as also indicated by a previous meta-analysis which only included significant transmission estimates [2]. 

Comparison of transmission route estimates calculated using different methodologies was difficult as we found few transmission routes that were estimated using multiple methods in comparable populations. Also, no study was identified which conducted all methodologies on the same dataset to study a transmission route. Methods of estimation seems to influence the interpretation of what sources are more likely to cause acquisition. This review found multiple studies indicating that risk-based analyses which do not use genetic data, such as WGS, tend to overestimate the risk compared to studies using genetic data [7–14]. Davis et al. [21] emphasize the added value of whole-genome sequencing, as their previous indicated transmission routes appeared not to have occurred when they compared strains of animals and humans.

Strengths of this review include the assessment and comparison of various methods of estimation, reservoirs and transmission routes so that a complete overview of the current literature on ARB is provided. The first limitation of our study is data collection was done by one reviewer which might have caused discrepancies in data collection and analysis, however measures were taken to limit discrepancies such as a subset which was screened by two authors. Secondly, the extensive literature on the transmission of ARB precludes our search terms from identifying all studies in this area. By including synonyms, we tried to identify as many of the studies as possible. Moreover, publication bias could be present in different degrees for different methods of estimation. For example, whole genome studies that do not identify possible transmission chains may be published less frequently. However, publication bias also occurs for statistical methods of estimation such as OR that do not identify significant routes. Contrary to other reviews [2] we did include non-significant findings, however we cannot control publication bias differences between different methods of estimation.

## Conclusion

To conclude, on the one hand, there is a plethora of estimates on the probability of acquisition for ARB transmission routes. On the other hand, the relevance of routes on a population scale is missing due to a missing link between probability of acquisition and frequency of exposure. This lack of knowledge prohibits an accurate prediction of the effectiveness of interventions which target one or more transmission routes. To better quantify acquisition risks in different transmission routes we recommend researchers to perform more focussed and more detailed studies. For instance, population-based studies in well described niches, such as hospitals, other health care facilities, or farms, in which samples can be collected longitudinally in all subjects and environments that may be involved in transmission, as has been performed by Pham et al. [22]. By sampling longitudinally from the environment and subjects one can asses colonization status and the frequency of exposure. Longitudinal studies researching the frequency of exposure, for example how often someone eats raw meat or has contact with animals, and colonization status can provide inside in the link between frequency of exposure and acquisition of ARB. However, measuring the colonization status of the source exposed to, for example is the meat indeed colonised as well, may give problems in practice and therefore, the study design has to be carefully thought out.Large-scale population studies may also reveal reliable information, see for instance Cuny et al. [15] and Mughini-Gras et al. [23]. Yet, such studies will always suffer from missing information as not all subjects and environments can be sampled, requiring statistical adjustments.

## Supplementary Information

**Additional file 1.** Supporting information including the search terms, Prisma checklist, frequency tables and a list of which studies were included in the review.

**Additional file 2.** Transmission data identified in the systematic review by Godijk et al.

**Additional file 3.** R Script of the systematic review by Godijk et al.

**Additional file 4.** R script of the forest plots for the systematic review of Godijk et al.

## Data Availability

The datasets and scripts generated during the current study are available in the GitHub repository, https://github.com/NoorGo/Transmission_routes_of_antibiotic_resistant_bacteria_systematic_review and as R files and Excel files in Additional file 2, Additional file 3 and Additional file 4.

## Abbreviations

*A. baumannii*: *Acinetobacter baumannii*
*A. calcoaceticus*: *Acinetobacter calcoaceticus*
ARB: Antibiotic resistant bacteria
*E. coli*: *Escherichia coli*
*E. faecalis*: *Enterococcus faecalis*
*E. faecium*: *Enterococcus faecium*
MRSA: Methicillin resistant *Staphylococcus aureus*
PRISMA: Preferred Reporting Items for Systematic Reviews and Meta-Analyses
*P. aeruginosa*: *Pseudomonas aeruginosa*
VRE: Vancomycin resistant enterococci
*S. aureus*: *Staphylococcus aureus*
*S. epidermidis*: *Staphylococcus epidermidis*
*S. haemolyticus*: *Staphylococcus haemolyticus*
*S. pneumoniae*: *Streptococcus pneumoniae*
*S. pseudintermedius*: *Staphylococcus pseudintermedius*
OR: Odds ratios
WGS: Whole-genome sequencing
