# Supplementary material for: Transmission routes of antibiotic resistant bacteria: a systematic review
Source: BMC Infect Dis. 2022 May 20;22:482. doi: 10.1186/s12879-022-07360-z (PMC9123679; doi:10.1186/s12879-022-07360-z)
Supplement: Supplementary file 1 — Additional file 1. Supporting information including the search terms, Prisma checklist, frequency tables and a list of which studies were included in the review. [file 12879_2022_7360_MOESM1_ESM.docx]

**Supporting information for**

**Transmission routes of antibiotic resistant bacteria: a systematic review**

**Noortje G. Godijk*^1^, Martin C.J. Bootsma^1,2^, Marc J.M. Bonten^1^**^1^Julius Center for Health Sciences and Primary Care, University Medical Center Utrecht, Utrecht University, NL
^2^Department of Mathematics, Faculty of Sciences, Utrecht University, Utrecht, NL

^*^Corresponding author: Noortje G. Godijk ([n.g.godijk-2@umcutrecht.nl](mailto:n.g.godijk-2@umcutrecht.nl))

**This file includes:**

S1 Appendix. Search terms.

S2 Appendix. Prisma Checklist.
S1 Table. Frequencies of each transmission route identified.
S2 Table. Frequencies of bacteria studied for transmission routes included in quantitative synthesis.
S3 Table. List of studies of which estimates were included in the synthesis.
Description additional files.

**S1 Appendix. Search terms.**
Search in PubMed, 01-01-2019
((((((((((((((resistant[Title/Abstract]) OR resistance[Title/Abstract])) AND ((((((bacterial[Title/Abstract]) OR antibiotic[Title/Abstract]) OR drug[Title/Abstract]) OR antibacterial[Title/Abstract]) OR antimicrobial[Title/Abstract]) OR microbial[Title/Abstract]))) OR antibiotic resistance, bacterial[MeSH Terms]))) AND ((((((((acquiring[Title/Abstract]) OR acquisition[Title/Abstract]) OR transmission[Title/Abstract]) OR spread[Title/Abstract]) OR dissemination[Title/Abstract]) OR exposure[Title/Abstract]) OR exposed[Title/Abstract]) OR intake[Title/Abstract]))) AND (((((((colonization[Title/Abstract]) OR colonized[Title/Abstract]) OR colonisation[Title/Abstract]) OR colonised[Title/Abstract]) OR carriage[Title/Abstract]) OR carrying[Title/Abstract])))) NOT ((((((((((((fung*[Title/Abstract]) OR viral[Title/Abstract]) OR virus[Title/Abstract]) OR implant*[Title/Abstract]) OR HIV[Title/Abstract]) OR molecular[Title]) OR cancer[Title/Abstract]) OR tumor[Title/Abstract]) OR tumour[Title/Abstract]) OR neoplasm[Title/Abstract]) OR neoplasms[Title/Abstract])))) AND (((english[Language]) OR dutch[Language]))
Results:4576
Search in Embase, 01-01-2019
(('bacterial':ab,ti OR 'antibiotic':ab,ti OR 'drug':ab,ti OR 'antibacterial':ab,ti OR 'antimicrobial':ab,ti OR 'microbial':ab,ti) AND ('resistant':ab,ti OR 'resistance':ab,ti) OR 'antibiotic resistance'/exp) AND ('acquiring':ab,ti OR 'acquisition':ab,ti OR 'transmission':ab,ti OR 'spread':ab,ti OR 'dissemination':ab,ti OR 'exposure':ab,ti OR 'exposed':ab,ti OR 'intake':ab,ti) AND ('colonization':ab,ti OR 'colonized':ab,ti OR 'colonisation':ab,ti OR 'colonised':ab,ti OR 'carriage':ab,ti OR 'carrying':ab,ti) NOT ('fung*':ab,ti OR 'viral':ab,ti OR 'virus':ab,ti OR 'implant*':ab,ti OR 'hiv':ab,ti OR 'molecular':ti OR 'cancer':ab,ti OR 'tumor':ab,ti OR 'tumour':ab,ti OR 'neoplasm':ab,ti OR 'neoplasms':ab,ti) AND ([dutch]/lim OR [english]/lim)
Results: 5195
After removing duplicates of the separate searches: 6054 results

**S2 Appendix. Prisma Checklist**


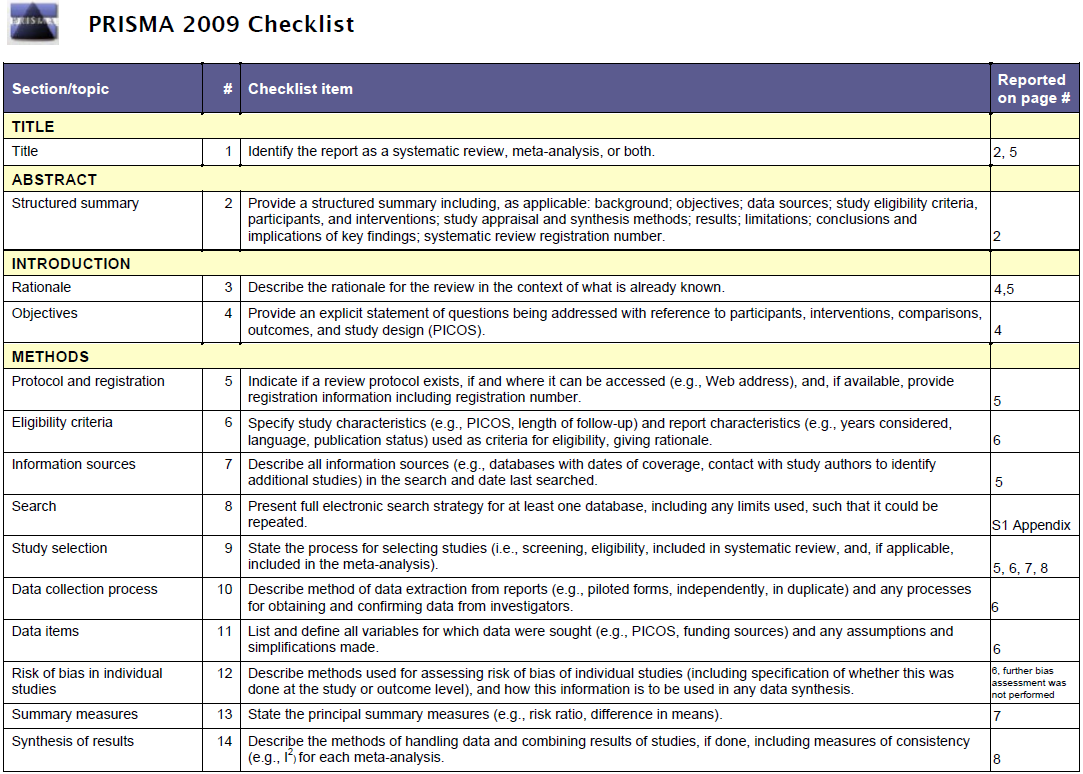

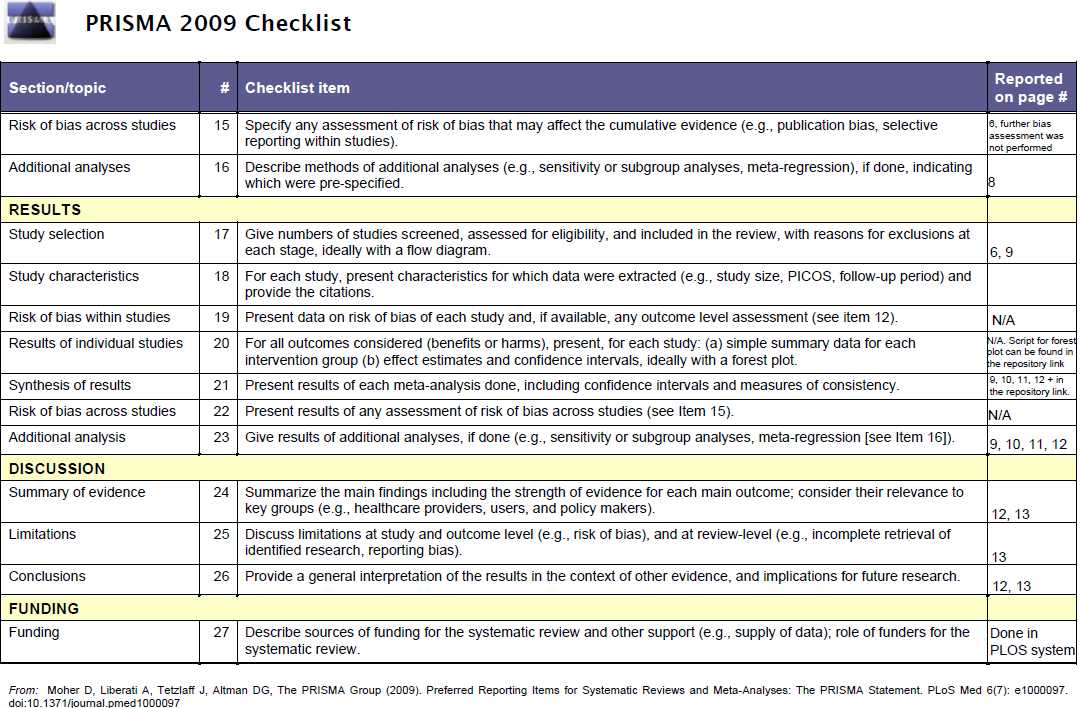


**S1 Table. Frequencies of each transmission route identified.**

| Transmission route | Frequency |
| --- | --- |
| Air to animal | 3 |
| Animal to air | 29 |
| Animal to water | 2 |
| Animal to animal | 14 |
| Animal to environment | 8 |
| Breast feeding | 17 |
| Contaminated room | 4 |
| Contact with infected person | 83 |
| Eating meat to human | 24 |
| Environment to animal | 1 |
| Environment to plant | 2 |
| Environment to environment | 2 |
| Environment to human | 2 |
| Family member colonised | 44 |
| Family member with occupational exposure | 11 |
| Fomites | 12 |
| Food to animal | 5 |
| Human to air | 6 |
| Human to nearby environment | 30 |
| Human to animal | 1 |
| Intervention | 17 |
| Livestock to drinking water | 12 |
| Milk to human | 1 |
| Animal mother to child | 3 |
| Mother to child during birth | 20 |
| Nearby environment to animal | 1 |
| Nearby environment to human | 4 |
| Nearby farm to human | 10 |
| Nearby farm to animal | 1 |
| Non-commercial animal keeping | 4 |
| Occupation exposure | 157 |
| Organ transfer | 3 |
| Other: animal to meat of animal | 1 |
| Other: bathing/showering | 3 |
| Other: crowding | 1 |
| Other: antibacterial soap | 3 |
| Pet to human | 28 |
| Prior colonised patient in room | 3 |
| Sharing water source with animals | 5 |
| Space sharing | 11 |
| Travelling | 110 |
| Pre-travel screening : 70 |  |
| No pre-travel screening: 40 |  |
| Travelling: eating food with local population | 1 |
| Contacting foreign healthcare service while travelling | 1 |
| Vegetables to human | 4 |
| Water to plant | 2 |
| Water to animal | 1 |
| Water (drinking) to human | 6 |
| Water (exposure) to human | 5 |
| Total | 718 |

**S2 Table.** **Frequencies of bacteria studied for transmission routes included in quantitative synthesis**

| Bacteria | Frequencies |
| --- | --- |
| *Acinetobacter baumannii* | 26 |
| *Acinetobacter calcoaceticus* | 4 |
| Campylobacter | 2 |
| *E. coli* | 160 |
| Enterobacteriaceae (unspecified)^1^ | 121 |
| *Enterococcus faecalis* | 2 |
| *Enterococcus faecium* | 4 |
| Group B streptococci | 6 |
| *S. aureus* | 275 |
| *Pseudomonas aeruginosa* | 13 |
| Salmonella | 4 |
| *Staphylococcus epidermidis* | 9 |
| *Staphylococcus haemolyticus* | 5 |
| *Staphylococcus pseudintermedius* | 7 |
| Staphylococci | 16 |
| *Streptococcus pneumoniae* | 10 |
| VRE | 42 |
| Other^2^ | 12 |
| Total | 718 |
| *Notes: ^1^Multiple Enterobacteriaceae or* *unspecified*, ^2^*Enterococci, Mycoplasma hominis, Ureaplasma urealyticum,* *Klebsiella pneumoniae,* *Staphylococcus hominis, Staphylococcus cohnii, Klebsiella oxytoca, Haemophilus influenzae, genes of bacterial species* | |

**S3 Table. List of studies of which estimates were included in the synthesis.**

| Author | Citation | Title | Transmission route | Bacteria group | Method of estimation |
| --- | --- | --- | --- | --- | --- |
| Rosen | (24) | Persistent and Transient Airborne MRSA Colonization of Piglets in a Newly Established Animal Model. | Air to animal | *S. aureus* | Bacteria load |
| Dierikx | (25) | Dynamics of cefotaxime resistant Escherichia coli in broilers in the first week of life. | Animal to animal | E. coli | Statistics: Risk |
| van Duijkeren | (26) | Transmission of methicillin-resistant Staphylococcus pseudintermedius between infected dogs and cats and contact pets, humans and the environment in households and veterinary clinics | Animal to animal, pet to human | Staphylococcus pseudintermedius | Statistics: Risk |
| Loeffler | (27) | Lack of transmission of methicillin-resistant Staphylococcus aureus (MRSA) between apparently healthy dogs in a rescue kennel | Animal to animal | *S. aureus* | Statistics: Risk |
| Manga | (28) | Fecal Carriage and Whole-Genome Sequencing-Assisted Characterization of CMY-2 Beta-Lactamase-Producing Escherichia coli in Calves at Czech Dairy Cow Farm | Animal to animal | *E. coli* | Genes |
| Broens | (16) | Quantification of transmission of livestock-associated methicillin resistant Staphylococcus aureus in pigs. | Animal to animal | *S. aureus* | Modelling: R0 |
| Huijbers | (29) | Transmission dynamics of extended-spectrum β-lactamase and AmpC β-lactamase-producing Escherichia coli in a broiler flock without antibiotic use | Animal to animal, environment to animal | *E. coli* | Modelling: Transmission rate, R0 |
| Riccobono | (30) | Carriage of Antibiotic-Resistant Escherichia coli Among Healthy Children and Home-Raised Chickens: A Household Study in a Resource-Limited Setting | Animal to animal, occupational exposure, contact with infected person | *E. coli* | Genes |
| Weese | (31) | Suspected transmission of methicillin-resistant Staphylococcus aureus between domestic pets and humans in veterinary clinics and in the household | Animal to animal, pet to human | *S. aureus* | Genes |
| Windahl | (32) | Colonization with methicillin-resistant Staphylococcus pseudintermedius in multi-dog households: A longitudinal study using whole genome sequencing | Animal to animal | *Staphylococcus pseudintermedius* | Genes |
| Graveland | (33) | Dynamics of MRSA carriage in veal calves: a longitudinal field study. | Animal to animal, Animal to air | *S. aureus* | Statistics: Transmission rate (week) |
| Gao | (34) | Emissions of Escherichia coli Carrying Extended-Spectrum β-Lactamase Resistance from Pig Farms to the Surrounding Environment | Animal to environment, animal to air, animal to water | *E. coli* | Statistics: Risk |
| Laube | (35) | Transmission of ESBL/AmpC-producing Escherichia coli from broiler chicken farms to surrounding areas. | Animal to environment, animal to air | *E. coli* | Statistics: Risk |
| Moodley | (36) | Transmission of IncN plasmids carrying blaCTX-M-1 between commensal Escherichia coli in pigs and farm workers. | Animal to environment, animal to air, occupational exposure | *E. coli* | Statistics: Risk |
| Gao | (37) | Application of swine manure on agricultural fields contributes to extended-spectrum β-lactamase-producing Escherichia coli spread in Tai’an, China | Animal to environment | *E. coli* | Genes |
| Leite-Martins | (38) | Spread of multidrug-resistant Enterococcus faecalis within the household setting. | Animal to environment, occupational exposure | *Enterococcus faecalis* | Genes |
| Schmithausen | (39) | Analysis of Transmission of MRSA and ESBL-E among Pigs and Farm Personnel | Animal to air, occupational exposure | *S. aureus*, Enterobacteriaceae | Statistics: Risk |
| Davis | (40) | Occurrence of Staphylococcus aureus in swine and swine workplace environments on industrial and antibiotic-free hog operations in North Carolina, USA: A One Health pilot study | Animal to air, occupational exposure | *S. aureus* | Statistics: Risk |
| Fu | (41) | Aquatic animals promote antibiotic resistance gene dissemination in water via conjugation: Role of different regions within the zebra fish intestinal tract, and impact on fish intestinal microbiota | Animal to water | *E. coli* | Bacteria intake |
| Parm | (42) | Risk factors associated with gut and nasopharyngeal colonization by common Gram-negative species and yeasts in neonatal intensive care units patients | Breast feeding | Other | Statistics: OR |
| Chen | (43) | Factors associated with nasal colonization of methicillin-resistant Staphylococcus aureus among healthy children in Taiwan. | Breast feeding | *S. aureus* | Statistics: OR |
| Isendahl | (44) | Fecal carriage of ESBL-producing E. coli and K. pneumoniae in children in Guinea-Bissau: a hospital-based cross-sectional study. | Breast feeding, fomites | Enterobacteriaceae | Statistics: OR |
| Hijazi | (45) | Multidrug-resistant ESBL-producing Enterobacteriaceae and associated risk factors in community infants in Lebanon | Breast feeding, pet to human | Enterobacteriaceae | Statistics: OR |
| Koliou | (46) | Risk factors for carriage of Streptococcus pneumoniae in children. | Breast feeding | Streptococcus pneumoniae | Statistics: OR |
| Nakamura | (47) | Outbreak of extended-spectrum β-lactamase-producing Escherichia coli transmitted through breast milk sharing in a neonatal intensive care unit | Breast feeding | *E. coli* | Statistics: OR and genes |
| Nordberg | (48) | High Proportion of Intestinal Colonization with Successful Epidemic Clones of ESBL-Producing Enterobacteriaceae in a Neonatal Intensive Care Unit in Ecuador | Breast feeding | *E. coli* | Statistics: OR |
| Ciftçi | (49) | Investigation of risk factors for penicillin-resistant Streptococcus pneumoniae carriage in Turkish children. | Breast feeding, other crowding | Streptococcus pneumoniae | Statistics: OR |
| Gastelum | (50) | Transmission of community-associated methicillin-resistant Staphylococcus aureus from breast milk in the neonatal intensive care unit. | Breast feeding, contact with infected person | *S. aureus* | Statistics: Risk and genes |
| Benito | (51) | Characterization of Staphylococcus aureus strains isolated from faeces of healthy neonates and potential mother-to-infant microbial transmission through breastfeeding | Breast feeding | *S. aureus* | Genes^rs^ |
| Gueimonde | (52) | Presence of specific antibiotic (tet) resistance genes in infant faecal microbiota. | Breast feeding | Genes | Genes |
| Jackson | (53) | Bacterial burden is associated with increased transmission to health care workers from patients colonized with vancomycin-resistant Enterococcus | Contact with infected person | VRE | Genes |
| Morgan | (54) | Frequent Multidrug-Resistant Acinetobacter baumannii Contamination of Gloves, Gowns, and Hands of Healthcare Workers | Contact with infected person, space sharing | *Acinetobacter baumannii* | Statistics: OR, risk |
| Schwartz-Neiderman | (55) | Risk Factors for Carbapenemase-Producing Carbapenem-Resistant Enterobacteriaceae (CP-CRE) Acquisition Among Contacts of Newly Diagnosed CP-CRE Patients | Contact with infected person, space sharing | Enterobacteriaceae | Statistics: OR |
| Nerby | (12) | Risk factors for household transmission of community-associated methicillin-resistant Staphylococcus aureus | Contact with infected person, space sharing, other soap, fomites, family member colonized, | *S. aureus,* VRE | Statistics: OR and genes |
| Grabsch | (56) | Risk of Environmental and Healthcare Worker Contamination With Vancomycin-Resistant Enterococci During Outpatient Procedures and Hemodialysis | Contact with infected person, human to nearby environment | VRE | Statistics: Risk |
| Snyder | (57) | Detection of methicillin-resistant Staphylococcus aureus and vancomycin-resistant enterococci on the gowns and gloves of healthcare workers. | Contact with infected person | *S. aureus*, VRE | Statistics: Risk |
| El Shafie | (58) | Investigation of an outbreak of multidrug-resistant *Acinetobacter baumannii* in trauma intensive care unit | Contact with infected person, human to nearby environment | *Acinetobacter baumannii* | Statistics: Risk |
| Senn | (59) | The Stealthy Superbug: the Role of Asymptomatic Enteric Carriage in Maintaining a Long-Term Hospital Outbreak of ST228 Methicillin-Resistant Staphylococcus aureus. | Contact with infected person | *S. aureus* | Statistics: Risk |
| Rydberg | (60) | Intrafamilial spreading of Escherichia coli resistant to trimethoprim | Contact with infected person | *E. coli* | Statistics: Risk and genes |
| Frénay | (61) | Long-term carriage, and transmission of methicillin-resistant Staphylococcus aureus after discharge from hospital. | Contact with infected person | *S. aureus* | Statistics: Risk |
| McBryde | (62) | An investigation of contact transmission of methicillin-resistant Staphylococcus aureus | Contact with infected person | *S. aureus* | Statistics: Risk |
| Jackson | (53) | Bacterial burden is associated with increased transmission to health care workers from patients colonized with vancomycin-resistant Enterococcus | Contact with infected person | VRE | Statistics: Risk |
| Mutters | (8) | Low risk of apparent transmission of vancomycin-resistant Enterococci from bacteraemicpatients to hospitalized contacts. | Contact with infected person | VRE | Statistics: Risk and genes |
| Schaumburg | (63) | Transmission of Staphylococcus aureus between mothers and infants in an African setting | Mother to child | *S. aureus* | Statistics: Risk^rs^, OR^rs^ |
| Tandé | (7) | Intrafamilial transmission of extended-spectrum-beta-lactamase-producing Escherichia coli and Salmonella enterica Babelsberg among the families of internationally adopted children. | Family member colonized | Enterobacteriaceae | Statistics: Risk and genes |
| Torres | (64) | Prevalence and transmission dynamics of Escherichia coli ST131 among contacts of infected community and hospitalized patients. | Contact with infected person | *E. coli* | Statistics: Risk |
| Zhou | (65) | Factors Associated with acquisition of vancomycin-resistant Enterococci(VRE) in roommate contacts of patients colonized or infected with VRE in a tertiary care hospital | Contact with infected person | VRE | Statistics: Risk |
| Hedin | (66) | Multiply antibiotic-resistant *Staphylococcus epidermidis* in patients, staff and environment- a one-week survey in a bone marrow transplant unit. | Contact with infected person, human to air | *Staphylococcus epidermidis* | Statistics: Risk |
| Pierce | (67) | Methicillin-resistant *Staphyloccus areus* (MRSA) acquisition risk in an endemic neonatal intensive care unit with an active surveillance culture and decolonization programme | Contact with infected person | *S. aureus* | Statistics: RR |
| Calfee | (68) | Spread of methicillin-resistant Staphylococcus aureus (MRSA) among household contacts of individuals with nosocomially acquired MRSA. | Contact with infected person | *S. aureus* | Statistics: RR |
| Domenech de Cellès | (69) | Identifying more epidemic clones during a hospital outbreak of multidrug-resistant *Acinetobacter baumannii* | Contact with infected person | *Acinetobacter baumannii* | Modelling: R0 |
| Christopher | (70) | Transmission dynamics of methicillin-resistant *Staphylococcus aureus* in a medical intensive care unit in India | Contact with infected person | *S. aureus* | Modelling: R0 |
| Austin | (71) | Vancomycin-resistant enterococci in intensive-care hospital settings: transmission dynamics, persistence, and the impact of infection control programs. | Contact with infected person | VRE | Modelling: R0 |
| Plipat | (72) | The dynamics of methicillin-resistant Staphylococcus aureus exposure in a hospital model and the potential for environmental intervention. | Contact with infected person, Nearby environment to human | *S. aureus* | Modelling: Importance of route |
| Mikolajczyk | (73) | Mixture model to assess the extent of cross-transmission of multidrug-resistant pathogens in hospitals | Contact with infected person | *S. aureus*, *Acinetobacter baumannii*, Pseudomonas aeruginosa | Modelling: Importance of route |
| Cheah | (74) | Mathematical modelling of vancomycin-resistant enterococci transmission during passive surveillance and active surveillance with contact isolation highlights the need to identify and address the source of acquisition | Contact with infected person | VRE | Modelling: Importance of route |
| Cooper | (75) | Quantifying type-specific reproduction numbers for nosocomial pathogens: evidence for heightened transmission of an Asian sequence type 239 MRSA clone | Contact with infected person | *S. aureus* | Modelling: Acquisition rate |
| Domenech de Cellès | (76) | Intrinsic epidemicity of Streptococcus pneumoniae depends on strain serotype and antibiotic susceptibility pattern | Contact with infected person | Streptococcus pneumoniae | Modelling: Transmission rate |
| Forrester | (77) | Use of stochastic epidemic odelling to quantify transmission rates of colonization with methicillin-resistant Staphylococcus aureus in an intensive care unit | Contact with infected person, nearby environment to human | *S. aureus* | Modelling: Cases per day |
| Forrester | (78) | Bayesian inference of hospital-acquired infections and control measures given imperfect surveillance data | Contact with infected person | *S. aureus* | Modelling: Cases per day |
| Hetem | (79) | Nosocomial transmission of community-associated methicillin-resistant Staphylococcus aureus in Danish Hospitals | Contact with infected person, nearby environment to human | *S. aureus* | Modelling: R0 |
| Mikolajczyk | (80) | A statistical method for estimating the proportion of cases resulting from cross-transmission of multi-resistant pathogens in an intensive care unit. | Contact with infected person | *S. aureus*, Pseudomonas aeruginosa | Modelling: Importance of route |
| Simon | (81) | Modeling bacterial colonization and infection routes in health care settings: analytic and numerical approaches. | Contact with infected person | *S. aureus* | Modelling: R0 |
| López-García | (82) | A unified stochastic modelling framework for the spread of nosocomial infections | Contact with infected person, human to nearby environment | *S. aureus*, VRE | Modelling: R0 |
| McBryde | (83) | A stochastic mathematical model of methicillin resistant Staphylococcus aureus transmission in an intensive care unit: predicting the impact of interventions | Contact with infected person | *S. aureus* | Modelling: R0, transmission rate |
| Pelupessy | (84) | How to assess the relative importance of different colonization routes of pathogens within hospital settings | Contact with infected person | VRE, Pseudomonas aeruginosa | Modelling: transmission rate |
| Wang | (85) | Modeling nosocomial infections of methicillin-resistant *Staphylococcus aureus* with environment contamination | Contact with infected person | *S. aureus* | Modelling: R0, transmission rate |
| Lo | (86) | Fecal carriage of CTXM type extended-spectrum beta-lactamase-producing organisms by children and their household contacts. | Family member colonize, contact with infected person | Enterobacteriaceae | Genes |
| Givon-Lavi | (87) | Spread of Streptococcus pneumoniae and antibiotic-resistant S. pneumoniae from day-care center attendees to their younger siblings | Family member colonized | Streptococcus pneumoniae | Genes^rs^ |
| Alam | (88) | Transmission and Microevolution of USA300 MRSA in U.S. Households: Evidence from Whole-Genome Sequencing | Family member colonized | *S. aureus* | Genes |
| Alves | (89) | Extended-spectrum beta-lactamase-producing Enterobacteriaceae in the intensive care unit: acquisition does not mean cross-transmission | Contact with infected person | Enterobacteriaceae | Genes |
| Chidekel | (90) | Nasopharyngeal colonization in children with cystic fibrosis: Antibiotic resistance and intrafamilial spread | Family member colonized | *S. aureus*, Streptococcus pneumoniae, other | Genes^rs^ |
| Cochard | (91) | Extended-spectrum β-lactamase-producing Enterobacteriaceae in French nursing homes: an association between high carriage rate among residents, environmental contamination, poor conformity with good hygiene practice, and putative resident-to-resident trans | Contact with infected person, human to nearby environment | Enterobacteriaceae | Genes |
| Crombach | (92) | Control of an epidemic spread of a multi-resistant strain of *Acinetobacter calcoaceticus* in a hospital | Contact with infected person, human to air, human to nearby environment | Acinetobacter calcoaceticus | Genes |
| Davis | (21) | Genome sequencing reveals strain dynamics of methicillin-resistant *Staphylococcus aureus* in the same household in the context of clinical disease in a person and a dog | Family member colonized, pet to human, nearby environment to animal | *S. aureus* | Genes |
| Cuny | (93) | Nasal colonization of humans with methicillin-resistant *Staphylococcus aureus* (MRSA) CC398 with and without exposure to pigs | Contact with infected person | *S. aureus* | Genes |
| Eveillard | (94) | Carriage of methicillin-resistant *Staphylococcus aureus* among hospital employees: prevalence, duration, and transmission to households | Family member colonized | *S. aureus* | Genes |
| Griffith | (95) | The epidemiology of *Pseudomonas aeruginosa* in oncology patients in a general hospital | Contact with infected person, environment to human | *Pseudomonas aeruginosa* | Genes |
| Gustafsson | (96) | MRSA in children from foreign countries adopted to Swedish families | Family member colonized | *S. aureus* | Genes |
| Henriqus Normark | (97) | Clonal analysis of *Streptococcus pneumoniae* nonsusceptible to penicillin at day-care centers with index cases, in a region with low incidence of resistance: emergence of an invasive type 35B clone among carriers | Contact with infected person | Streptococcus pneumoniae | Genes |
| Johansson | (98) | High prevalence of MRSA in household contacts. | Family member colonized | *S. aureus* | Genes |
| Johnson | (99) | The role of patient-to-patient transmission in the acquisition of imipenem-resistant *Pseudomonas aeruginosa* colonization in the intensive care unit | Contact with infected person | Pseudomonas aeruginosa | Genes |
| Kojima | (100) | Spread of CTX-M-15 Extended-Spectrum-Lactamase-Producing *Escherichia coli* isolates through household contact and plasmid transfer | Contact with infected person | *E. coli* | Genes |
| Maataoui | (101) | High acquisition rate of extended-spectrum β-lactamase-producing Enterobacteriaceae among French military personnel on mission abroad, without evidence of inter-individual transmission. | Contact with infected person, travelling + | *E. coli* | Genes and Statistics: OR, risk |
| McCallum | (13) | Spread of an epidemic *Pseudomonas aeruginosa* strain from a patient with cystic fibrosis (CF) to non-CF relatives | Family member colonized | Pseudomonas aeruginosa | Genes |
| Mitsuda | (102) | The influence of methicillin-resistant *Staphylococcus aureus* (MRSA) carriers in a nursery and transmission of MRSA to their households | Contact with infected person, family member colonized | *S. aureus* | Genes |
| Ozaki | (14) | Genotypes, intrafamilial transmission, and virulence potential of nasal methicillin-resistant *Staphylococcus aureus* from children in the community. | Contact with infected person, family member colonized | *S. aureus* | Genes |
| Stone | (103) | Methicillin-resistant *Staphylococcus aureus* (MRSA) nasal carriage in residents of Veterans Affairs long-term care facilities: role of antimicrobial exposure and MRSA acquisition. | Contact with infected person | *S. aureus* | Genes |
| Toleman | (104) | Systematic surveillance detects multiple silent introductions and household transmission of methicillin-resistant *Staphylococcus aureus* USA300 in the east of England. | Family member colonized | *S. aureus* | Genes |
| Kariuki | (105) | Invasive multidrug-resistant non-typhoidal Salmonella infections in Africa: zoonotic or anthroponotic transmission? | Contact with infected person, Environment to human | Salmonella | Genes |
| Geffers | (106) | Risk of Transmission of Nosocomial Methicillin-Resistant Staphylococcus aureus (MRSA) From Patients Colonized With MRSA | Contact with infected person | *S. aureus* | Statistics: transmission rate |
| Martínez | (107) | Role of environmental contamination as a risk factor for acquisition of vancomycin-resistant enterococci in patients treated in a medical intensive care unit | Contaminated room | VRE | Statistics: OR |
| Salm | (108) | Prolonged outbreak of clonal MDR *Pseudomonas aeruginosa* on an intensive care unit: contaminated sinks and contamination of ultra-filtrate bags as possible route of transmission? | Contaminated room | *Pseudomonas aeruginosa* | Statistics: OR |
| Leistner | (109) | Risk factors associated with the community-acquired colonization of extended-spectrum beta-lactamase (ESBL) positive *Escherichia Coli*. An exploratory case-control study. | Eating Meat to human | *E. coli* | Statistics: OR |
| Lietzau | (110) | Clustering of antibiotic resistance of *E. coli* in couples: suggestion for a major role of conjugal transmission | Eating Meat to human, family member colonized | *E. coli* | Statistics: OR |
| Nadimpalli | (111) | Meat and fish as sources of Extended-Spectrum β-Lactamase–Producing *Escherichia coli*, Cambodia | Eating meat to human, pet to human, vegetables to human | *E. coli* | Statistics: OR |
| Samore | (112) | Impact of outpatient antibiotic use on carriage of ampicillin-resistant *Escherichia coli*? | Eating Meat to human, travelling -, occupational exposure | *E. coli* | Statistics: PR |
| Börjesson | (113) | Limited dissemination of Extended-Spectrum β-Lactamase- and Plasmid-Encoded AmpC-Producing *Escherichia coli* from food and farm animals, Sweden | Eating Meat to human, occupational exposure | *E. coli* | Genes |
| Sørensen | (114) | Transient intestinal carriage after ingestion of antibiotic-resistant Enterococcus faecium from chicken and pork. | Eating Meat to human | Enterococcus faecium | Bacteria intake |
| Duckro | (115) | Transfer of vancomycin-resistant enterococci via health care worker hands | Environment to environment, human to nearby environment | VRE | Statistics: Risk |
| Kotay | (116) | Spread from the sink to the patient: In situ study using green fluorescent protein (GFP)- expressing *Escherichia coli* to model bacterial dispersion from hand-washing sink-trap reservoirs | Environment to environment | *E. coli* | Bacteria intake |
| Habteselassie | (117) | Understanding the role of agricultural practices in the potential colonization and contamination by *Escherichia coli* in the rhizospheres of fresh produce | Environment to Plant, water to Plant | *E. coli* | Bacteria intake |
| Ferreira | (118) | Transmission of MRSA between companion animals and infected human patients presenting to outpatient medical care facilities | Family member colonized | *S. aureus* | Statistics: OR |
| Fornasini | (119) | Trimethoprim-resistant *Escherichia coli* in households of children attending day care centers. | Family member colonized | *E. coli* | Statistics: OR |
| Samore | (120) | High rates of multiple antibiotic resistance in Streptococcus pneumoniae from healthy children living in isolated rural communities: association with cephalosporin use and intrafamilial transmission. | Family member colonized | Streptococcus pneumoniae | Statistics: OR |
| Cluzet | (121) | Risk factors for recurrent colonization with methicillin-resistant *Staphylococcus aureus* in community-dwelling adults and children | Family member colonized | *S. aureus* | Statistics: OR |
| Stewardson | (122) | Effect of outpatient antibiotics for urinary tract infections on antimicrobial resistance among commensal Enterobacteriaceae: a multinational prospective cohort study. | Family member colonized, travelling - | Enterobacteriaceae | Statistics: PR |
| Paltansing | (123) | Extended-spectrum β-lactamase-producing Enterobacteriaceae among travelers from the Netherlands. | Family member colonized, travelling + | *E. coli* | Statistics: Risk |
| Zetsma | (124) | Carrier state and spread of methicillin resistant *Staphylococcus aureus* following hospital discharge | Family member colonized, pet to human | *S. aureus* | Statistics: Risk |
| Garcia-Graells | (125) | Livestock veterinarians at high risk of acquiring methicillin-resistant *Staphylococcus aureus* ST398 | Family member occupational exposure, occupational exposure | *S. aureus* | Statistics: OR |
| Lo | (126) | Dissemination of methicillin-resistant *Staphylococcus aureus* among healthy children in Northern Taiwan | Family member occupational exposure | *S. aureus* | Statistics: OR |
| Lu | (127) | Methicillin-resistant *Staphylococcus aureus* carriage, infection and transmission in dialysispatients, healthcare workers and their family members | Family member occupational exposure | *S. aureus* | Statistics: OR |
| Neyra | (128) | Multidrug-resistant and methicillin-resistant *Staphylococcus aureus* (MRSA) in hog slaughter and processing plant workers and their community in North Carolina (USA) | Family member occupational exposure, occupational Exposure | *S. aureus* | Statistics: PR |
| Würtz | (129) | No apparent transmission of livestock-associated methicillin-resistant *Staphylococcus aureus* CC398 in a survey of staff at a regional Danish hospital | Family member occupational exposure | *S. aureus* | Statistics: Risk |
| Baran | (130) | Stool colonization with vancomycin-resistant Enterococci in healthcare workers and their households | Family member occupational exposure | VRE | Statistics: Risk |
| Maree | (131) | Risk factors for infection and colonization with community-associated methicillin-resistant *Staphylococcus aureus* in the Los Angeles county jail: A case-control study | Fomites, other Bathing/Showering | *S. aureus* | Statistics: OR |
| Nilsson | (132) | Carriage of penicillin-resistant *Streptococcus pneumoniae* by children in day-care centers during an intervention program in Malmo, Sweden | Fomites | Streptococcus pneumoniae | Statistics: OR |
| Rackham | (133) | Community-associated methicillin-resistant *Staphylococcus aureus* nasal carriage in a college student athlete population | Fomites | *S. aureus* | Statistics: Risk |
| Groat | (134) | Canine raw meat diets and antimicrobial resistant *E. coli*: is there a link? | Food to animal | *E. coli* | Statistics: OR |
| Olkkola | (135) | Population genetics and antimicrobial susceptibility of canine Campylobacter isolates collected before and after a raw feeding experiment. | Food to animal | Campylobacter | Statistics: Risk |
| Fukuda | (136) | Role of flies in the maintenance of antimicrobial resistance in farm environments | Food to animal | *E. coli* | Bacteria Intake |
| Barbarin | (137) | Colonization of Cimex lectularius with methicillin-resistant *Staphylococcus aureus*. | Human to animal | *S. aureus* |  |
| Lis | (138) | Methicillin resistance of airborne coagulase-negative staphylococci in homes of persons having contact with a hospital environment. | Human to air | *Staphylococcus epidermidis*, other | Statistics: Risk difference |
| Kennedy | (139) | Face mask sampling reveals antimicrobial resistance genes in exhaled aerosols from patients with chronic obstructive pulmonary disease and healthy volunteer | Human to air | Genes | Statistics: Risk |
| Thom | (140) | Environmental contamination due to multidrug-resistant *Acinetobacter baumannii* surrounding colonized or infected patients | Human to nearby environment | Enterobacteriaceae | Statistics: Risk |
| Bures | (141) | Computer keyboards and faucet handles as reservoirs of nosocomial pathogens in the intensive care unit. | Human to nearby environment | *S. aureus* | Statistics: Risk |
| Shahbazian | (142) | Multidrug and mupirocin resistance in environmental methicillin-resistant *Staphylococcus aureus* (MRSA) isolates from homes of people diagnosed with community-onset MRSA infection. | Human to nearby environment | *S. aureus* | Statistics: Risk |
| Levin | (143) | Environmental contamination by multidrug-resistant *Acinetobacter baumannii* in an intensive care unit. | Human to nearby environment | *Acinetobacter baumannii* | Statistics: Risk |
| Zimbudzi | (144) | Contamination of renal patients’ hospital chart covers with vancomycin-resistant enterococci: Handle with care | Human to nearby environment | VRE | Statistics: Risk and genes |
| Chalfine | (145) | Ten-year decrease of acquired methicillin-resistant *Staphylococcus aureus* (MRSA) bacteremia at a single institution: the result of a multifaceted program combining cross-transmission prevention and antimicrobial stewardship. | Intervention | *S. aureus* | Statistics: Risk |
| Duggal | (146) | An investigation of MRSA from the burns ward the importance of hand hygiene | Intervention | *S. aureus* | Statistics: Risk |
| Batra | (147) | Efficacy and limitation of a chlorhexidine-based decolonization strategy in preventing transmission of methicillin-resistant *Staphylococcus aureus* in an intensive care unit | Intervention | *S. aureus* | Modelling: Incidence rate ratios |
| D’Agata | (148) | The impact of persistent gastrointestinal colonization on the transmission dynamics of vancomycin-resistant enterococci. | Intervention | VRE | Modelling: Importance of Route |
| Perencevich | (149) | Projected benefits of active surveillance for vancomycin-resistant enterococci in intensive care units | Intervention | VRE | Modelling: Incidence |
| Montecalvo | (150) | Infection-control measures reduce transmission of vancomycin-resistant enterococci in an endemic setting. | Intervention | VRE | Modelling: transmission rate |
| Coleman | (151) | Contamination of Canadian private drinking water sources with antimicrobial resistant *Escherichia coli*. | Livestock to drinking water, occupational Exposure | *E. coli* | Statistics: OR |
| Caudell | (152) | Identification of risk factors associated with carriage of resistant Escherichia coli in three culturally diverse ethnic groups in Tanzania: a biological and socioeconomic analysis | Milk to human, non -commercial animal keeping, sharing water with animal | *E. coli* | Statistics: OR |
| Moodley | (153) | Experimental colonization of pigs with methicillin-resistant Staphylococcus aureus (MRSA): insights into the colonization and transmission of livestock-associated MRSA. | Mother (animal) to child | *S. aureus* | Statistics: Risk |
| Corrò | (154) | Occurrence and characterization of methicillin-resistant *Staphylococcus pseudintermedius* in successive parturitions of bitches and their puppies in two kennels in Italy. | Mother (animal) to child | *Staphylococcus pseudintermedius* | Genes |
| Tamelienė | (155) | *Escherichia coli* colonization in neonates: prevalence. perinatal transmission. antimicrobial susceptibility. and risk factors. | Mother to child | *E. coli* | Statistics: OR^rs^ |
| Denkel | (156) | The mother as most important risk factor for colonization of very low birth weight (VLBW) infants with extended-spectrum β-lactamase-producing Enterobacteriaceae (ESBL-E). | Mother to child | Enterobacteriaceae, *S. aureus* | Statistics: OR, risk^rs^ |
| Kunze | (157) | Colonization, serotypes and transmission rates of group B streptococci in pregnant women and their infants born at a single University Center in Germany. | Mother to child | Group B streptococci | Statistics: Risk^rs^ |
| Bourgeois-Nicolaos | (158) | Maternal vaginal colonisation by *Staphylococcus aureus* and newborn acquisition at delivery. | Mother to child | *S. aureus* | Statistics: Risk |
| Chua | (159) | Ureaplasma urealyticum and Mycoplasma hominis isolation from cervical secretions of pregnant women and nasopharyngeal secretions of their babies at delivery | Mother to child | Other | Statistics: Risk^rs^ |
| Eren | (160) | The carriage of group B streptococci in Turkish pregnant women and its transmission rate in newborns and serotype distribution | Mother to child | Group B streptococci | Statistics: Risk^rs^ |
| Facchinetti | (161) | Chlorhexidine vaginal flushings versus systemic ampicillin in the prevention of vertical transmission of neonatal group B streptococcus, at term | Mother to child | Group B streptococci | Statistics: Risk^rs^ |
| Jiménez-Rámila | (162) | Vagino-rectal colonization and maternal-neonatal transmission of Enterobacteriaceae producing extended-spectrum β-lactamases or carbapenemases: a cross-sectional study. | Mother to child | Enterobacteriaceae | Statistics: Risk |
| Kadanali | (163) | Maternal carriage and neonatal colonisation of group B streptococcus in eastern Turkey: prevalence, risk factors and antimicrobial resistance | Mother to child | Group B streptococci | Statistics: Risk^rs^ |
| Kunze | (164) | Comparison of pre- and intrapartum screening of group B streptococci and adherence to screening guidelines: a cohort study. | Mother to child | Group B streptococci | Statistics: Risk^rs^ |
| Strus | (165) | Group B streptococcus colonization of pregnant women and their children observed on obstetric and neonatal wards of the University Hospital in Krakow, Poland. | Mother to child | Group B streptococci | Statistics: Risk |
| Kothari | (166) | Community acquisition of beta-lactamase producing Enterobacteriaceae in neonatal gut | Mother to child | Enterobacteriaceae | Statistics: Risk |
| Morel | (167) | Nosocomial transmission of methicillin-resistant *Staphylococcus aureus* from a mother to her preterm quadruplet infants | Mother to child | *S. aureus* | Genes |
| Peretz | (168) | Peripartum maternal transmission of extended-spectrum β-lactamase organism to newborn infants | Mother to child | Enterobacteriaceae | Genes |
| Kurita | (169) | Nosocomial transmission of methicillin-resistant *Staphylococcus aureus* via the surfaces of the dental operatory. | Nearby environment to human | *S. aureus* | Genes |
| Zhou | (170) | Sources of sporadic *Pseudomonas aeruginosa* colonizations/infections in surgical ICUs: Association with contaminated sink trap. | Nearby environment to human | *Pseudomonas aeruginosa* | Genes^rs^ |
| Rubini | (171) | Prevalence of Salmonella strains in wild animals from a highly populated area of north-eastern Italy | Nearby farm to Animal | Salmonella | Statistics: OR^rs^ |
| Wielders | (172) | Extended-spectrum β-lactamase- and pAmpC-producing Enterobacteriaceae among the general population in a livestock-dense area | Nearby farm to human, non-commercial animal keeping, occupational exposure, pet to human, travelling - | Enterobacteriaceae | Statistics: OR |
| Reynaga | (173) | Clinical impact and prevalence of MRSA CC398 and differences between MRSA-TetR and MRSA-TetS in an area of Spain with a high density of pig farming: a prospective cohort study. | Occupational Exposure | *S. aureus* | Statistics: OR |
| Diercke | (174) | Livestock-associated methicillin-resistant Staphylococcus aureus in patients admitted to hospitals in the Ems-Dollart-Region, 2011 | Occupational Exposure | *S. aureus* | Statistics: OR and genes |
| Founou | (175) | Mannitol-fermenting methicillin-resistant staphylococci (MRS) in pig abatoirs in Cameroon and South Africa: A serious foodsafety threat. | Occupational Exposure | *S. aureus,* Staphylococci | Statistics: OR, risk |
| Ho | (176) | Occupational exposure to raw meat: a newly-recognized risk factor for *Staphylococcus aureus* nasal colonization amongst food handlers | Occupational Exposure | *S. aureus* | Statistics: OR, OR^rs^ |
| Jayaweera | (177) | Antibiotic resistance patterns of methicillin-resistant *Staphylococcus aureus* (MRSA) isolated from livestock and associated farmers in Anuradhapura, Sri Lanka | Occupational Exposure | *S. aureus* | Statistics: OR |
| Dohmen | (17) | Carriage of extended-spectrum β-lactamases in pig farmers is associated with occurrence in pigs | Occupational Exposure | Enterobacteriaceae | Statistics: OR, risk and genes |
| Mascaro | (19) | Prevalence of livestock-associated methicillin-resistant *Staphylococcus aureus* (LA-MRSA) among farm and slaughterhouse workers in Italy | Occupational Exposure | *S. aureus* | Statistics: OR, risk |
| Moodley | (178) | High risk for nasal carriage of methicillin-resistant Staphylococcus aureus among Danish veterinary practitioners | Occupational Exposure | *S. aureus* | Statistics: OR |
| Rasamiravaka | (20) | Evaluation of methicillin-resistant Staphylococcus aureus nasal carriage in Malagasy pig and poultry non-industrial farmers | Occupational Exposure | *S. aureus* | Statistics: OR^rs^ |
| Rasamiravaka | (179) | Evaluation of methicillin-resistant staphylococcus aureus nasal carriage in Malagasy veterinary students | Occupational Exposure | *S. aureus* | Statistics: OR |
| Rosenberg Goldstein | (180) | Occupational exposure to *Staphylococcus aureus* and Enterococcus spp. among spray irrigation workers using reclaimed water. | Occupational Exposure | *S. aureus* | Statistics: OR |
| van Rijen | (181) | Increase in a Dutch hospital of methicillin-resistant Staphylococcus aureus related to animal farming | Occupational Exposure, travelling (healthcare system contact) - | *S. aureus* | Statistics: OR |
| Ye | (182) | Livestock-associated methicillin and multidrug resistant *S. aureus* in humans is associated with occupational pig contact, not pet contact | Occupational Exposure, pet to human | *S. aureus* | Statistics: OR, PR |
| Ye | (183) | Genotypic and phenotypic markers of livestock-associated methicillin-resistant *Staphylococcus aureus* CC9 in humans | Occupational Exposure | *S. aureus* | Statistics: OR |
| Price | (184) | Elevated risk of carrying gentamicin-resistant *Escherichia coli* among U.S. poultry workers | Occupational Exposure | *E. coli* | Statistics: OR |
| Trung | (185) | Zoonotic transmission of mcr-1 colistin resistance gene from small-scale poultry Farms, Vietnam | Occupational Exposure | *E. coli* | Statistics: OR |
| Moser | (186) | The role of mobile genetic elements in the spread of antimicrobial-resistant *Escherichia coli* From chickens to humans in small-scale production poultry operations in rural Ecuador | Occupational Exposure | *E. coli* | Statistics: OR |
| Wardyn | (187) | Swine farming Is a risk factor for infection with and high prevalence of carriage of multidrug-resistant *Staphylococcus aureus* | Occupational exposure, family member occupational exposure | *S. aureus* | Statistics: PR^rs^, OR^rs^ |
| Rinsky | (188) | Livestock-associated methicillin and multidrug resistant *Staphylococcus aureus* is present among industrial, not antibiotic-free livestock operation workers in North Carolina | Occupational Exposure | *S. aureus* | Statistics: PR |
| Hatcher | (189) | The prevalence of antibiotic-resistant *Staphylococcus aureus* nasal carriage among industrial hog operation workers, community residents, and children living in their households: North Carolina, USA. | Occupational Exposure | *S. aureus* | Statistics: PR |
| Li | (18) | Nasal carriage of methicillin-resistant coagulase-negative staphylococci in healthy humans is associated with occupational pig contact in a dose-response manner | Occupational exposure | Staphylococci, *Staphylococcus epidermidis*, *Staphylococcus haemolyticus* | Statistics: PR |
| Borgen | (190) | Continuing high prevalence of VanA‐type vancomycin‐resistant enterococci on Norwegian poultry farms three years after avoparcin was banned | Occupational exposure | VRE | Statistics: PR |
| Fischer | (191) | Nasal colonization of pig-exposed persons with Enterobacteriaceae and associated microbial resistance | Occupational exposure | Enterobacteriaceae | Statistics: Risk |
| Frana | (192) | Isolation and characterization of methicillin-resistant *Staphylococcus aureus* from pork farms and visiting veterinary students | Occupational exposure | *S. aureus* | Statistics: Risk |
| Wulf | (193) | Prevalence of methicillin-resistant *Staphylococcus aureus* among veterinarians: an international study. | Occupational exposure | *S. aureus* | Statistics: OR |
| Fang | (194) | Livestock-associated methicillin-resistant *Staphylococcus aureus* ST9 in pigs and related personnel in Taiwan. | Occupational exposure | *S. aureus* | Statistics: Risk, genes |
| Khanna | (195) | Methicillin resistant *Staphylococcus aureus* colonization in pigs and pig farmers. | Occupational exposure | *S. aureus* | Statistics: Risk |
| Witte | (196) | Emergence of caMRSA infections in humans caused by MRSA CC398 which has its main reservoir in animals | Occupational exposure | *S. aureus* | Statistics: Risk |
| Cuny | (15) | Methicillin-resistant *Staphylococcus aureus* from infections in horses in Germany are frequent colonizers of veterinarians but rare among MRSA from infections in humans | Occupational exposure, pet to human | *S. aureus* | Statistics: Risk and genes |
| Drougka | (197) | Interspecies spread of *Staphylococcus aureus* clones among companion animals and human close contacts in a veterinary teaching hospital. Across-sectional study in Greece | Occupational Exposure, pet to human | *S. aureus* | Statistics: Risk |
| Graells | (125) | Livestock veterinarians at high risk of acquiring methicillin-resistant *Staphylococcus aureus* ST398. | Occupational Exposure | *S. aureus* | Statistics: Risk |
| Oppliger | (198) | Antimicrobial resistance of *Staphylococcus aureus* strains acquired by pig farmers from pigs | Occupational Exposure | *S. aureus* | Statistics: Risk |
| Nakane | (199) | Long-term colonization by blaCTX-M-harboring *Escherichia coli* in healthy Japanese people engaged in food handling | Occupational Exposure | *E. coli* | Statistics: Risk |
| Castro | (200) | Food handlers as potential sources of dissemination of virulent strains of *Staphylococcus aureus* in the community. | Occupational Exposure | *S. aureus* | Statistics: Risk |
| Zurfluh | (201) | Screening for fecal carriage of MCR-producing Enterobacteriaceae in healthy humans and primary care patients | Occupational Exposure | Enterobacteriaceae | Statistics: Risk |
| Cuny | (202) | Occurrence of cfr-mediated multiresistance in staphylococci from veal calves and pigs, from humans at the corresponding farms, and from veterinarians and their family members | Occupational Exposure | Staphylococci | Statistics: Risk |
| Gràinne | (203) | The emergence and spread of multiple livestock-associated clonal complex 398 methicillin-resistant and methicillin-susceptible *Staphylococcus aureus* strains among animals and humans in the Republic of Ireland, 2010–2014 | Occupational Exposure, Pet to human | *S. aureus* | Genes |
| Knetsch | (11) | Whole genome sequencing reveals potential spread of Clostridium difficile between humans and farm animals in the Netherlands, 2002 to 2011. | Occupational Exposure | *S. aureus* | Genes |
| Lozano | (204) | Dynamic of nasal colonization by methicillin-resistant Staphylococcus aureus ST398 and ST1after mupirocin treatment in a family in close contact with pigs. | Occupational Exposure | *S. aureus* | Genes |
| Tzavaras | (205) | Diversity of vanA-type vancomycin-resistant Enterococcus faecium isolated from broilers, poultry slaughterers and hospitalized humans in Greece. | Occupational Exposure | Enterococcus faecium | Genes |
| Köck | (206) | Livestock-associated methicillin-resistant Staphylococcus aureus (MRSA) as causes of human infection and colonization in Germany | Occupational Exposure | *S. aureus* | Genes |
| Wang | (207) | Distribution of the multidrug resistance gene cfr in Staphylococcus isolates from pigs, workers, and the environment of a hog market and a slaughterhouse in Guangzhou, China | Occupational Exposure | Staphylococci | Genes |
| Mularoni | (208) | Outcome of Transplantation Using Organs From Donors Infected or Colonized With Carbapenem-Resistant Gram-Negative Bacteria. | Organ | Enterobacteriaceae | Statistics: Risk |
| Goldberg | (209) | Organ transplantation from a donor colonized with a multidrug-resistant organism: A case report | Organ | Other | Statistics: Risk |
| Mingquan | (210) | Distinct mechanisms of acquisition of mcr-1 –bearing plasmid by Salmonella strains recovered from animals and food samples | Other (animal to meat of animal) | Salmonella | Genes |
| Mork | (211) | Comprehensive modeling reveals proximity, seasonality, and hygiene practices as keydeterminants of MRSA colonization in exposed households. | Other Bathing/Showering, space sharing, other Soap | *S. aureus* | Statistics: OR |
| Gandolfi-Decristophoris | (212) | Evaluation of pet contact as a risk factor for carriage of multidrug-resistant staphylococci in nursing home residents | Pet to human | Staphylococci | Statistics: OR |
| Morita | (213) | Survey of Methicillin–Resistant *Staphylococcus aureus* (MRSA) Carriage in Healthy College Students, Hawaii | Pet to human, water(exposure)to human | *S. aureus* | Statistics: Risk |
| De Martino | (9) | Methicillin-resistant staphylococci isolated from healthy horses and horse personnel in Italy. | Pet to human | Staphylococci | Statistics: Risk and genes |
| Loncaric | (214) | Suspected goat-to-human transmission of methicillin-resistant *Staphylococcus aureus* sequence type 398 | Pet to human | *S. aureus* | Statistics: Risk |
| Morris | (215) | Potential for pet animals to harbour methicillin-resistant *Staphylococcus aureus* when residing with human MRSA patients | Pet to human | *S. aureus* | Statistics: Risk and genes |
| Boost | (216) | Prevalence of Staphylococcus aureus carriage among dogs and their owners | Pet to human | *S. aureus* | Genes^rs^ |
| Boehmer | (217) | Phenotypic characterization and whole genome analysis of extended-spectrum beta-lactamase-producing bacteria isolated from dogs in Germany | Pet to human | *E. coli* | Genes |
| Damborg | (218) | Dogs are a reservoir of ampicillin-resistant Enterococcus faecium lineages associated with human infections | Pet to human | Enterococcus faecium | Genes |
| Gordoncillo | (10) | Detection of methicillin-resistant *Staphylococcus aureus* (MRSA) in backyard pigs and their owners, Michigan, USA. | Pet to human | *S. aureus* | Genes |
| van den Eede | (219) | MRSA carriage in the equine community: an investigation of horse-caretaker couples | Pet to human | *S. aureus* | Genes |
| Walther | (220) | Sharing more than friendship - nasal colonization with coagulase-positive staphylococci (CPS) and co-habitation aspects of dogs and their owners | Pet to human | *Staphylococcus pseudintermedius* | Genes^rs^ |
| Shorman | (221) | Risk factors associated with vancomycin-resistant enterococcus in intensive care unit settings in Saudi Arabia | Prior colonised patient in room | Other | Statistics: OR |
| Nseir | (222) | Risk of acquiring multidrug-resistant Gram-negative bacilli from prior room occupants in the intensive care unit | Prior colonised patient in room | *Pseudomonas aeruginosa*, *Acinetobacter baumannii* | Statistics: OR |
| Morales Barroso | (223) | Intestinal colonization due to *Escherichia coli* ST131: Risk factors and prevalence | Space sharing | *E. coli* | Statistics: OR |
| Torres-Gonzalez | (224) | Factors associated to prevalence and incidence of carbapenem-resistant Enterobacteriaceae fecal carriage: A cohort study in a Mexican tertiary care hospital | Space sharing | Enterobacteriaceae | Statistics: OR |
| Ben-David | (225) | Carbapenem-resistant *Klebsiella pneumoniae* in post-acute-care facilities in Israel | Space sharing | Enterobacteriaceae | Statistics: OR |
| Papadimitriou-Olivgeris | (226) | Risk factors for enterococcal infection and colonization by vancomycin-resistant enterococci in critically ill patients | Space sharing | VRE | Statistics: OR |
| Jiménez-Truque | (227) | Association Between Contact Sports and Colonization with Staphylococcus aureus in a Prospective Cohort of Collegiate Athletes | Space sharing | *S. aureus* | Statistics: OR |
| Fossi Djembi | (228) | Factors associated with Vancomycin-resistant Enterococcus acquisition during a large outbreak | Space sharing | VRE | Statistics: PR |
| Ulstad | (229) | Carriage of ESBL/AmpC-producing or ciprofloxacin non-susceptible *Escherichia coli* and *Klebsiella spp*. in healthy people in Norway | Travelling - | Enterobacteriaceae | Statistics: OR |
| Vading | (230) | Frequent acquisition of low-virulence strains of ESBL-producing *Escherichia coli* in travellers | Travelling + | Enterobacteriaceae | Statistics: OR |
| Peirano | (231) | Rates of colonization with extended-spectrum β-lactamase-producing Escherichia coli in Canadian travellers returning from South Asia: a cross-sectional assessment | Travelling +, travelling (eating food) + | *E. coli* | Statistics: OR |
| MacFadden | (232) | A passage from India: Association between air traffic and reported cases of New Delhi Metallo-beta-lactamase 1 from 2007 to 2012 | Travelling - | Enterobacteriaceae | Statistics: OR |
| Epelboin | (233) | High rate of multidrug-resistant gram-negative bacilli carriage and infection in hospitalized returning travelers: A cross-sectional cohort study | Travelling + | *E. coli* | Statistics: OR |
| Lorme | (234) | Acquisition of plasmid-mediated cephalosporinase producing Enterobacteriaceae after a travel to the tropics | Travelling + | Enterobacteriaceae | Statistics: OR, risk |
| Nurjadi | (235) | Skin and soft tissue infections in intercontinental travellers and the import of multi-resistant Staphylococcus aureus to Europe | Travelling - | *S. aureus* | Statistics: OR |
| Ostholm-Balkhed | (236) | Travel-associated faecal colonization with ESBL-producing Enterobacteriaceae: incidence and risk factors | Travelling + | Enterobacteriaceae | Statistics: OR |
| Yaita | (237) | Epidemiology of extended-spectrum β-lactamase producing *Escherichia coli* in the stools of returning Japanese travelers, and the risk factors for colonization | Travelling + | *E. coli* | Statistics: OR |
| Sannes | (238) | Predictors of antimicrobial-resistant *Escherichia coli* in the feces of vegetarians and newly hospitalized adults in Minnesota and Wisconsin | Travelling - | *E. coli* | Statistics: PR |
| Islam | (239) | Intestinal carriage of third-generation cephalosporin-resistant and extended-spectrum β-lactamase-producing Enterobacteriaceae in healthy US children | Travelling - | Enterobacteriaceae | Statistics: OR |
| Coleman | (240) | The role of drinking water in the transmission of antimicrobial-resistant *E. coli* | Travelling -, drinking water to human, eating meat to human | *E. coli* | Statistics: PR |
| Tängdén | (241) | Foreign travel is a major risk factor for colonization with Escherichia coli producing CTX-M-type extended-spectrum beta-lactamases: a prospective study with Swedish volunteers | Travelling + | Enterobacteriaceae | Statistics: Risk |
| Pires | (242) | Polyclonal intestinal colonization with Extended-Spectrum Cephalosporin-Resistant Enterobacteriaceae upon traveling to India | Travelling + | Enterobacteriaceae | Statistics: Risk |
| Lausch | (243) | Colonisation with multi-resistant Enterobacteriaceae in hospitalised Danish patients with a history of recent travel: a cross-sectional study | Travelling - | *E. coli* | Statistics: Risk |
| Arcilla | (244) | Import and spread of extended-spectrum β-lactamase-producing Enterobacteriaceae by international travellers (COMBAT study): a prospective, multicentre cohort study | Travelling + | Enterobacteriaceae | Statistics: Risk |
| Blyth | (245) | Antimicrobial resistance acquisition after international travel in U.S. travelers | Travelling + | *E. coli* | Statistics: Risk |
| Bevan | (246) | Acquisition and loss of CTX-M-producing and non-producing *Escherichia coli* in the fecal microbiome of travelers to South Asia | Travelling + | *E. coli* | Statistics: Risk |
| Lübbert | (247) | Colonization with extended-spectrum beta-lactamase-producing and carbapenemase-producing Enterobacteriaceae in international travelers returning to Germany | Travelling + | Enterobacteriaceae | Statistics: Risk |
| Nakayama | (248) | Carriage of colistin-resistant, extended-spectrum β-lactamase-producing Escherichia coli harboring the mcr-1 resistance gene after short-term international travel to Vietnam | Travelling + | *E. coli* | Statistics: Risk |
| Valverde | (249) | Intestinal Colonisation with multidrug-resistant Enterobacteriaceae in travellers, immigrants and 'visiting friends and relatives': Dominance of E. coli producing CTX-M enzymes | Travelling - | Enterobacteriaceae | Statistics: Risk |
| Johnning | (250) | Quinolone resistance mutations in the faecal microbiota of Swedish travellers to India | Travelling + | *E. coli* | Statistics: Risk difference |
| Sun | (251) | Varying High Levels of faecal carriage of Extended-Spectrum Beta-Lactamase producing Enterobacteriaceae in rural villages in Shandong, China: Implications for global health | Vegetables to human, drinking water to human, pet to human, nearby farm to human | *E. coli* | Statistics: OR |
| O'Flaherty | (252) | Human exposure to antibiotic resistant-*Escherichia coli* through irrigated lettuce. | Vegetables to human | *E. coli* | Bacteria intake |
| Dekić | (253) | Emerging human pathogen *Acinetobacter baumannii* in the natural aquatic environment: a public health risk? | Water to Animal | *Acinetobacter baumannii* | Bacteria intake |
| Goldstein | (254) | Higher prevalence of coagulase-negative staphylococci carriage among reclaimed water spray irrigators. | Water(exposure)to human | Staphylococci | Statistics: OR |
| Leonard | (255) | Exposure to and colonisation by antibiotic-resistant *E. coli* in UK coastal water users: Environmental surveillance, exposure assessment, and epidemiological study (Beach Bum Survey). | Water(exposure)to human | *E. coli* | Statistics: OR |
| Yang | (256) | Discharge of KPC-2 genes from the WWTPs contributed to their enriched abundance in the receiving river | Water(exposure)to human | Enterobacteriaceae | Genes |

**Additional file 1.**

This .docx file contains supporting information of this study, namely S1 Appendix containing the search terms, S2 Appendix containing the Prisma Checklist, S1 Table containing the frequencies of each transmission route identified, S2 Table containing the frequencies of bacteria studied for transmission routes included in quantitative synthesis and S3 Table containing the list of studies of which estimates were included in the synthesis.

**Additional file 2.**Data as .xlsx file called “Additional file 2. Transmission_Review_Data_180820”.

**Additional file 3.**Script for analyses and meta-analyses as .R file called “Additional file 3. Script Systematic Review Godijk et al”.
**Additional file 4.**Script for forest plots as .R file called “Additional file 4. Forest plots Systematic Review Godijk et al”.
