# Supplementary material for: Transmission routes of antibiotic resistant bacteria: a systematic review
Source: BMC Infect Dis. 2022 May 20;22:482. doi: 10.1186/s12879-022-07360-z (PMC9123679; doi:10.1186/s12879-022-07360-z)
Supplement: Supplementary file 3 — Additional file 3. R Script of the systematic review by Godijk et al. [file 12879_2022_7360_MOESM3_ESM.pdf]

Additional file 3. R Script of the systematic review by Godijk et al.

```
# Before running the script, make sure that all the packages are installed

# Also check the pathways for the data storage and data retrieval


#rm(list=ls())

# Clear any data left in your R, uncomment code above if you want to do so

library(foreign)

library(tidyverse)

library(rmeta)

library(msm)

library(lme4)

library(rJava)

library(xlsx)

library(gmodels)


############################################################ Meta-analysis
####################################

#install.packages("devtools")

#library(devtools)

#install.packages("digest")

#install.packages("curl")

#devtools::install_github("MathiasHarrer/dmetar")

#install.packages("dmetar")

#install.packages("remotes")

#library("remotes")

#install_github("MathiasHarrer/dmetar")


#backports

#library(dmetar)


# We will use a random effects meta analysis since not all study populations come from the same
underlying population

# By using this method, we account for more variance than when drawn from a single population
```

```
# There is another source of variance due to the fact that not all stem from the same underlying population

# We assume a distribution of effect sizes instead of 1 effect size

# we estimate the mean distribution of true effect sizes

# So next to some sampling error there is another error.

# The REM pays more attention to small studies when pooling the overall estimate


#DerSimonian-Laird estimator or Maximum Likelihood als tau


library(meta)

library(metafor)


# Load the final data file

Transmission <- read.xlsx("PATH TO YOUR COMPUTER/Transmission_Review_Data_180820.xlsx",1)

# Replace PATH TO YOUR COMPUTER to were you want to save the excel file

Transmission <- Transmission[,c(3:37)]

Transmission$Num_Estimate<-as.numeric(as.character(paste(Transmission$Num_Estimate)))

Transmission$Num_ub<-as.numeric(as.character(paste(Transmission$Num_ub)))

Transmission$Num_lb<-as.numeric(as.character(paste(Transmission$Num_lb)))

Transmission$Num_SE<-as.numeric(as.character(paste(Transmission$Num_SE)))


#Which bacteria

Table_Bacteria_grouped<-table(Transmission$GroupGood)

Table_Bacteria_grouped

pie(Table_Bacteria_grouped,main = "Organisms studied",r=1)

rm(Table_Bacteria_grouped)


#write.xlsx(Table_Bacteria_grouped, "PATH TO YOUR COMPUTER/Bacteria.xlsx")

# Replace PATH TO YOUR COMPUTER to were you want to save the excel file


test<-function (bacteriagroup) {
```

```
  if ("E. faecium and L. acidophilus " %in% bacteriagroup) {
    stop("Error: Recoding bacteria groups went wrong")
  }
}
test(Transmission$GroupGood) #We are checking if the recoding of the bacteria group went correct



# Transmission routes
Table_TransmissionRouteGroup <-table(Transmission$Transmission.Group)
Table_TransmissionRouteGroup

attach(Transmission)
# Number of methods
Methods<-table(Transmission$MethodGroup)
Methods

#Countries
Countries<-table(Transmission$Country)
Countries
barplot(Countries)
#write.xlsx(Countries, "PATH TO YOUR COMPUTER/Countries.xlsx")
# Replace PATH TO YOUR COMPUTER to were you want to save the excel file

#Subset methods
Modelling<-subset(Transmission, MethodGroup=="Modelling")
Genes<-subset(Transmission, Method.of.estimation=="Genes")
Stats<-subset(Transmission, MethodGroup=="Statistics", select = c(Method.of.estimation))
BacteriaIntake<-subset(Transmission, MethodGroup=="BacteriaIntake")

OR<-subset(Stats, Method.of.estimation=="OR", select = c(Method.of.estimation))
Risk<-subset(Stats, Method.of.estimation=="Risk", select = c(Method.of.estimation))
```

```
PR<-subset(Stats, Method.of.estimation=="PR", select = c(Method.of.estimation))

TR<-subset(Stats, Method.of.estimation=="Transmission rate", select = c(Method.of.estimation))

R0<-subset(Modelling, Method.of.estimation=='R0')

TransmisionRate<-subset(Modelling, Method.of.estimation=='transmission rate')

ImportanceRoute<-subset(Modelling, Method.of.estimation=='Importance of Route')

Casesperday<-subset(Modelling, Method.of.estimation=='cases per day')

Incidence<-subset(Modelling, Method.of.estimation=='Incidence')

Incidence_rate_ratio<-subset(Modelling, Method.of.estimation=='Incidence rate ratios ')

Acquisition<-subset(Modelling, Method.of.estimation=='acquisition rate')

TRratio<-subset(Modelling, Method.of.estimation=='acquisition rate')


#Methods of statistics

names_stats<-c("OR", "Risk", "PR", "TR")

count<-c(paste(count(OR)), paste(count(Risk)), paste(count(PR)),paste(count(TR)))

count

Table_Statistical_methods<-data.frame(names_stats, count)

Table_Statistical_methods



#Transmission routes of statistics

Table_TransmissionRouteGroupS <-subset(Transmission,
Transmission$Method.of.estimation=="OR")

Table_TransmissionRouteGroupS<- table(Table_TransmissionRouteGroupS$Transmission.Group)

Table_TransmissionRouteGroupS

Table_TransmissionRouteGroupS <-subset(Transmission,
Transmission$Method.of.estimation=="PR")

Table_TransmissionRouteGroupS<- table(Table_TransmissionRouteGroupS$Transmission.Group)

Table_TransmissionRouteGroupS

Table_TransmissionRouteGroupS <-subset(Transmission,
Transmission$Method.of.estimation=="Risk")

Table_TransmissionRouteGroupS<- table(Table_TransmissionRouteGroupS$Transmission.Group)

Table_TransmissionRouteGroupS
```

```
Table_TransmissionRouteGroupS <-subset(Transmission, Transmission$Method.of.estimation=="RR")

Table_TransmissionRouteGroupS<- table(Table_TransmissionRouteGroupS$Transmission.Group)

Table_TransmissionRouteGroupS


rm(TR,OR,PR, Risk, count,names_stats, Table_TransmissionRouteGroupS)


#Transmission routes of genes

Table_TransmissionRouteGroupG <-subset(Transmission, Transmission$Method.of.estimation=="Genes")

Table_TransmissionRouteGroupG<- table(Table_TransmissionRouteGroupG$Transmission.Group)

Table_TransmissionRouteGroupG

rm(Table_TransmissionRouteGroupG)


#Methods of Modelling

names_modelling<-c("R0", "Atttributable%", "Transmission rate", "cases per day", "Incidence", "Incidence rate ratio", "Acquisition rate")

count<-c(paste(count(R0)),paste(count(ImportanceRoute)), paste(count(TransmisionRate)), paste(count(Casesperday)), paste(count(Incidence)), paste(count(Incidence_rate_ratio)), paste(count(Acquisition)))

count

Table_Modelling_methods<-data.frame(names_modelling, count)

Table_Modelling_methods#One other is transmission rate (week), this I have to count as well as transmission rate and devide by seven + make a note that I did this

Modelling$Method.of.estimation

levels(Modelling$Method.of.estimation) # Those who have a value labelled "missing" we decided not to report


#Transmission routes of modelling

Table_TransmissionRouteGroupM <-subset(Transmission, Transmission$Method.of.estimation=="R0")

Table_TransmissionRouteGroupM<- table(Table_TransmissionRouteGroupM$Transmission.Group)

Table_TransmissionRouteGroupM
```

```
Table_TransmissionRouteGroupM <-subset(Transmission, Transmission$Method.of.estimation=="Importance of Route")

Table_TransmissionRouteGroupM<- table(Table_TransmissionRouteGroupM$Transmission.Group)

Table_TransmissionRouteGroupM

Table_TransmissionRouteGroupM <-subset(Transmission, Transmission$Method.of.estimation=="cases per day")

Table_TransmissionRouteGroupM<- table(Table_TransmissionRouteGroupM$Transmission.Group)

Table_TransmissionRouteGroupM

Table_TransmissionRouteGroupM <-subset(Transmission, Transmission$Method.of.estimation=="Incidence rate ratios ")

Table_TransmissionRouteGroupM<- table(Table_TransmissionRouteGroupM$Transmission.Group)

Table_TransmissionRouteGroupM

rm(Table_TransmissionRouteGroupM)


rm(count,names_modelling, TransmisionRate, ImportanceRoute, R0)


#Countries

Transmission$Country<-as.factor(Transmission$Country)

Table_Country<-table(Transmission$Country)

Table_Country

pie(Table_Country)


######################################################### Analysis which pathogens in the groups for meta-analysis ####

#First make subsets for eacht route that we do a meta-analysis for

Occupational_Exposure<-subset(Transmission, Transmission$Transmission.Group=="Occupational Exposure")

Family_member_occupational_exposure<- subset(Transmission, Transmission$Transmission.Group=="Family member occupational exposure")

Animal_to_air<-subset(Transmission, Transmission$Transmission.Group=="Animal-> Air")

Animal_to_environment<-subset(Transmission, Transmission$Transmission.Group=="Animal -> environment")

Animal_contact<-subset(Transmission, Transmission$Transmission.Group=="Animal Contact")
```

```
Breast_feeding<-subset(Transmission, Transmission$Transmission.Group=="Breast feeding")

Contact_with_infected<-subset(Transmission, Transmission$Transmission.Group=="Contact with infected person")

Eating_meat<-subset(Transmission, Transmission$Transmission.Group=="Eating Meat -> human")

Family_member_colonised<-subset(Transmission, Transmission$Transmission.Group=="Family member colonised")

Human_to_nearbyenv<-subset(Transmission, Transmission$Transmission.Group=="Human-> nearby environment")

Livestock_to_drinking_water<-subset(Transmission, Transmission$Transmission.Group=="Livestock-> drinking water")

Mother_to_child<-subset(Transmission, Transmission$Transmission.Group=="Mother to child")

Pet_to_human<-subset(Transmission, Transmission$Transmission.Group=="Pet-> human")

Prior_col_patient<-subset(Transmission, Transmission$Transmission.Group=="Prior colonised patient in room")

Space_sharing<-subset(Transmission, Transmission$Transmission.Group=="Space sharing")

Travelling<-subset(Transmission, Transmission$Transmission.Group=="Travelling")

Contaminated_room<-subset(Transmission, Transmission$Transmission.Group=="Contaminated room")

Sharing_water_source_with_animals<-subset(Transmission, Transmission$Transmission.Group=="Sharing water source with animals")

Non_commercial_animal_keeping<-subset(Transmission, Transmission$Transmission.Group=="Non commercial animal keeping")

Animal_to_animal<-subset(Transmission, Transmission$Transmission.Group=="Animal -> animal")

levels(Transmission$Transmission.Group)

Nearby_env_tohuman<-subset(Transmission, Transmission$Transmission.Group=="Nearby environment -> human")


#Per route, calculate the bacteria

Bacteria_Occupational<-table(Occupational_Exposure$GroupGood)

Bacteria_Occupational

Bacteria_Fam_Occu_Exp<-table(Family_member_occupational_exposure$GroupGood)

Bacteria_Fam_Occu_Exp

Bacteria_Animal_to_Air<-table(Animal_to_air$GroupGood)

Bacteria_Animal_to_Air
```

```
Bacteria_Animal_to_environment<-table(Animal_to_environment$GroupGood)

Bacteria_Animal_to_environment

Bacteria_Sharing_water_source_with_animals<-
table(Sharing_water_source_with_animals$GroupGood)

Bacteria_Sharing_water_source_with_animals

Bacteria_Non_commercial_animal_keeping<-table(Non_commercial_animal_keeping$GroupGood)

Bacteria_Non_commercial_animal_keeping

Bacteria_Breast_feeding<-table(Breast_feeding$GroupGood)

Bacteria_Breast_feeding

Bacteria_Contact_with_infected<-table(Contact_with_infected$GroupGood)

Bacteria_Contact_with_infected

Bacteria_Eating_meat<-table(Eating_meat$GroupGood)

Bacteria_Eating_meat

Bacteria_Family_mem_colonised<-table(Family_member_colonised$GroupGood)

Bacteria_Family_mem_colonised

Bacteria_human_to_nearbyenv<-table(Human_to_nearbyenv$GroupGood)

Bacteria_human_to_nearbyenv

Bacteria_livestock_to_drinkw<-table(Livestock_to_drinking_water$GroupGood)

Bacteria_livestock_to_drinkw

Bacteria_Mother_to_child<-table(Mother_to_child$GroupGood)

Bacteria_Mother_to_child

Bacteria_Pet_to_human<-table(Pet_to_human$GroupGood)

Bacteria_Pet_to_human

Bacteria_prior_col_patient<-table(Prior_col_patient$GroupGood)

Bacteria_prior_col_patient

Bacteria_Travelling<-table(Travelling$GroupGood)

Bacteria_Travelling

Bacteria_space_sharing<-table(Space_sharing$GroupGood)

Bacteria_space_sharing

Bacteria_Contaminated_room<-table(Contaminated_room$GroupGood)

Bacteria_Contaminated_room
```

```
######################################################## Calculate per route, per pathogen which methods have been used ####

CrossTable(Contaminated_room$GroupGood, Contaminated_room$Method.of.estimation)

CrossTable(Occupational_Exposure$GroupGood, Occupational_Exposure$Method.of.estimation)

CrossTable(Animal_to_air$GroupGood, Animal_to_air$Method.of.estimation)

CrossTable(Animal_to_environment$GroupGood, Animal_to_environment$Method.of.estimation)

Table<-table(subset(Sharing_water_source_with_animals, Sharing_water_source_with_animals$GroupGood=="E.Coli")$Method.of.estimation)

Table

CrossTable(Non_commercial_animal_keeping$GroupGood, Non_commercial_animal_keeping$Method.of.estimation)

CrossTable(Breast_feeding$GroupGood, Breast_feeding$Method.of.estimation)

CrossTable(Contact_with_infected$GroupGood, Contact_with_infected$Method.of.estimation)

Table1<-table(subset(Eating_meat, Eating_meat$GroupGood=="E.Coli")$Method.of.estimation)

Table1

CrossTable(Family_member_colonised$GroupGood, Family_member_colonised$Method.of.estimation)

CrossTable(Family_member_occupational_exposure$GroupGood, Family_member_occupational_exposure$Method.of.estimation)

CrossTable(Human_to_nearbyenv$GroupGood, Human_to_nearbyenv$Method.of.estimation)

CrossTable(Mother_to_child$GroupGood, Mother_to_child$Method.of.estimation)

CrossTable(Pet_to_human$GroupGood, Pet_to_human$Method.of.estimation)

CrossTable(Prior_col_patient$GroupGood, Prior_col_patient$Method.of.estimation)

CrossTable

CrossTable(Animal_to_animal$GroupGood, Animal_to_animal$Method.of.estimation)

rm(Table, Table1)


########################################### Starting with OR########################

OR<-subset(Transmission, Method.of.estimation=="OR")

OR$Num_ub <-as.numeric(as.character(OR$ub))

OR$Num_Estimate <-as.numeric(as.character(OR$Estimate))

OR$ub[239]#"B 2.8632"
```

```
OR$Num_ub[239]<-2.8632 # estimates keeps appearing with a B in front in our dataset when we load in into R, therefore we replace it with just the value

OR$Num_ub[239]

OR$Estimate[239]

OR$Num_Estimate[239]<-2.04

OR$Estimate[239]


OR$logOR<-log(OR$Num_Estimate)

OR$loglb<-log(OR$Num_lb)

OR$logub<-log(OR$Num_ub)

OR$diflogCI<- OR$logub-OR$loglb

OR$logSE<-OR$diflogCI/3.92

OR$logSE[17]

OR$logSE[17]<-(1.88706965)/1.96

OR$logSE[17]

OR$logOR[17]<-log(6.60)

OR$logSE[16]

OR$logSE[16]<-(0.69314718--2.30258509)/1.96

OR$logSE[16]

OR$logSE[33]

OR$logSE[33]<-(3.09104245-0.530628251)/1.96

OR$logSE[33]

OR$logSE[34]

OR$logSE[34]<-(2.77258872-0.262364264)/1.96

OR$logSE[34]



#Creating subsets per transmission group for ORs

table((OR$Transmission.Group))


OR_Family_member_occu_exp<-subset(OR, Transmission.Group =="Family member occupational exposure")
```

```
OR_Occupational_Exp <-subset(OR, Transmission.Group=="Occupational Exposure")

OR_Livestock_to_drink_water<- subset(OR, Transmission.Group=="Livestock-> drinking water")

OR_Nearbyfarm_to_human <-subset(OR, Transmission.Group=="Nearby farm-> human")

OR_Eating_Meat<- subset(OR, Transmission.Group=="Eating Meat -> human")

OR_Waterdrink_to_human<-subset(OR, Transmission.Group=="Water (drinking) -> human")

OR_Pet_to_human<-subset(OR, Transmission.Group=="Pet-> human")

OR_Animal_contact<-subset(OR, Transmission.Group=="Animal Contact")

OR_Travelling<-subset(OR, Transmission.Group=="Travelling")

OR_Prior_col_roomoccu<-subset(OR, Transmission.Group=="Prior colonised patient in room")

OR_Mother_to_Child<- subset(OR, Transmission.Group=="Mother to child")

OR_Family_mem_colonised<-subset(OR, Transmission.Group=="Family member colonised")

OR_Contaminated_room<- subset(OR, Transmission.Group=="Contaminated room")

OR_Contact_inf_person<-subset(OR, Transmission.Group=="Contact with infected person")

OR_Space_sharing<-subset(OR, Transmission.Group=="Space sharing")

OR_Fomites<-subset(OR, Transmission.Group=="Fomites")


# Now for risk ratios

RR<-subset(Transmission, Method.of.estimation=="RR")


# I am making subsets for prevalence ratio's

PR<- subset(Transmission, Method.of.estimation=="PR")

PR$LogPR<-log(PR$Num_Estimate)


# We also need to back calculate the SE for all the estimates

# First we check our code with an example of which we know the answer, the answer should be
0.3474

(log(0.869331)-log(.44))/1.96 # the formula is correct

PR$SE_logPR<-(log(PR$Num_ub)-log(PR$Num_Estimate))/1.96

head(PR$Num_Estimate)

PR_alles<-metagen(log(PR$Num_Estimate), lower = log(PR$Num_lb), upper = log(PR$Num_ub),
studlab = paste(PR$Author), sm="RR")

PR_alles
```

```
levels(PR$Transmission.Group)


PR_Fam_Col<-subset(PR, PR$Transmission.Group=="Family member colonised")

PR_Occupation_Exp<-subset(PR, PR$Transmission.Group=="Occupational Exposure")

PR_Fam_Occu_Exp<-subset(PR, PR$Transmission.Group=="Family member occupational exposure")

PR_Pet_Human<-subset(PR, PR$Transmission.Group=="Pet-> human")

PR_SpaceShare<-subset(PR, PR$Transmission.Group=="Space sharing")

PR_Travel<-subset(PR, PR$Transmission.Group=="Travelling")


# I am making subsets for risks

# I created subsets for all possible levels of Transmission groups for risk

Risk<-subset(Transmission, Method.of.estimation=="Risk")



Risk$Sample.size<-as.numeric(as.character(Risk$Sample.size)) # Make sample size numeric

Risk$Num_Estimate<- as.numeric(gsub("[\\%,]","", Risk$Estimate)) #Make the percentage numeric and remove the % sign

Risk$Num_Estimate<- Risk$Num_Estimate/100 # We turn this into proportion instead of percentage

Risk$Num_Estimate<-abs(Risk$Num_Estimate)


Risk$Num_Estimate[176]

Risk$Num_Estimate[176]<-0.884

Risk$Num_Estimate[176]

Risk$SE<- sqrt(Risk$Num_Estimate*(1-Risk$Num_Estimate)/Risk$Sample.size) # This is the normal SE

# Before I can do a meta analysis for proportions, I have to give eacht study an ID (this cannot be author as some authors have multiple estimates)


Risk$id<-c(1:242)

Risk$id


#Now I have to get 1) sample size 2) number of events 3) sample size- number of events
```

```
#I need this for GLMER to get a logit estimate

Risk$Events<-Risk$Sample.size*Risk$Num_Estimate

#Risk<-Risk[,c(1:5, 7:36,6)]

Risk$Events_integer<-round(Risk$Events)

Risk$No_Event<-Risk$Sample.size-Risk$Events_integer



Risk_Occupational_Exposure<-subset(Risk,  Risk$Transmission.Group=="Occupational Exposure")

Risk_Fam_mem_occu_exposure<-subset(Risk,  Risk$Transmission.Group=="Family member
occupational exposure")

Risk_Travelling<-subset(Risk,  Risk$Transmission.Group=="Travelling")

Risk_hum_to_nearenvironment<-subset(Risk,  Risk$Transmission.Group=="Human-> nearby
environment")

Risk_Cont_Inf_Person<-subset(Risk,  Risk$Transmission.Group=="Contact with infected person")

Risk_Animal_to_Air<-subset(Risk,  Risk$Transmission.Group=="Animal-> Air")

Risk_Animal_to_water<-subset(Risk,  Risk$Transmission.Group=="Animal-> Water")

Risk_Animal_to_environment<-subset(Risk,  Risk$Transmission.Group=="Animal -> environment")

Risk_Household_Col<-subset(Risk,  Risk$Transmission.Group=="Household member colonised")

Risk_Mother_to_child<-subset(Risk,  Risk$Transmission.Group=="Mother to child")

Risk_Water_drink_Human<-subset(Risk,  Risk$Transmission.Group=="Water (drinking) -> human")

Risk_Organ<-subset(Risk,  Risk$Transmission.Group=="Organ")

Risk_Fomites<-subset(Risk,  Risk$Transmission.Group=="Fomites")

Risk_Pet_to_human<-subset(Risk,  Risk$Transmission.Group=="Pet-> human")

Risk_Water_exposure_to_human<-subset(Risk,  Risk$Transmission.Group=="Water(exposure)->
human")

Risk_Food_to_animal<-subset(Risk,  Risk$Transmission.Group=="Food-> animal")

Risk_Intervention<-subset(Risk,  Risk$Transmission.Group=="Intervention")

Risk_Human_to_Air	<-subset(Risk,  Risk$Transmission.Group=="Human-> Air")

Risk_Animal_to_Animal<-subset(Risk,  Risk$Transmission.Group=="Animal -> animal")

Risk_Motheranimal_to_animalchild<-subset(Risk,  Risk$Transmission.Group=="Mother (animal) ->
child")
```

```
############################################################## Meta analysis per pathogen per route ###############################

############################################################## Animal to air

Animal_to_air_Ecoli_Risk<- subset(Risk_Animal_to_Air, Risk_Animal_to_Air$GroupGood=="E.Coli")

poultry_to_air_Ecoli_Risk<-Animal_to_air_Ecoli_Risk[c(2,3),]

pig_to_air_Ecoli_Risk<-Animal_to_air_Ecoli_Risk[c(1,4),]


poultry_to_air_Ecoli_Risk$id<-c(1,2)

poultry_to_air_Ecoli_Risk$Num_Estimate<- (as.numeric(gsub("[\\%,]","", poultry_to_air_Ecoli_Risk$Estimate)))/100

poultry_to_air_Ecoli_Risk$Sample.size<- as.numeric(as.character(poultry_to_air_Ecoli_Risk$Sample.size))

poultry_to_air_Ecoli_Risk$Events<- poultry_to_air_Ecoli_Risk$Sample.size*poultry_to_air_Ecoli_Risk$Num_Estimate

poultry_to_air_Ecoli_Risk$Events_integer<-round(poultry_to_air_Ecoli_Risk$Events)

poultry_to_air_Ecoli_Risk$No_Event<-poultry_to_air_Ecoli_Risk$Sample.size- poultry_to_air_Ecoli_Risk$Events_integer


#Dit klopt niet

model.AtoA_EcoliRisk_poultry<- glmer(Events_integer/Sample.size~1+(1|id),weights=Sample.size,data=poultry_to_air_Ecoli_Risk,family="binomial")

summary(model.AtoA_EcoliRisk_poultry)

se.logit.AtoApoultry <- sqrt(vcov(model.AtoA_EcoliRisk_poultry))

model.AtoA_EcoliRisk_poultry@beta


pig_to_air_Ecoli_Risk$id<-c(1,2)

pig_to_air_Ecoli_Risk$Num_Estimate<- (as.numeric(gsub("[\\%,]","", pig_to_air_Ecoli_Risk$Estimate)))/100

pig_to_air_Ecoli_Risk$Sample.size<-as.numeric(as.character(pig_to_air_Ecoli_Risk$Sample.size))

pig_to_air_Ecoli_Risk$Events<- pig_to_air_Ecoli_Risk$Sample.size*pig_to_air_Ecoli_Risk$Num_Estimate

pig_to_air_Ecoli_Risk$Events_integer<-round(pig_to_air_Ecoli_Risk$Events)
```

```
pig_to_air_Ecoli_Risk$No_Event<-pig_to_air_Ecoli_Risk$Sample.size-
pig_to_air_Ecoli_Risk$Events_integer


model.pig_to_air_Ecoli_Risk<-
glmer(Events_integer/Sample.size~1+(1|id),weights=Sample.size,data=pig_to_air_Ecoli_Risk,family=
"binomial")

summary(model.pig_to_air_Ecoli_Risk)

se.logit.AtoApig <- sqrt(vcov(model.pig_to_air_Ecoli_Risk))

model.pig_to_air_Ecoli_Risk@beta



Animal_to_air_Sareus_Risk<- subset(Risk_Animal_to_Air, Risk_Animal_to_Air$GroupGood=="S.
Aureus")

Pig_to_air_Sareus_Risk<-Animal_to_air_Sareus_Risk[c(1,2),]

Cattle_to_air_Sareus_Risk<-Animal_to_air_Sareus_Risk[c(3:24),]


Pig_to_air_Sareus_Risk$id<-c(1,2)

Pig_to_air_Sareus_Risk$Num_Estimate<- (as.numeric(gsub("[\\%,]","",
Pig_to_air_Sareus_Risk$Estimate)))/100

Pig_to_air_Sareus_Risk$Sample.size<-as.numeric(as.character(Pig_to_air_Sareus_Risk$Sample.size))

Pig_to_air_Sareus_Risk$Events<-
Pig_to_air_Sareus_Risk$Sample.size*Pig_to_air_Sareus_Risk$Num_Estimate

Pig_to_air_Sareus_Risk$Events_integer<-round(Pig_to_air_Sareus_Risk$Events)

Pig_to_air_Sareus_Risk$No_Event<-Pig_to_air_Sareus_Risk$Sample.size-
Pig_to_air_Sareus_Risk$Events_integer


model.pig_to_air_Saureus_Risk<-
glmer(Events_integer/Sample.size~1+(1|id),weights=Sample.size,data=Pig_to_air_Sareus_Risk,famil
y="binomial")

se.logit.AtoApigAreus<- sqrt(vcov(model.pig_to_air_Saureus_Risk))


Cattle_to_air_Sareus_Risk$id<-c(1:22)

Cattle_to_air_Sareus_Risk$Num_Estimate<- (as.numeric(gsub("[\\%,]","",
Cattle_to_air_Sareus_Risk$Estimate)))/100
```

```
Cattle_to_air_Sareus_Risk$Sample.size<-
as.numeric(as.character(Cattle_to_air_Sareus_Risk$Sample.size))

Cattle_to_air_Sareus_Risk$Events<-
Cattle_to_air_Sareus_Risk$Sample.size*Cattle_to_air_Sareus_Risk$Num_Estimate

Cattle_to_air_Sareus_Risk$Events_integer<-round(Cattle_to_air_Sareus_Risk$Events)

Cattle_to_air_Sareus_Risk$No_Event<-Cattle_to_air_Sareus_Risk$Sample.size-
Cattle_to_air_Sareus_Risk$Events_integer


model.cattle.to.air.risk<-
glmer(Events_integer/Sample.size~1+(1|id),weights=Sample.size,data=Cattle_to_air_Sareus_Risk,fa
mily="binomial")

se.logit.AtoAcattle<-sqrt(vcov(model.cattle.to.air.risk))

############################################################ Animal to animal

# E. coli

chicken_to_chicken_EColiTR<- subset(Animal_to_animal, Animal_to_animal$GroupGood=="E.Coli")
# All are poultry

chicken_to_chicken_EColiTR<-
subset(chicken_to_chicken_EColiTR,chicken_to_chicken_EColiTR$Method.of.estimation=="transmis
sion rate")

#cannot be pooled due to study dissimlarities, its the same study but 1 has infected chicken and the
other not so they do not estimate the same thing


# S. Aureus

animal_to_animal_Saurues<-subset(Animal_to_animal, Animal_to_animal$GroupGood=="S.
Aureus")

pig_to_pig_Saureus<-subset(Animal_to_animal, Animal_to_animal$Animal.involved.in.route=="pig")

# could not be pooled, same study different time frame (just after release vs weeks after release)

cattle_to_cattle_Saureus<-subset(Animal_to_animal,
Animal_to_animal$Method.of.estimation=="transmission rate (week)")

# could not be pooled, same study different time frame (just after release vs weeks after release)


#R0

animal_to_animal_Saurues_r0<-subset(Animal_to_animal, Animal_to_animal$GroupGood=="S.
Aureus" & Animal_to_animal$Method.of.estimation=="R0")
```

```
animal_to_animal_Saurues_r0$logOR<-log(animal_to_animal_Saurues_r0$Num_Estimate)

animal_to_animal_Saurues_r0$loglb<-log(animal_to_animal_Saurues_r0$Num_lb)

animal_to_animal_Saurues_r0$logub<-log(animal_to_animal_Saurues_r0$Num_ub)

animal_to_animal_Saurues_r0$diflogCI<- animal_to_animal_Saurues_r0$logub-
animal_to_animal_Saurues_r0$loglb

animal_to_animal_Saurues_r0$logSE<-animal_to_animal_Saurues_r0$diflogCI/3.92


r0.animalanimal<-rma(yi = (animal_to_animal_Saurues_r0$logOR) , sei =
(animal_to_animal_Saurues_r0$logSE), method = "ML",measure = "OR")

summary(r0.animalanimal) #moet logor en log se nog toevoegen hier, helaas

?rma


#S. pseudointermedius

pet_to_pet<-subset(Animal_to_animal, Animal_to_animal$GroupGood=="Staphylococcus
pseudintermedius")

pet_to_pet_risk_speudi<-subset(pet_to_pet, pet_to_pet$Method.of.estimation=="Risk")


pet_to_pet_risk_speudi$id<-c(1,2)

pet_to_pet_risk_speudi$Num_Estimate<- (as.numeric(gsub("[\\%,]","",
pet_to_pet_risk_speudi$Estimate)))/100

pet_to_pet_risk_speudi$Sample.size<-
as.numeric(as.character(pet_to_pet_risk_speudi$Sample.size))

pet_to_pet_risk_speudi$Events<-
pet_to_pet_risk_speudi$Sample.size*pet_to_pet_risk_speudi$Num_Estimate

pet_to_pet_risk_speudi$Events_integer<-round(pet_to_pet_risk_speudi$Events)

pet_to_pet_risk_speudi$No_Event<-pet_to_pet_risk_speudi$Sample.size-
pet_to_pet_risk_speudi$Events_integer


model.pet_to_pet_speudo.risk<-
glmer(Events_integer/Sample.size~1+(1|id),weights=Sample.size,data=pet_to_pet_risk_speudi,famil
y="binomial")

summary(model.pet_to_pet_speudo.risk)

se.logit.pet.Spseudo<-sqrt(vcov(model.pet_to_pet_speudo.risk))
```

```
############################################################ Animal to environment

# E. coli


Animal_to_environment_Ecoli<-subset(Animal_to_environment,
Animal_to_environment$GroupGood=="E.Coli")

pig_to_env_ecoli<-subset(Animal_to_environment_Ecoli,
Animal_to_environment_Ecoli$Animal.involved.in.route=="pig")

poultry_to_env<-subset(Animal_to_environment_Ecoli,
Animal_to_environment_Ecoli$Animal.involved.in.route=="poultry")


pig_to_env_ecoli$id<-c(1,2)

pig_to_env_ecoli$Num_Estimate<- (as.numeric(gsub("[\\%,]","", pig_to_env_ecoli$Estimate)))/100

pig_to_env_ecoli$Sample.size<-as.numeric(as.character(pig_to_env_ecoli$Sample.size))

pig_to_env_ecoli$Events<-pig_to_env_ecoli$Sample.size*pig_to_env_ecoli$Num_Estimate

pig_to_env_ecoli$Events_integer<-round(pig_to_env_ecoli$Events)

pig_to_env_ecoli$No_Event<-pig_to_env_ecoli$Sample.size-pig_to_env_ecoli$Events_integer


model.pig_to_env<-
glmer(Events_integer/Sample.size~1+(1|id),weights=Sample.size,data=pig_to_env_ecoli,family="bin
omial")

se.logit.pig_env<-sqrt(vcov(model.pig_to_env))

se.logit.pig_env


poultry_to_env<-poultry_to_env[c(1,2),] #only first 2 are similar

poultry_to_env$id<-c(1,2)

poultry_to_env$Num_Estimate<- (as.numeric(gsub("[\\%,]","", poultry_to_env$Estimate)))/100

poultry_to_env$Sample.size<-as.numeric(as.character(poultry_to_env$Sample.size))

poultry_to_env$Events<-poultry_to_env$Sample.size*poultry_to_env$Num_Estimate

poultry_to_env$Events_integer<-round(poultry_to_env$Events)

poultry_to_env$No_Event<-poultry_to_env$Sample.size-poultry_to_env$Events_integer
```

```
model.poultry_to_env<-
glmer(Events_integer/Sample.size~1+(1|id),weights=Sample.size,data=poultry_to_env,family="bino
mial")

se.logit.poultry_env<-sqrt(vcov(model.poultry_to_env))


############################################################ Sharing water

# same study so have to check why there are different estimates

# not going to pool as it compares wild-life and lifestock sharing water with only livestock sharing


############################################################ non commercial animal
keeping

# Not pooled due to study asimilarities, all different kind of animals


############################################################ Breast feeding

# E. coli

BreastF_ecoli<-subset(Breast_feeding, Breast_feeding$GroupGood=="E.Coli")

BreastF_ecoli_OR<- subset(BreastF_ecoli, BreastF_ecoli$Method.of.estimation=="OR")


BreastF_ecoli_OR$logOR<-log(BreastF_ecoli_OR$Num_Estimate)

BreastF_ecoli_OR$loglb<-log(BreastF_ecoli_OR$Num_lb)

BreastF_ecoli_OR$logub<-log(BreastF_ecoli_OR$Num_ub)

BreastF_ecoli_OR$diflogCI<- BreastF_ecoli_OR$logub-BreastF_ecoli_OR$loglb

BreastF_ecoli_OR$logSE<-BreastF_ecoli_OR$diflogCI/3.92


or.breastf<-rma(yi = (BreastF_ecoli_OR$logOR) , sei = (BreastF_ecoli_OR$logSE), method =
"ML",measure = "OR")

summary(or.breastf) #moet logor en log se nog toevoegen hier, helaas


#enterobacteriae

BreastF_entero<-subset(Breast_feeding,
Breast_feeding$GroupGood=="Enterobacteriaceae(multiple or unspecified)")

BreastF_entero_OR<- subset(BreastF_entero, BreastF_entero$Method.of.estimation=="OR")
```

```
BreastF_entero_OR$logOR<-log(BreastF_entero_OR$Num_Estimate)

BreastF_entero_OR$loglb<-log(BreastF_entero_OR$Num_lb)

BreastF_entero_OR$logub<-log(BreastF_entero_OR$Num_ub)

BreastF_entero_OR$diflogCI<- BreastF_entero_OR$logub-BreastF_entero_OR$loglb

BreastF_entero_OR$logSE<-BreastF_entero_OR$diflogCI/3.92


or.breastf_entero<-rma(yi = (BreastF_entero_OR$logOR) , sei = (BreastF_entero_OR$logSE), method = "ML",measure = "OR")

summary(or.breastf_entero)


#S. pneumoniae

BreastF_spneumo<-subset(Breast_feeding, Breast_feeding$GroupGood=="Streptococcus pneumoniae")

BreastF_spneumo_OR<- subset(BreastF_spneumo, BreastF_spneumo$Method.of.estimation=="OR")


BreastF_spneumo_OR$logOR<-log(BreastF_spneumo_OR$Num_Estimate)

BreastF_spneumo_OR$loglb<-log(BreastF_spneumo_OR$Num_lb)

BreastF_spneumo_OR$logub<-log(BreastF_spneumo_OR$Num_ub)

BreastF_spneumo_OR$diflogCI<- BreastF_spneumo_OR$logub-BreastF_spneumo_OR$loglb

BreastF_spneumo_OR$logSE<-BreastF_spneumo_OR$diflogCI/3.92


or.breastf_spneumo<-rma(yi = (BreastF_spneumo_OR$logOR) , sei = (BreastF_spneumo_OR$logSE), method = "ML",measure = "OR")

summary(or.breastf_spneumo) #moet logor en log se nog toevoegen hier, helaas


# S. Areus

# This is for genes, so risk instead of odds method of calculation

breast_areus<-subset(Breast_feeding, Breast_feeding$GroupGood=="S. Aureus")

breast_areus<-subset(breast_areus, breast_areus$Method.of.estimation=="Genes")


breast_areus$id<-c(1,2)

breast_areus$Num_Estimate<- (as.numeric(gsub("[\\%,]","", breast_areus$Estimate)))/100
```

```
breast_areus$Sample.size<-as.numeric(as.character(breast_areus$Sample.size))

breast_areus$Events<-breast_areus$Sample.size*breast_areus$Num_Estimate

breast_areus$Events_integer<-round(breast_areus$Events)

breast_areus$No_Event<-breast_areus$Sample.size-breast_areus$Events_integer


model.breast.areus<-
glmer(Events_integer/Sample.size~1+(1|id),weights=Sample.size,data=breast_areus,family="binomi
al")

se.logit.breast<-aureus<-sqrt(vcov(model.breast.areus))

se.logit.breast


############################################################ Contact with infected person

# E.coli


Cont_Infeced_ecoli_Risk<-subset(Contact_with_infected,
Contact_with_infected$GroupGood=="E.Coli" &
Contact_with_infected$Method.of.estimation=="Risk")


Cont_Infeced_ecoli_Risk$id<-c(1:2)

Cont_Infeced_ecoli_Risk$Num_Estimate<- (as.numeric(gsub("[\\%,]","",
Cont_Infeced_ecoli_Risk$Estimate)))/100

Cont_Infeced_ecoli_Risk$Sample.size<-
as.numeric(as.character(Cont_Infeced_ecoli_Risk$Sample.size))

Cont_Infeced_ecoli_Risk$Events<-
Cont_Infeced_ecoli_Risk$Sample.size*Cont_Infeced_ecoli_Risk$Num_Estimate

Cont_Infeced_ecoli_Risk$Events_integer<-round(Cont_Infeced_ecoli_Risk$Events)

Cont_Infeced_ecoli_Risk$No_Event<-Cont_Infeced_ecoli_Risk$Sample.size-
Cont_Infeced_ecoli_Risk$Events_integer


model.cont.inf.ecoli_risk<-
glmer(Events_integer/Sample.size~1+(1|id),weights=Sample.size,data=Cont_Infeced_ecoli_Risk,fami
ly="binomial")

se.logit.cont.inf.ecoli.risk<-sqrt(vcov(model.cont.inf.ecoli_risk))
```

```
#Enterobacteria

# We will not report this one, too different estimates of routes

#Cont_Infeced_entero_genes<-subset(Contact_with_infected,
Contact_with_infected$GroupGood=="Enterobacteriaceae(multiple or unspecified)" &
Contact_with_infected$Method.of.estimation=="Genes")


#Cont_Infeced_entero_genes$id<-c(1:4)

#Cont_Infeced_entero_genes$Num_Estimate<- (as.numeric(gsub("[\\%,]","",
Cont_Infeced_entero_genes$Estimate)))/100

#Cont_Infeced_entero_genes$Sample.size<-
as.numeric(as.character(Cont_Infeced_entero_genes$Sample.size))

#Cont_Infeced_entero_genes$Events<-
Cont_Infeced_entero_genes$Sample.size*Cont_Infeced_entero_genes$Num_Estimate

#Cont_Infeced_entero_genes$Events_integer<-round(Cont_Infeced_entero_genes$Events)

#Cont_Infeced_entero_genes$No_Event<-Cont_Infeced_entero_genes$Sample.size-
Cont_Infeced_entero_genes$Events_integer


#model.cont.inf.entero<-
glmer(Events_integer/Sample.size~1+(1|id),weights=Sample.size,data=Cont_Infeced_entero_genes,
family="binomial")

#se.logit.cont.inf.entero<-sqrt(vcov(model.cont.inf.entero))


#S. Aureus

#Cases per day (4)

Contact_with_infected_Saurues_Cases<- subset(Contact_with_infected,
Contact_with_infected$GroupGood=="S. Aureus" &
Contact_with_infected$Method.of.estimation=="cases per day")


#Genes (14)

Contact_with_infected_Saurues_Genes<- subset(Contact_with_infected,
Contact_with_infected$GroupGood=="S. Aureus" &
Contact_with_infected$Method.of.estimation=="Genes")


Contact_with_infected_Saurues_Genes$id<-c(1:2)
```

```
Contact_with_infected_Saurues_Genes$Num_Estimate<- (as.numeric(gsub("[\\%,]","", Contact_with_infected_Saurues_Genes$Estimate)))/100

Contact_with_infected_Saurues_Genes$Sample.size<- as.numeric(as.character(Contact_with_infected_Saurues_Genes$Sample.size))

Contact_with_infected_Saurues_Genes$Events<- Contact_with_infected_Saurues_Genes$Sample.size*Contact_with_infected_Saurues_Genes$Num_Estimate

Contact_with_infected_Saurues_Genes$Events_integer<- round(Contact_with_infected_Saurues_Genes$Events)

Contact_with_infected_Saurues_Genes$No_Event<- Contact_with_infected_Saurues_Genes$Sample.size- Contact_with_infected_Saurues_Genes$Events_integer


model.cont.inf.saureus_genes<- glmer(Events_integer/Sample.size~1+(1|id),weights=Sample.size,data=Contact_with_infected_Saurues_Genes,family="binomial")

se.logit.cont.inf.saureus.genes<-sqrt(vcov(model.cont.inf.saureus_genes))


# Importance of Route  IOR(3)

Contact_with_infected_Saurues_IOR<- subset(Contact_with_infected, Contact_with_infected$GroupGood=="S. Aureus" & Contact_with_infected$Method.of.estimation=="Importance of Route")


Contact_with_infected_Saurues_IOR$id<-c(1:3)

Contact_with_infected_Saurues_IOR$Num_Estimate<- (as.numeric(gsub("[\\%,]","", Contact_with_infected_Saurues_IOR$Estimate)))

Contact_with_infected_Saurues_IOR$Num_Estimate[2]<- Contact_with_infected_Saurues_IOR$Num_Estimate[2]/100

Contact_with_infected_Saurues_IOR$Num_Estimate[1]<- Contact_with_infected_Saurues_IOR$Num_Estimate[1]/100

# Missing sample sizes so pooling not possible


#OR(2)

Contact_with_infected_Saurues_OR<- subset(Contact_with_infected, Contact_with_infected$GroupGood=="S. Aureus" & Contact_with_infected$Method.of.estimation=="OR")
```

```
Contact_with_infected_Saurues_OR$logOR<-
log(Contact_with_infected_Saurues_OR$Num_Estimate)

Contact_with_infected_Saurues_OR$loglb<-log(Contact_with_infected_Saurues_OR$Num_lb)

Contact_with_infected_Saurues_OR$logub<-log(Contact_with_infected_Saurues_OR$Num_ub)

Contact_with_infected_Saurues_OR$diflogCI<- Contact_with_infected_Saurues_OR$logub-
Contact_with_infected_Saurues_OR$loglb

Contact_with_infected_Saurues_OR$logSE<-Contact_with_infected_Saurues_OR$diflogCI/3.92


or.cont.inf.saureus<-rma(yi = (Contact_with_infected_Saurues_OR$logOR) , sei =
(Contact_with_infected_Saurues_OR$logSE), method = "ML",measure = "OR")

summary(or.cont.inf.saureus)


#R0(11)

Contact_with_infected_Saurues_r0<- subset(Contact_with_infected,
Contact_with_infected$GroupGood=="S. Aureus" &
Contact_with_infected$Method.of.estimation=="R0")

Contact_with_infected_Saurues_r0$Estimate[9]<-0.50

Contact_with_infected_Saurues_r0$Num_Estimate[9]<-0.50

Contact_with_infected_Saurues_r0$logr0<-log(Contact_with_infected_Saurues_r0$Num_Estimate)

Contact_with_infected_Saurues_r0$loglb<-log(Contact_with_infected_Saurues_r0$Num_lb)

Contact_with_infected_Saurues_r0$logub<-log(Contact_with_infected_Saurues_r0$Num_ub)

Contact_with_infected_Saurues_r0$diflogCI<- Contact_with_infected_Saurues_r0$logub-
Contact_with_infected_Saurues_r0$loglb

Contact_with_infected_Saurues_r0$logSE<-Contact_with_infected_Saurues_r0$diflogCI/3.92


r0.cont.inf.saureus<-rma(yi = (Contact_with_infected_Saurues_r0$logr0) , sei =
(Contact_with_infected_Saurues_r0$logSE), method = "ML",measure = "OR")

summary(r0.cont.inf.saureus)

exp(r0.cont.inf.saureus$beta)

view(Contact_with_infected_Saurues_r0)


#Risk(9)
```

```
Contact_with_infected_Saurues_Risk<- subset(Contact_with_infected,
Contact_with_infected$GroupGood=="S. Aureus" &
Contact_with_infected$Method.of.estimation=="Risk")


Contact_with_infected_Saurues_Risk$id<-c(1:8)

Contact_with_infected_Saurues_Risk$Num_Estimate<- (as.numeric(gsub("[\\%,]","",
Contact_with_infected_Saurues_Risk$Estimate)))/100

Contact_with_infected_Saurues_Risk$Sample.size<-
as.numeric(as.character(Contact_with_infected_Saurues_Risk$Sample.size))

Contact_with_infected_Saurues_Risk$Events<-
Contact_with_infected_Saurues_Risk$Sample.size*Contact_with_infected_Saurues_Risk$Num_Esti
mate

Contact_with_infected_Saurues_Risk$Events_integer<-
round(Contact_with_infected_Saurues_Risk$Events)

Contact_with_infected_Saurues_Risk$No_Event<-
Contact_with_infected_Saurues_Risk$Sample.size-
Contact_with_infected_Saurues_Risk$Events_integer


model.cont.inf.saureus_Risk<-
glmer(Events_integer/Sample.size~1+(1|id),weights=Sample.size,data=Contact_with_infected_Saur
ues_Risk,family="binomial")

se.logit.cont.inf.saureus.Risk<-sqrt(vcov(model.cont.inf.saureus_Risk))


#RR(2)

Contact_with_infected_Saurues_RR<- subset(Contact_with_infected,
Contact_with_infected$GroupGood=="S. Aureus" &
Contact_with_infected$Method.of.estimation=="RR")


Contact_with_infected_Saurues_RR$logRR<-
log(Contact_with_infected_Saurues_RR$Num_Estimate)

Contact_with_infected_Saurues_RR$loglb<-log(Contact_with_infected_Saurues_RR$Num_lb)

Contact_with_infected_Saurues_RR$logub<-log(Contact_with_infected_Saurues_RR$Num_ub)

Contact_with_infected_Saurues_RR$diflogCI<- Contact_with_infected_Saurues_RR$logub-
Contact_with_infected_Saurues_RR$loglb

Contact_with_infected_Saurues_RR$logSE<-Contact_with_infected_Saurues_RR$diflogCI/3.92
```

```
rr.contact.inf.saureus<-rma(yi = (Contact_with_infected_Saurues_RR$logRR) , sei =
(Contact_with_infected_Saurues_RR$logSE), method = "ML",measure = "RR")

summary(rr.contact.inf.saureus)


#TR(4)

Contact_with_infected_Saurues_TR<- subset(Contact_with_infected,
Contact_with_infected$GroupGood=="S. Aureus" &
Contact_with_infected$Method.of.estimation=="transmission rate")

# Cannot be pooled due to study asimilarites




#	VRE	17

Contact_with_infected_VRE<-subset(Contact_with_infected,
Contact_with_infected$GroupGood=="VRE")

table(Contact_with_infected_VRE$Method.of.estimation)


#Risk

Contact_with_infected_VRE_Risk<-
subset(Contact_with_infected_VRE,Contact_with_infected_VRE$Method.of.estimation=="Risk")


Contact_with_infected_VRE_Risk$id<-c(1:10)

Contact_with_infected_VRE_Risk$Num_Estimate<- (as.numeric(gsub("[\\%,]","",
Contact_with_infected_VRE_Risk$Estimate)))/100

Contact_with_infected_VRE_Risk$Sample.size<-
as.numeric(as.character(Contact_with_infected_VRE_Risk$Sample.size))

Contact_with_infected_VRE_Risk$Events<-
Contact_with_infected_VRE_Risk$Sample.size*Contact_with_infected_VRE_Risk$Num_Estimate

Contact_with_infected_VRE_Risk$Events_integer<-round(Contact_with_infected_VRE_Risk$Events)

Contact_with_infected_VRE_Risk$No_Event<-Contact_with_infected_VRE_Risk$Sample.size-
Contact_with_infected_VRE_Risk$Events_integer

Contact_with_infected_VRE_Risk$No_Event[10]<-408

Contact_with_infected_VRE_Risk$Events_integer[10]<-71
```

```
model.cont.inf.vre_Risk<-
glmer(Events_integer/Sample.size~1+(1|id),weights=Sample.size,data=Contact_with_infected_VRE_
Risk,family="binomial")

se.logit.cont.inf.vre.Risk<-sqrt(vcov(model.cont.inf.saureus_Risk))


#R0

Contact_with_infected_VRE_R0<-
subset(Contact_with_infected_VRE,Contact_with_infected_VRE$Method.of.estimation=="R0")

# Ook missing sample sizes


#        A. baumanni

#OR

Contact_with_infected_Abau_OR<-subset(Contact_with_infected,
Contact_with_infected$GroupGood=="Acinetobacter baumannii" &
Contact_with_infected$Method.of.estimation=="OR")


Contact_with_infected_Abau_OR$logOR<-log(Contact_with_infected_Abau_OR$Num_Estimate)

Contact_with_infected_Abau_OR$loglb<-log(Contact_with_infected_Abau_OR$Num_lb)

Contact_with_infected_Abau_OR$logub<-log(Contact_with_infected_Abau_OR$Num_ub)

Contact_with_infected_Abau_OR$diflogCI<- Contact_with_infected_Abau_OR$logub-
Contact_with_infected_Abau_OR$loglb

Contact_with_infected_Abau_OR$logSE<-Contact_with_infected_Abau_OR$diflogCI/3.92


or.cont.inf.abaum<-rma(yi = (Contact_with_infected_Abau_OR$logOR) , sei =
(Contact_with_infected_Abau_OR$logSE), method = "ML",measure = "OR")

summary(or.cont.inf.abaum)


#Risk

Contact_with_infected_Abau_Risk<-subset(Contact_with_infected,
Contact_with_infected$GroupGood=="Acinetobacter baumannii" &
Contact_with_infected$Method.of.estimation=="Risk")


Contact_with_infected_Abau_Risk$id<-c(1:5)
```

```
Contact_with_infected_Abau_Risk$Num_Estimate<- (as.numeric(gsub("[\\%,]","",
Contact_with_infected_Abau_Risk$Estimate)))/100

Contact_with_infected_Abau_Risk$Sample.size<-
as.numeric(as.character(Contact_with_infected_Abau_Risk$Sample.size))

Contact_with_infected_Abau_Risk$Events<-
Contact_with_infected_Abau_Risk$Sample.size*Contact_with_infected_Abau_Risk$Num_Estimate

Contact_with_infected_Abau_Risk$Events_integer<-
round(Contact_with_infected_Abau_Risk$Events)

Contact_with_infected_Abau_Risk$No_Event<-Contact_with_infected_Abau_Risk$Sample.size-
Contact_with_infected_Abau_Risk$Events_integer


model.cont.inf.abau_Risk<-
glmer(Events_integer/Sample.size~1+(1|id),weights=Sample.size,data=Contact_with_infected_Abau
_Risk,family="binomial")

se.logit.cont.inf.abau<-sqrt(vcov(model.cont.inf.abau_Risk))


#R0

Contact_with_infected_Abau_R0<-subset(Contact_with_infected,
Contact_with_infected$GroupGood=="Acinetobacter baumannii" &
Contact_with_infected$Method.of.estimation=="R0")

# Same study but per day and per hospital admission so not able to pool


# p. aeruginosa

# Risk (2)

Contact_with_infected_paerug_risk<-subset(Contact_with_infected,
Contact_with_infected$GroupGood=="Pseudomonas aeruginosa" &
Contact_with_infected$Method.of.estimation=="Risk")


Contact_with_infected_paerug_risk$id<-c(1,2)

Contact_with_infected_paerug_risk$Num_Estimate<- (as.numeric(gsub("[\\%,]","",
Contact_with_infected_paerug_risk$Estimate)))/100

Contact_with_infected_paerug_risk$Sample.size<-
as.numeric(as.character(Contact_with_infected_paerug_risk$Sample.size))

Contact_with_infected_paerug_risk$Events<-
Contact_with_infected_paerug_risk$Sample.size*Contact_with_infected_paerug_risk$Num_Estimat
e
```

```
Contact_with_infected_paerug_risk$Events_integer<-
round(Contact_with_infected_paerug_risk$Events)

Contact_with_infected_paerug_risk$No_Event<-Contact_with_infected_paerug_risk$Sample.size-
Contact_with_infected_paerug_risk$Events_integer


model.cont.inf.pau<-
glmer(Events_integer/Sample.size~1+(1|id),weights=Sample.size,data=Contact_with_infected_paer
ug_risk,family="binomial")

se.logit.cont.inf.pau<-sqrt(vcov(model.cont.inf.pau))


#Genes (2)

Contact_with_infected_paerug_genes<- subset(Contact_with_infected,
Contact_with_infected$GroupGood=="Pseudomonas aeruginosa" &
Contact_with_infected$Method.of.estimation=="Genes")


Contact_with_infected_paerug_genes$id<-c(1:2)

Contact_with_infected_paerug_genes$Num_Estimate<- (as.numeric(gsub("[\\%,]","",
Contact_with_infected_paerug_genes$Estimate)))/100

Contact_with_infected_paerug_genes$Sample.size<-
as.numeric(as.character(Contact_with_infected_paerug_genes$Sample.size))

Contact_with_infected_paerug_genes$Events<-
Contact_with_infected_paerug_genes$Sample.size*Contact_with_infected_paerug_genes$Num_Est
imate

Contact_with_infected_paerug_genes$Events_integer<-
round(Contact_with_infected_paerug_genes$Events)

Contact_with_infected_paerug_genes$No_Event<-
Contact_with_infected_paerug_genes$Sample.size-
Contact_with_infected_paerug_genes$Events_integer


model.cont.inf.pau.genes<-
glmer(Events_integer/Sample.size~1+(1|id),weights=Sample.size,data=Contact_with_infected_paer
ug_genes,family="binomial")

se.logit.cont.inf.pau.genes<-sqrt(vcov(model.cont.inf.pau.genes))


#IOR (2)
```

```
Contact_with_infected_paerug_IOR<-subset(Contact_with_infected,
Contact_with_infected$GroupGood=="Pseudomonas aeruginosa" &
Contact_with_infected$Method.of.estimation=="Importance of Route")


Contact_with_infected_paerug_IOR$id<-c(1,2)

Contact_with_infected_paerug_IOR$Num_Estimate<- (as.numeric(gsub("[\\%,]","",
Contact_with_infected_paerug_IOR$Estimate)))

Contact_with_infected_paerug_IOR$Num_Estimate[1]<-
Contact_with_infected_paerug_IOR$Num_Estimate[1]/100

Contact_with_infected_paerug_IOR$Sample.size<-
as.numeric(as.character(Contact_with_infected_paerug_IOR$Sample.size))

#Sample size unknown


#	S. epidermidis

#Risk(2)

Contact_with_infected_epi_risk<-subset(Contact_with_infected,
Contact_with_infected$GroupGood=="Staphylococcus epidermidis")


Contact_with_infected_epi_risk$id<-c(1,2)

Contact_with_infected_epi_risk$Num_Estimate<- (as.numeric(gsub("[\\%,]","",
Contact_with_infected_epi_risk$Estimate)))/100

Contact_with_infected_epi_risk$Sample.size<-
as.numeric(as.character(Contact_with_infected_epi_risk$Sample.size))

Contact_with_infected_epi_risk$Events<-
Contact_with_infected_epi_risk$Sample.size*Contact_with_infected_epi_risk$Num_Estimate

Contact_with_infected_epi_risk$Events_integer<-round(Contact_with_infected_epi_risk$Events)

Contact_with_infected_epi_risk$No_Event<-Contact_with_infected_epi_risk$Sample.size-
Contact_with_infected_epi_risk$Events_integer


model.cont.inf.epi.risk<-
glmer(Events_integer/Sample.size~1+(1|id),weights=Sample.size,data=Contact_with_infected_epi_r
isk,family="binomial")

se.logit.cont.inf.epi.risk<-sqrt(vcov(model.cont.inf.epi.risk))
```

```
########################################################## Eating meat to human

Eating_meat_E.coli_OR<-subset(Eating_meat, Eating_meat$GroupGood=="E.Coli" & Eating_meat$Method.of.estimation=="OR")

Eating_meat_E.coli_OR<-Eating_meat_E.coli_OR[c(1:4,6:12),]

Eating_meat_E.coli_PR<-subset(Eating_meat, Eating_meat$GroupGood=="E.Coli" & Eating_meat$Method.of.estimation=="PR")

Eating_meat_E.coli_PR<-Eating_meat_E.coli_PR[c(1,4,6:8),]


Eating_meat_E.coli_OR$logOR<-log(Eating_meat_E.coli_OR$Num_Estimate)

Eating_meat_E.coli_OR$loglb<-log(Eating_meat_E.coli_OR$Num_lb)

Eating_meat_E.coli_OR$logub<-log(Eating_meat_E.coli_OR$Num_ub)

Eating_meat_E.coli_OR$diflogCI<- Eating_meat_E.coli_OR$logub-Eating_meat_E.coli_OR$loglb

Eating_meat_E.coli_OR$logSE<-Eating_meat_E.coli_OR$diflogCI/3.92


or.meat_ecoli<-rma(yi = (Eating_meat_E.coli_OR$logOR) , sei = (Eating_meat_E.coli_OR$logSE), method = "ML",measure = "OR")

summary(or.meat_ecoli)


#view(Eating_meat)


# meat stratified

whitemeat_or<-Eating_meat_E.coli_OR[c(4,8),]

redmeat_or<-Eating_meat_E.coli_OR[c(1:3,9:11),]

generalmeat_or<-Eating_meat_E.coli_OR[(5:7),]

or.white<-rma(yi = (whitemeat_or$logOR) , sei = (whitemeat_or$logSE), method = "ML",measure = "OR")

or.red<-rma(yi = (redmeat_or$logOR) , sei = (redmeat_or$logSE), method = "ML",measure = "OR")

or.general<-rma(yi = (generalmeat_or$logOR) , sei = (generalmeat_or$logSE), method = "ML",measure = "OR")



Eating_meat_E.coli_PR$logOR<-log(Eating_meat_E.coli_PR$Num_Estimate)

Eating_meat_E.coli_PR$loglb<-log(Eating_meat_E.coli_PR$Num_lb)
```

```
Eating_meat_E.coli_PR$logub<-log(Eating_meat_E.coli_PR$Num_ub)

Eating_meat_E.coli_PR$diflogCI<- Eating_meat_E.coli_PR$logub-Eating_meat_E.coli_PR$loglb

Eating_meat_E.coli_PR$logSE<-Eating_meat_E.coli_PR$diflogCI/3.92


pr.meat_ecoli<-rma(yi = (Eating_meat_E.coli_PR$logOR) , sei = (Eating_meat_E.coli_PR$logSE), method = "ML",measure = "RR")

summary(pr.meat_ecoli)

# meat stratified


Eating_meat_E.coli_PR$Num_Estimate[1]<-1.07

red_pr<-Eating_meat_E.coli_PR[c(1,2,4,5),]


red_pr$logOR<-log(red_pr$Num_Estimate)

red_pr$loglb<-log(red_pr$Num_lb)

red_pr$logub<-log(red_pr$Num_ub)

red_pr$diflogCI<- red_pr$logub-red_pr$loglb

red_pr$logSE<-red_pr$diflogCI/3.92


pr.redmeat<-rma(yi = (red_pr$logOR) , sei = (red_pr$logSE), method = "ML",measure = "RR")


############################################################ Family member colonised

#E.coli

#Risk

Family_member_colonised_ecoli_risk<-subset(Family_member_colonised, Family_member_colonised$GroupGood=="E.Coli" & Family_member_colonised$Method.of.estimation=="Risk")

Family_member_colonised_ecoli_risk$id<-c(1:2)

Family_member_colonised_ecoli_risk$Num_Estimate<- (as.numeric(gsub("[\\%,]","", Family_member_colonised_ecoli_risk$Estimate)))/100

Family_member_colonised_ecoli_risk$Sample.size<- as.numeric(as.character(Family_member_colonised_ecoli_risk$Sample.size))
```

Family_member_colonised_ecoli_risk$Events<-Family_member_colonised_ecoli_risk$Sample.size*Family_member_colonised_ecoli_risk$Num_Estimate

Family_member_colonised_ecoli_risk$Events_integer<-round(Family_member_colonised_ecoli_risk$Events)

Family_member_colonised_ecoli_risk$No_Event<-Family_member_colonised_ecoli_risk$Sample.size-Family_member_colonised_ecoli_risk$Events_integer

model.Family_member_colonised_ecoli_risk<-glmer(Events_integer/Sample.size~1+(1|id),weights=Sample.size,data=Family_member_colonised_ecoli_risk,family="binomial")

se.logit.Family_member_colonised_ecoli_risk<-sqrt(vcov(model.Family_member_colonised_ecoli_risk))

#OR

Family_member_colonised_ecoli_OR<-subset(Family_member_colonised, Family_member_colonised$GroupGood=="E.Coli" & Family_member_colonised$Method.of.estimation=="OR")

Family_member_colonised_ecoli_OR$logOR<-log(Family_member_colonised_ecoli_OR$Num_Estimate)

Family_member_colonised_ecoli_OR$loglb<-log(Family_member_colonised_ecoli_OR$Num_lb)

Family_member_colonised_ecoli_OR$logub<-log(Family_member_colonised_ecoli_OR$Num_ub)

Family_member_colonised_ecoli_OR$diflogCI<- Family_member_colonised_ecoli_OR$logub-Family_member_colonised_ecoli_OR$loglb

Family_member_colonised_ecoli_OR$logSE<-Family_member_colonised_ecoli_OR$diflogCI/3.92

or.fam.mem.col.ecoli<-rma(yi = (Family_member_colonised_ecoli_OR$logOR) , sei = (Family_member_colonised_ecoli_OR$logSE), method = "ML",measure = "RR")

summary(or.fam.mem.col.ecoli)

Genes

Fam_col_ecoli_genes<-subset(Family_member_colonised, Family_member_colonised$GroupGood=="E.Coli" & Family_member_colonised$Method.of.estimation=="Genes")

```
Fam_col_ecoli_genes$id<-c(1:3)

Fam_col_ecoli_genes$Num_Estimate<- (as.numeric(gsub("[\\%,]","",
Fam_col_ecoli_genes$Estimate)))/100

Fam_col_ecoli_genes$Sample.size<-as.numeric(as.character(Fam_col_ecoli_genes$Sample.size))

Fam_col_ecoli_genes$Events<-
Fam_col_ecoli_genes$Sample.size*Fam_col_ecoli_genes$Num_Estimate

Fam_col_ecoli_genes$Events_integer<-round(Fam_col_ecoli_genes$Events)

Fam_col_ecoli_genes$No_Event<-Fam_col_ecoli_genes$Sample.size-
Fam_col_ecoli_genes$Events_integer


model.Fam_col_ecoli_genes<-
glmer(Events_integer/Sample.size~1+(1|id),weights=Sample.size,data=Fam_col_ecoli_genes,family=
"binomial")

se.logit.Fam_col_ecoli_genes<-sqrt(vcov(model.Fam_col_ecoli_genes))


#Entero

Family_member_colonised_entero_PR<-subset(Family_member_colonised,
Family_member_colonised$GroupGood=="Enterobacteriaceae(multiple or unspecified)" &
Family_member_colonised$Method.of.estimation=="PR")


Family_member_colonised_entero_PR$logOR<-
log(Family_member_colonised_entero_PR$Num_Estimate)

Family_member_colonised_entero_PR$loglb<-log(Family_member_colonised_entero_PR$Num_lb)

Family_member_colonised_entero_PR$logub<-log(Family_member_colonised_entero_PR$Num_ub)

Family_member_colonised_entero_PR$diflogCI<- Family_member_colonised_entero_PR$logub-
Family_member_colonised_entero_PR$loglb

Family_member_colonised_entero_PR$logSE<-Family_member_colonised_entero_PR$diflogCI/3.92


pr.fam.mem.col.enter<-rma(yi = (Family_member_colonised_entero_PR$logOR) , sei =
(Family_member_colonised_entero_PR$logSE), method = "ML",measure = "RR")

summary(pr.fam.mem.col.enter)


# S. aureus
```

```
Family_member_colonised_saureus_OR<-subset(Family_member_colonised,
Family_member_colonised$GroupGood=="S. Aureus" &
Family_member_colonised$Method.of.estimation=="OR")

Family_member_colonised_saureus_OR<-Family_member_colonised_saureus_OR[c(1:6),]

Family_member_colonised_saureus_OR$logOR<-
log(Family_member_colonised_saureus_OR$Num_Estimate)

Family_member_colonised_saureus_OR$loglb<-
log(Family_member_colonised_saureus_OR$Num_lb)

Family_member_colonised_saureus_OR$logub<-
log(Family_member_colonised_saureus_OR$Num_ub)

Family_member_colonised_saureus_OR$diflogCI<- Family_member_colonised_saureus_OR$logub-
Family_member_colonised_saureus_OR$loglb

Family_member_colonised_saureus_OR$logSE<-
Family_member_colonised_saureus_OR$diflogCI/3.92


or.fam.mem.col.saureus<-rma(yi = (Family_member_colonised_saureus_OR$logOR) , sei =
(Family_member_colonised_saureus_OR$logSE), method = "ML",measure = "RR")

summary(or.fam.mem.col.saureus)

# Risk

fam_col_sau_risk<-subset(Family_member_colonised, Family_member_colonised$GroupGood=="S.
Aureus" & Family_member_colonised$Method.of.estimation=="Risk")


fam_col_sau_risk<-fam_col_sau_risk[c(1,3,4),]

fam_col_sau_risk$id<-c(1,3,4)

fam_col_sau_risk$Num_Estimate<- (as.numeric(gsub("[\\%,]","", fam_col_sau_risk$Estimate)))/100

fam_col_sau_risk$Sample.size<-as.numeric(as.character(fam_col_sau_risk$Sample.size))

fam_col_sau_risk$Events<-fam_col_sau_risk$Sample.size*fam_col_sau_risk$Num_Estimate

fam_col_sau_risk$Events_integer<-round(fam_col_sau_risk$Events)

fam_col_sau_risk$No_Event<-fam_col_sau_risk$Sample.size-fam_col_sau_risk$Events_integer


model.fam_col_sau_risk<-
glmer(Events_integer/Sample.size~1+(1|id),weights=Sample.size,data=fam_col_sau_risk,family="bin
omial")

se.logit.fam_col_sau_risk<-sqrt(vcov(model.fam_col_sau_risk))

#view(fam_col_sau_risk)
```

```
# genes

fam_col_sau_genes<-subset(Family_member_colonised,
Family_member_colonised$GroupGood=="S. Aureus" &
Family_member_colonised$Method.of.estimation=="Genes")

fam_col_sau_genes_T<-fam_col_sau_genes[c(2,7:9),]

fam_col_sau_genes<-fam_col_sau_genes[c(1,3:6,10:11),]


fam_col_sau_genes$id<-c(1:7)

fam_col_sau_genes$Num_Estimate<- (as.numeric(gsub("[\\%,]","",
fam_col_sau_genes$Estimate)))/100

fam_col_sau_genes$Sample.size<-as.numeric(as.character(fam_col_sau_genes$Sample.size))

fam_col_sau_genes$Events<-fam_col_sau_genes$Sample.size*fam_col_sau_genes$Num_Estimate

fam_col_sau_genes$Events_integer<-round(fam_col_sau_genes$Events)

fam_col_sau_genes$No_Event<-fam_col_sau_genes$Sample.size-
fam_col_sau_genes$Events_integer


model.fam_col_sau_genes<-
glmer(Events_integer/Sample.size~1+(1|id),weights=Sample.size,data=fam_col_sau_genes,family="
binomial")

se.logit.fam_col_sau_genes<-sqrt(vcov(model.fam_col_sau_genes))

#view(fam_col_sau_genes)


fam_col_sau_genes_T$id<-c(1:4)

fam_col_sau_genes_T$Num_Estimate<- (as.numeric(gsub("[\\%,]","",
fam_col_sau_genes_T$Estimate)))/100

fam_col_sau_genes_T$Sample.size<-as.numeric(as.character(fam_col_sau_genes_T$Sample.size))

fam_col_sau_genes_T$Events<-
fam_col_sau_genes_T$Sample.size*fam_col_sau_genes_T$Num_Estimate

fam_col_sau_genes_T$Events_integer<-round(fam_col_sau_genes_T$Events)

fam_col_sau_genes_T$No_Event<-fam_col_sau_genes_T$Sample.size-
fam_col_sau_genes_T$Events_integer
```

```
model.fam_col_sau_genes_T<-
glmer(Events_integer/Sample.size~1+(1|id),weights=Sample.size,data=fam_col_sau_genes_T,family
="binomial")

se.logit.fam_col_sau_genes_T<-sqrt(vcov(model.fam_col_sau_genes_T))


#view(fam_col_sau_genes_T)


# p aeruginose


#Genes

Fam_with_infected_paerug_genes<- subset(Family_member_colonised,
Family_member_colonised$GroupGood=="Pseudomonas aeruginosa" &
Family_member_colonised$Method.of.estimation=="Genes")


Fam_with_infected_paerug_genes$id<-c(1:2)

Fam_with_infected_paerug_genes$Num_Estimate<- (as.numeric(gsub("[\\%,]","",
Fam_with_infected_paerug_genes$Estimate)))/100

Fam_with_infected_paerug_genes$Sample.size<-
as.numeric(as.character(Fam_with_infected_paerug_genes$Sample.size))

Fam_with_infected_paerug_genes$Events<-
Fam_with_infected_paerug_genes$Sample.size*Fam_with_infected_paerug_genes$Num_Estimate

Fam_with_infected_paerug_genes$Events_integer<-
round(Fam_with_infected_paerug_genes$Events)

Fam_with_infected_paerug_genes$No_Event<-Fam_with_infected_paerug_genes$Sample.size-
Fam_with_infected_paerug_genes$Events_integer


model.fam.pau.genes<-
glmer(Events_integer/Sample.size~1+(1|id),weights=Sample.size,data=Fam_with_infected_paerug_
genes,family="binomial")

se.logit.fam.pau.genes<-sqrt(vcov(model.fam.pau.genes))


############################################################ Family member occupational
exposure

Family_member_occupational_exposure_VRE<-subset(Family_member_occupational_exposure,
Family_member_occupational_exposure$GroupGood=="VRE")
```

```
Family_member_occupational_exposure_saureus<-subset(Family_member_occupational_exposure, Family_member_occupational_exposure$GroupGood=="S. Aureus" & Family_member_occupational_exposure$Method.of.estimation=="OR")

Family_member_occupational_exposure_saureus_hospital<-Family_member_occupational_exposure_saureus[c(4,5),]

Family_member_occupational_exposure_saureus_farming<-Family_member_occupational_exposure_saureus[c(1,2,3,7),]


Family_member_occupational_exposure_saureus_farming$logOR<-log(Family_member_occupational_exposure_saureus_farming$Num_Estimate)

Family_member_occupational_exposure_saureus_farming$loglb<-log(Family_member_occupational_exposure_saureus_farming$Num_lb)

Family_member_occupational_exposure_saureus_farming$logub<-log(Family_member_occupational_exposure_saureus_farming$Num_ub)

Family_member_occupational_exposure_saureus_farming$diflogCI<-Family_member_occupational_exposure_saureus_farming$logub-Family_member_occupational_exposure_saureus_farming$loglb

Family_member_occupational_exposure_saureus_farming$logSE<-Family_member_occupational_exposure_saureus_farming$diflogCI/3.92


or.fam.occu.farm<-rma(yi = (Family_member_occupational_exposure_saureus_farming$logOR) , sei = (Family_member_occupational_exposure_saureus_farming$logSE), method = "ML",measure = "RR")

summary(or.fam.occu.farm)


Family_member_occupational_exposure_saureus_hospital$logOR<-log(Family_member_occupational_exposure_saureus_hospital$Num_Estimate)

Family_member_occupational_exposure_saureus_hospital$loglb<-log(Family_member_occupational_exposure_saureus_hospital$Num_lb)

Family_member_occupational_exposure_saureus_hospital$logub<-log(Family_member_occupational_exposure_saureus_hospital$Num_ub)

Family_member_occupational_exposure_saureus_hospital$diflogCI<-Family_member_occupational_exposure_saureus_hospital$logub-Family_member_occupational_exposure_saureus_hospital$loglb

Family_member_occupational_exposure_saureus_hospital$logSE<-Family_member_occupational_exposure_saureus_hospital$diflogCI/3.92
```

```
or.fam.occu.hosp<-rma(yi = (Family_member_occupational_exposure_saureus_hospital$logOR) , sei = (Family_member_occupational_exposure_saureus_hospital$logSE), method = "ML",measure = "RR")

summary(or.fam.occu.hosp)


############################################################ Human to nearby environement

Human_to_nearbyenv_Saureus_Risk<-subset(Human_to_nearbyenv, Human_to_nearbyenv$GroupGood=="S. Aureus" & Human_to_nearbyenv$Method.of.estimation=="Risk")


Human_to_nearbyenv_Saureus_Risk$id<-c(1:3)

Human_to_nearbyenv_Saureus_Risk$Num_Estimate<- (as.numeric(gsub("[\\%,]","", Human_to_nearbyenv_Saureus_Risk$Estimate)))/100

Human_to_nearbyenv_Saureus_Risk$Sample.size<-as.numeric(as.character(Human_to_nearbyenv_Saureus_Risk$Sample.size))

Human_to_nearbyenv_Saureus_Risk$Events<-Human_to_nearbyenv_Saureus_Risk$Sample.size*Human_to_nearbyenv_Saureus_Risk$Num_Estimate

Human_to_nearbyenv_Saureus_Risk$Events_integer<-round(Human_to_nearbyenv_Saureus_Risk$Events)

Human_to_nearbyenv_Saureus_Risk$No_Event<-Human_to_nearbyenv_Saureus_Risk$Sample.size-Human_to_nearbyenv_Saureus_Risk$Events_integer


model.humantonearenv.saureus.risk<-glmer(Events_integer/Sample.size~1+(1|id),weights=Sample.size,data=Human_to_nearbyenv_Saureus_Risk,family="binomial")

se.logithumantonearenv.saureus.risk<-sqrt(vcov(model.humantonearenv.saureus.risk))


Human_to_nearbyenv_VRE_Risk<-subset(Human_to_nearbyenv, Human_to_nearbyenv$GroupGood=="VRE" & Human_to_nearbyenv$Method.of.estimation=="Risk")


Human_to_nearbyenv_VRE_Risk<-Human_to_nearbyenv_VRE_Risk[c(1:7),]

Human_to_nearbyenv_VRE_Risk$id<-c(1:7)

Human_to_nearbyenv_VRE_Risk$Num_Estimate<- (as.numeric(gsub("[\\%,]","", Human_to_nearbyenv_VRE_Risk$Estimate)))/100
```

```
Human_to_nearbyenv_VRE_Risk$Sample.size<-
as.numeric(as.character(Human_to_nearbyenv_VRE_Risk$Sample.size))

Human_to_nearbyenv_VRE_Risk$Events<-
Human_to_nearbyenv_VRE_Risk$Sample.size*Human_to_nearbyenv_VRE_Risk$Num_Estimate

Human_to_nearbyenv_VRE_Risk$Events_integer<-round(Human_to_nearbyenv_VRE_Risk$Events)

Human_to_nearbyenv_VRE_Risk$No_Event<-Human_to_nearbyenv_VRE_Risk$Sample.size-
Human_to_nearbyenv_VRE_Risk$Events_integer


model.humantonearenv.VRE.risk<-
glmer(Events_integer/Sample.size~1+(1|id),weights=Sample.size,data=Human_to_nearbyenv_VRE_
Risk,family="binomial")

se.logithumantonearenv.VRE.risk<-sqrt(vcov(model.humantonearenv.VRE.risk))


# r0

R0_VRE_humantonrear<-subset(Human_to_nearbyenv, Human_to_nearbyenv$GroupGood=="VRE"
& Human_to_nearbyenv$Method.of.estimation=="R0")

#missing sample sizes


#risk

Human_to_nearbyenv_abau_Risk<-subset(Human_to_nearbyenv,
Human_to_nearbyenv$GroupGood=="Acinetobacter baumannii" &
Human_to_nearbyenv$Method.of.estimation=="Risk")


Human_to_nearbyenv_abau_Risk$id<-c(1:13)

Human_to_nearbyenv_abau_Risk$Num_Estimate<- (as.numeric(gsub("[\\%,]","",
Human_to_nearbyenv_abau_Risk$Estimate)))/100

Human_to_nearbyenv_abau_Risk$Sample.size<-
as.numeric(as.character(Human_to_nearbyenv_abau_Risk$Sample.size))

Human_to_nearbyenv_abau_Risk$Events<-
Human_to_nearbyenv_abau_Risk$Sample.size*Human_to_nearbyenv_abau_Risk$Num_Estimate

Human_to_nearbyenv_abau_Risk$Events_integer<-
round(Human_to_nearbyenv_abau_Risk$Events)

Human_to_nearbyenv_abau_Risk$No_Event<-Human_to_nearbyenv_abau_Risk$Sample.size-
Human_to_nearbyenv_abau_Risk$Events_integer
```

```
model.humantonearenv.abau.risk<-glmer(Events_integer/Sample.size~1+(1|id),weights=Sample.size,data=Human_to_nearbyenv_abau_Risk,family="binomial")

se.logithumantonearenv.abau.risk<-sqrt(vcov(model.humantonearenv.abau.risk))


Human_to_nearbyenv_acalco_Risk<-subset(Human_to_nearbyenv, Human_to_nearbyenv$GroupGood=="Acinetobacter calcoaceticus" & Human_to_nearbyenv$Method.of.estimation=="Risk")


Human_to_nearbyenv_acalco_Risk$id<-c(1:2)

Human_to_nearbyenv_acalco_Risk$Num_Estimate<- (as.numeric(gsub("[\\%,]","", Human_to_nearbyenv_acalco_Risk$Estimate)))/100

Human_to_nearbyenv_acalco_Risk$Sample.size<-as.numeric(as.character(Human_to_nearbyenv_acalco_Risk$Sample.size))

Human_to_nearbyenv_acalco_Risk$Events<-Human_to_nearbyenv_acalco_Risk$Sample.size*Human_to_nearbyenv_acalco_Risk$Num_Estimate

Human_to_nearbyenv_acalco_Risk$Events_integer<-round(Human_to_nearbyenv_acalco_Risk$Events)

Human_to_nearbyenv_acalco_Risk$No_Event<-Human_to_nearbyenv_acalco_Risk$Sample.size-Human_to_nearbyenv_acalco_Risk$Events_integer


model.humantonearenv.acalco.risk<-glmer(Events_integer/Sample.size~1+(1|id),weights=Sample.size,data=Human_to_nearbyenv_acalco_Risk,family="binomial")

se.logithumantonearenv.acalco.risk<-sqrt(vcov(model.humantonearenv.acalco.risk))


############################################################ Livestock to drinking water

Livestock_to_drinking_water_pig<-subset(Livestock_to_drinking_water, Livestock_to_drinking_water$Animal.involved.in.route=="pig")

Livestock_to_drinking_water_poultry<-subset(Livestock_to_drinking_water, Livestock_to_drinking_water$Animal.involved.in.route=="poultry")

Livestock_to_drinking_water_cattle<-subset(Livestock_to_drinking_water, Livestock_to_drinking_water$Animal.involved.in.route=="cattle" | Livestock_to_drinking_water$Animal.involved.in.route=="catle")


Livestock_to_drinking_water_pig$logOR<-log(Livestock_to_drinking_water_pig$Num_Estimate)
```

```
Livestock_to_drinking_water_pig$loglb<-log(Livestock_to_drinking_water_pig$Num_lb)

Livestock_to_drinking_water_pig$logub<-log(Livestock_to_drinking_water_pig$Num_ub)

Livestock_to_drinking_water_pig$diflogCI<-Livestock_to_drinking_water_pig$logub-
Livestock_to_drinking_water_pig$loglb

Livestock_to_drinking_water_pig$logSE<-Livestock_to_drinking_water_pig$diflogCI/3.92


or.livedrink_pig<-rma(yi = (Livestock_to_drinking_water_pig$logOR) , sei =
(Livestock_to_drinking_water_pig$logSE), method = "ML",measure = "RR")

summary(or.livedrink_pig)


Livestock_to_drinking_water_poultry$logOR<-
log(Livestock_to_drinking_water_poultry$Num_Estimate)

Livestock_to_drinking_water_poultry$loglb<-log(Livestock_to_drinking_water_poultry$Num_lb)

Livestock_to_drinking_water_poultry$logub<-log(Livestock_to_drinking_water_poultry$Num_ub)

Livestock_to_drinking_water_poultry$diflogCI<- Livestock_to_drinking_water_poultry$logub-
Livestock_to_drinking_water_poultry$loglb

Livestock_to_drinking_water_poultry$logSE<-Livestock_to_drinking_water_poultry$diflogCI/3.92


or.livedrink_poultry<-rma(yi = (Livestock_to_drinking_water_poultry$logOR) , sei =
(Livestock_to_drinking_water_poultry$logSE), method = "ML",measure = "RR")

summary(or.livedrink_poultry)


Livestock_to_drinking_water_cattle$logOR<-
log(Livestock_to_drinking_water_cattle$Num_Estimate)

Livestock_to_drinking_water_cattle$loglb<-log(Livestock_to_drinking_water_cattle$Num_lb)

Livestock_to_drinking_water_cattle$logub<-log(Livestock_to_drinking_water_cattle$Num_ub)

Livestock_to_drinking_water_cattle$diflogCI<- Livestock_to_drinking_water_cattle$logub-
Livestock_to_drinking_water_cattle$loglb

Livestock_to_drinking_water_cattle$logSE<-Livestock_to_drinking_water_cattle$diflogCI/3.92


or.livedrink_cattle<-rma(yi = (Livestock_to_drinking_water_cattle$logOR) , sei =
(Livestock_to_drinking_water_cattle$logSE), method = "ML",measure = "RR")

summary(or.livedrink_cattle)

##########################################################Mother to child
```

```
Mother_to_child_entero_Risk<-subset(Mother_to_child,
Mother_to_child$GroupGood=="Enterobacteriaceae(multiple or unspecified)" &
Mother_to_child$Method.of.estimation=="Risk")

#don't pool because not similar


Mother_to_child_Saureus_Risk<-subset(Mother_to_child, Mother_to_child$GroupGood=="S.
Aureus" & Mother_to_child$Method.of.estimation=="Risk")


Mother_to_child_Saureus_Risk$id<-c(1:3)

Mother_to_child_Saureus_Risk$Num_Estimate<- (as.numeric(gsub("[\\%,]","",
Mother_to_child_Saureus_Risk$Estimate)))/100

Mother_to_child_Saureus_Risk$Sample.size<-as.numeric(as.character(
Mother_to_child_Saureus_Risk$Sample.size))

Mother_to_child_Saureus_Risk$Events<- Mother_to_child_Saureus_Risk$Sample.size*
Mother_to_child_Saureus_Risk$Num_Estimate

Mother_to_child_Saureus_Risk$Events_integer<-round( Mother_to_child_Saureus_Risk$Events)

Mother_to_child_Saureus_Risk$No_Event<- Mother_to_child_Saureus_Risk$Sample.size-
Mother_to_child_Saureus_Risk$Events_integer


model.mother.to.child.risk<-glmer(Events_integer/Sample.size~1+(1|id),weights=Sample.size,data=
Mother_to_child_Saureus_Risk,family="binomial")

se.logit.mother.to.child<-sqrt(vcov(model.mother.to.child.risk))


Mother_to_child_Aaureus_OR<-subset(Mother_to_child, Mother_to_child$GroupGood=="S.
Aureus" & Mother_to_child$Method.of.estimation=="OR")


Mother_to_child_Aaureus_OR$logOR<-log(Mother_to_child_Aaureus_OR$Num_Estimate)

Mother_to_child_Aaureus_OR$loglb<-log(Mother_to_child_Aaureus_OR$Num_lb)

Mother_to_child_Aaureus_OR$logub<-log(Mother_to_child_Aaureus_OR$Num_ub)

Mother_to_child_Aaureus_OR$diflogCI<- Mother_to_child_Aaureus_OR$logub-
Mother_to_child_Aaureus_OR$loglb

Mother_to_child_Aaureus_OR$logSE<-Mother_to_child_Aaureus_OR$diflogCI/3.92
```

```
or.mother.to.child<-rma(yi = (Mother_to_child_Aaureus_OR$logOR) , sei =
(Mother_to_child_Aaureus_OR$logSE), method = "ML",measure = "RR")

summary(or.livedrink_cattle)


# group B


Mother_to_child_groupB_Risk<-subset(Mother_to_child,  Mother_to_child$GroupGood=="Group B
streptococci" & Mother_to_child$Method.of.estimation=="Risk")

Mother_to_child_groupB_Risk<-
Mother_to_child_groupB_Risk[!(Mother_to_child_groupB_Risk$Author=="Facchinetti F"),]

#The study of Facchinetti et al is not comparable to the other studies and therefore not included in
the meta-analysis

Mother_to_child_groupB_Risk$id<-c(1:5)

Mother_to_child_groupB_Risk$Num_Estimate<- (as.numeric(gsub("[\\%,]","",
Mother_to_child_groupB_Risk$Estimate)))/100

Mother_to_child_groupB_Risk$Sample.size<-as.numeric(as.character(
Mother_to_child_groupB_Risk$Sample.size))

Mother_to_child_groupB_Risk$Events<- Mother_to_child_groupB_Risk$Sample.size*
Mother_to_child_groupB_Risk$Num_Estimate

Mother_to_child_groupB_Risk$Events_integer<-round( Mother_to_child_groupB_Risk$Events)

Mother_to_child_groupB_Risk$No_Event<- Mother_to_child_groupB_Risk$Sample.size-
Mother_to_child_groupB_Risk$Events_integer


model.mother.to.child.risk.GB<-
glmer(Events_integer/Sample.size~1+(1|id),weights=Sample.size,data=
Mother_to_child_groupB_Risk,family="binomial")

se.logit.mother.to.child.GB<-sqrt(vcov(model.mother.to.child.risk.GB))


################################################################ Occupational Exposure

#. E. coli

Occupational_Exposure_E.coli_OR_poultry<-subset(Occupational_Exposure,
Occupational_Exposure$GroupGood=="E.Coli" &
Occupational_Exposure$Method.of.estimation=="OR"
&Occupational_Exposure$Animal.involved.in.route=="poultry")
```

```
Occupational_Exposure_E.coli_OR_poultry$logOR<-
log(Occupational_Exposure_E.coli_OR_poultry$Num_Estimate)

Occupational_Exposure_E.coli_OR_poultry$loglb<-
log(Occupational_Exposure_E.coli_OR_poultry$Num_lb)

Occupational_Exposure_E.coli_OR_poultry$logub<-
log(Occupational_Exposure_E.coli_OR_poultry$Num_ub)

Occupational_Exposure_E.coli_OR_poultry$diflogCI<-
Occupational_Exposure_E.coli_OR_poultry$logub-Occupational_Exposure_E.coli_OR_poultry$loglb

Occupational_Exposure_E.coli_OR_poultry$logSE<-
Occupational_Exposure_E.coli_OR_poultry$diflogCI/3.92


or.occu.exp.poultry<-rma(yi = (Occupational_Exposure_E.coli_OR_poultry$logOR) , sei =
(Occupational_Exposure_E.coli_OR_poultry$logSE), method = "ML",measure = "RR")

summary(or.occu.exp.poultry)


Occupational_Exposure_E.coli_PR<-subset(Occupational_Exposure,
Occupational_Exposure$GroupGood=="E.Coli" &
Occupational_Exposure$Method.of.estimation=="PR")

Occupational_Exposure_E.coli_PR<-Occupational_Exposure_E.coli_PR[c(1,2),]


Occupational_Exposure_E.coli_Risk<-subset(Occupational_Exposure,
Occupational_Exposure$GroupGood=="E.Coli" &
Occupational_Exposure$Method.of.estimation=="Risk")

#uninfected and infected are from the same studiy as the other poultry, they contain the same
people so cannot be pooled

Occupational_Exposure_E.coli_Genes<-subset(Occupational_Exposure,
Occupational_Exposure$GroupGood=="E.Coli" &
Occupational_Exposure$Method.of.estimation=="Genes")

#Risk & Genes cannot be pooled due to different animals/occupation exposures


# Enterobacteriae

Occupational_Exposure_Entero_OR<-subset(Occupational_Exposure,
Occupational_Exposure$GroupGood=="Enterobacteriaceae(multiple or unspecified)" &
Occupational_Exposure$Method.of.estimation=="OR")
```

#The meta-analysis below could no longer be performed. The study of Dohmen has a reference category that also has occupational exposure to pigs. This article is mentioned in the review as more hours indicate more colonization.

```
#Occupational_Exposure_Entero_OR_pig<-Occupational_Exposure_Entero_OR[c(1,2),]

#Occupational_Exposure_Entero_OR_pig$logOR<-
log(Occupational_Exposure_Entero_OR_pig$Num_Estimate)

#Occupational_Exposure_Entero_OR_pig$loglb<-
log(Occupational_Exposure_Entero_OR_pig$Num_lb)

#Occupational_Exposure_Entero_OR_pig$logub<-
log(Occupational_Exposure_Entero_OR_pig$Num_ub)

#Occupational_Exposure_Entero_OR_pig$diflogCI<- Occupational_Exposure_Entero_OR_pig$logub-
Occupational_Exposure_Entero_OR_pig$loglb

#Occupational_Exposure_Entero_OR_pig$logSE<-
Occupational_Exposure_Entero_OR_pig$diflogCI/3.92


#or.occu.exp.pig.entero<-rma(yi = (Occupational_Exposure_Entero_OR_pig$logOR) , sei =
(Occupational_Exposure_Entero_OR_pig$logSE), method = "ML",measure = "RR")

#summary(or.occu.exp.pig.entero)


Occupational_Exposure_Entero_Risk<-subset(Occupational_Exposure,
Occupational_Exposure$GroupGood=="Enterobacteriaceae(multiple or unspecified)" &
Occupational_Exposure$Method.of.estimation=="Risk")

Occupational_Exposure_Entero_Risk_pig<-Occupational_Exposure_Entero_Risk[c(1:3),]


Occupational_Exposure_Entero_Risk_pig$id<-c(1:3)

Occupational_Exposure_Entero_Risk_pig$Num_Estimate<- (as.numeric(gsub("[\\%,]","",
Occupational_Exposure_Entero_Risk_pig$Estimate)))/100

Occupational_Exposure_Entero_Risk_pig$Sample.size<-as.numeric(as.character(
Occupational_Exposure_Entero_Risk_pig$Sample.size))

Occupational_Exposure_Entero_Risk_pig$Events<-
Occupational_Exposure_Entero_Risk_pig$Sample.size*
Occupational_Exposure_Entero_Risk_pig$Num_Estimate

Occupational_Exposure_Entero_Risk_pig$Events_integer<-round(
Occupational_Exposure_Entero_Risk_pig$Events)

Occupational_Exposure_Entero_Risk_pig$No_Event<-
Occupational_Exposure_Entero_Risk_pig$Sample.size-
Occupational_Exposure_Entero_Risk_pig$Events_integer
```

```
model.occu.exp.pig.entero<-glmer(Events_integer/Sample.size~1+(1|id),weights=Sample.size,data=
Occupational_Exposure_Entero_Risk_pig,family="binomial")

se.logit.occu.exp.pig.entero<-sqrt(vcov(model.occu.exp.pig.entero))


Occupational_Exposure_Entero_genes<-subset(Occupational_Exposure,
Occupational_Exposure$GroupGood=="Enterobacteriaceae(multiple or unspecified)" &
Occupational_Exposure$Method.of.estimation=="Genes")


# S. Aureus

#OR

Occupational_Exposure_SAureus_OR<-subset(Occupational_Exposure,
Occupational_Exposure$GroupGood=="S. Aureus" &
Occupational_Exposure$Method.of.estimation=="OR")

Occupational_Exposure_SAureus_OR_pig<-subset(Occupational_Exposure,
Occupational_Exposure$GroupGood=="S. Aureus" &
Occupational_Exposure$Method.of.estimation=="OR" &
Occupational_Exposure$Animal.involved.in.route=="pig")

Occupational_Exposure_SAureus_OR_pig<-Occupational_Exposure_SAureus_OR_pig[c(1:9,13:17),]
#10-12 are excluded because their reference category also has occupatoinal pig exposure


Occupational_Exposure_SAureus_OR_pig$logOR<-
log(Occupational_Exposure_SAureus_OR_pig$Num_Estimate)

Occupational_Exposure_SAureus_OR_pig$loglb<-
log(Occupational_Exposure_SAureus_OR_pig$Num_lb)

Occupational_Exposure_SAureus_OR_pig$logub<-
log(Occupational_Exposure_SAureus_OR_pig$Num_ub)

Occupational_Exposure_SAureus_OR_pig$diflogCI<-
Occupational_Exposure_SAureus_OR_pig$logub-Occupational_Exposure_SAureus_OR_pig$loglb

Occupational_Exposure_SAureus_OR_pig$logSE<-
Occupational_Exposure_SAureus_OR_pig$diflogCI/3.92


or.occu.exp.pig.saureus<-rma(yi = (Occupational_Exposure_SAureus_OR_pig$logOR) , sei =
(Occupational_Exposure_SAureus_OR_pig$logSE), method = "ML",measure = "RR")

summary(or.occu.exp.pig.saureus)
```

```
Occupational_Exposure_SAureus_OR_cattle<-subset(Occupational_Exposure,
Occupational_Exposure$GroupGood=="S. Aureus" &
Occupational_Exposure$Method.of.estimation=="OR" &
Occupational_Exposure$Animal.involved.in.route=="catle")


Occupational_Exposure_SAureus_OR_cattle$logOR<-
log(Occupational_Exposure_SAureus_OR_cattle$Num_Estimate)

Occupational_Exposure_SAureus_OR_cattle$loglb<-
log(Occupational_Exposure_SAureus_OR_cattle$Num_lb)

Occupational_Exposure_SAureus_OR_cattle$logub<-
log(Occupational_Exposure_SAureus_OR_cattle$Num_ub)

Occupational_Exposure_SAureus_OR_cattle$diflogCI<-
Occupational_Exposure_SAureus_OR_cattle$logub-
Occupational_Exposure_SAureus_OR_cattle$loglb

Occupational_Exposure_SAureus_OR_cattle$logSE<-
Occupational_Exposure_SAureus_OR_cattle$diflogCI/3.92


or.occu.exp.cattle.saureus<-rma(yi = (Occupational_Exposure_SAureus_OR_cattle$logOR) , sei =
(Occupational_Exposure_SAureus_OR_cattle$logSE), method = "ML",measure = "RR")

summary(or.occu.exp.cattle.saureus)


#No longer included because reference group also has occupational exposure to poultry

#Occupational_Exposure_SAureus_OR_poultry<-subset(Occupational_Exposure,
Occupational_Exposure$GroupGood=="S. Aureus" &
Occupational_Exposure$Method.of.estimation=="OR" &
Occupational_Exposure$Animal.involved.in.route=="poultry")


#Occupational_Exposure_SAureus_OR_poultry$logOR<-
log(Occupational_Exposure_SAureus_OR_poultry$Num_Estimate)

#Occupational_Exposure_SAureus_OR_poultry$loglb<-
log(Occupational_Exposure_SAureus_OR_poultry$Num_lb)

#Occupational_Exposure_SAureus_OR_poultry$logub<-
log(Occupational_Exposure_SAureus_OR_poultry$Num_ub)

#Occupational_Exposure_SAureus_OR_poultry$diflogCI<-
Occupational_Exposure_SAureus_OR_poultry$logub-
Occupational_Exposure_SAureus_OR_poultry$loglb

#Occupational_Exposure_SAureus_OR_poultry$logSE<-
Occupational_Exposure_SAureus_OR_poultry$diflogCI/3.92
```

```
#or.occu.exp.poultry.saureus<-rma(yi = (Occupational_Exposure_SAureus_OR_poultry$logOR) , sei = (Occupational_Exposure_SAureus_OR_poultry$logSE), method = "ML",measure = "RR")

#summary(or.occu.exp.poultry.saureus)


Occupational_Exposure_SAureus_OR_vets<-Occupational_Exposure_SAureus_OR[c(8,9,22,23),]


Occupational_Exposure_SAureus_OR_vets$logOR<-log(Occupational_Exposure_SAureus_OR_vets$Num_Estimate)

Occupational_Exposure_SAureus_OR_vets$loglb<-log(Occupational_Exposure_SAureus_OR_vets$Num_lb)

Occupational_Exposure_SAureus_OR_vets$logub<-log(Occupational_Exposure_SAureus_OR_vets$Num_ub)

Occupational_Exposure_SAureus_OR_vets$diflogCI<-Occupational_Exposure_SAureus_OR_vets$logub-Occupational_Exposure_SAureus_OR_vets$loglb

Occupational_Exposure_SAureus_OR_vets$logSE<-Occupational_Exposure_SAureus_OR_vets$diflogCI/3.92


or.occu.exp.vets.sarues<-rma(yi = (Occupational_Exposure_SAureus_OR_vets$logOR) , sei = (Occupational_Exposure_SAureus_OR_vets$logSE), method = "ML",measure = "RR")

summary(or.occu.exp.poultry)


Occupational_Exposure_SAureus_Risk<-subset(Occupational_Exposure, Occupational_Exposure$GroupGood=="S. Aureus" & Occupational_Exposure$Method.of.estimation=="Risk")


#PR

Occupational_Exposure_SAureus_PR<-subset(Occupational_Exposure, Occupational_Exposure$GroupGood=="S. Aureus" & Occupational_Exposure$Method.of.estimation=="PR")

Occupational_Exposure_SAureus_PR_poultry<-subset(Occupational_Exposure, Occupational_Exposure$GroupGood=="S. Aureus" & Occupational_Exposure$Method.of.estimation=="PR" & Occupational_Exposure$Animal.involved.in.route=="poultry")
```

```
Occupational_Exposure_SAureus_PR_poultry$logOR<-
log(Occupational_Exposure_SAureus_PR_poultry$Num_Estimate)

Occupational_Exposure_SAureus_PR_poultry$loglb<-
log(Occupational_Exposure_SAureus_PR_poultry$Num_lb)

Occupational_Exposure_SAureus_PR_poultry$logub<-
log(Occupational_Exposure_SAureus_PR_poultry$Num_ub)

Occupational_Exposure_SAureus_PR_poultry$diflogCI<-
Occupational_Exposure_SAureus_PR_poultry$logub-
Occupational_Exposure_SAureus_PR_poultry$loglb

Occupational_Exposure_SAureus_PR_poultry$logSE<-
Occupational_Exposure_SAureus_PR_poultry$diflogCI/3.92


pr.occu.exp.saureus.poultry<-rma(yi = (Occupational_Exposure_SAureus_PR_poultry$logOR) , sei =
(Occupational_Exposure_SAureus_PR_poultry$logSE), method = "ML",measure = "RR")

summary(pr.occu.exp.saureus.poultry)


Occupational_Exposure_SAureus_PR_Pig<-subset(Occupational_Exposure,
Occupational_Exposure$GroupGood=="S. Aureus" &
Occupational_Exposure$Method.of.estimation=="PR" &
Occupational_Exposure$Animal.involved.in.route=="pig"
|Occupational_Exposure$Animal.involved.in.route=="hog"|Occupational_Exposure$Animal.involved
.in.route=="swine")


Occupational_Exposure_SAureus_PR_Pig$logOR<-
log(Occupational_Exposure_SAureus_PR_Pig$Num_Estimate)

Occupational_Exposure_SAureus_PR_Pig$loglb<-
log(Occupational_Exposure_SAureus_PR_Pig$Num_lb)

Occupational_Exposure_SAureus_PR_Pig$logub<-
log(Occupational_Exposure_SAureus_PR_Pig$Num_ub)

Occupational_Exposure_SAureus_PR_Pig$diflogCI<-
Occupational_Exposure_SAureus_PR_Pig$logub-Occupational_Exposure_SAureus_PR_Pig$loglb

Occupational_Exposure_SAureus_PR_Pig$logSE<-
Occupational_Exposure_SAureus_PR_Pig$diflogCI/3.92


pr.occu.exp.saureus.pig<-rma(yi = (Occupational_Exposure_SAureus_PR_Pig$logOR) , sei =
(Occupational_Exposure_SAureus_PR_Pig$logSE), method = "ML",measure = "RR")

summary(pr.occu.exp.saureus.pig)
```

```
#Risk

Occupational_Exposure_SAureus_Risk<-subset(Occupational_Exposure,
Occupational_Exposure$GroupGood=="S. Aureus" &
Occupational_Exposure$Method.of.estimation=="Risk")

Occupational_Exposure_SAureus_Risk$Animal.involved.in.route[17]<-"pig"

Occupational_Exposure_SAureus_Risk_pig<-subset(Occupational_Exposure_SAureus_Risk,
Occupational_Exposure_SAureus_Risk$Animal.involved.in.route=="pig")


Occupational_Exposure_SAureus_Risk_pig<-
Occupational_Exposure_SAureus_Risk_pig[!(Occupational_Exposure_SAureus_Risk_pig$Author=="
Witte"),]

# The study by Witte et al is not included in the meta-analysis as the method of risk assessment was different then the other studies, these were collonise farmers which were then asked it they saw pigs


Occupational_Exposure_SAureus_Risk_pig$id<-c(1:9)

Occupational_Exposure_SAureus_Risk_pig$Num_Estimate<- (as.numeric(gsub("[\\%,]","",
Occupational_Exposure_SAureus_Risk_pig$Estimate)))/100

Occupational_Exposure_SAureus_Risk_pig$Sample.size<-as.numeric(as.character(
Occupational_Exposure_SAureus_Risk_pig$Sample.size))

Occupational_Exposure_SAureus_Risk_pig$Events<-
Occupational_Exposure_SAureus_Risk_pig$Sample.size*
Occupational_Exposure_SAureus_Risk_pig$Num_Estimate

Occupational_Exposure_SAureus_Risk_pig$Events_integer<-round(
Occupational_Exposure_SAureus_Risk_pig$Events)

Occupational_Exposure_SAureus_Risk_pig$No_Event<-
Occupational_Exposure_SAureus_Risk_pig$Sample.size-
Occupational_Exposure_SAureus_Risk_pig$Events_integer


model.occu.exp.pig.saureus<-
glmer(Events_integer/Sample.size~1+(1|id),weights=Sample.size,data=
Occupational_Exposure_SAureus_Risk_pig,family="binomial")

se.logit.occu.exp.pig.saureus<-sqrt(vcov(model.occu.exp.pig.saureus))


Occupational_Exposure_SAureus_Risk_vet<-Occupational_Exposure_SAureus_Risk[c(8,10:13),]
```

```
Occupational_Exposure_SAureus_Risk_vet$id<-c(1:5)

Occupational_Exposure_SAureus_Risk_vet$Num_Estimate<- (as.numeric(gsub("[\\%,]","",
Occupational_Exposure_SAureus_Risk_vet$Estimate)))/100

Occupational_Exposure_SAureus_Risk_vet$Sample.size<-as.numeric(as.character(
Occupational_Exposure_SAureus_Risk_vet$Sample.size))

Occupational_Exposure_SAureus_Risk_vet$Events<-
Occupational_Exposure_SAureus_Risk_vet$Sample.size*
Occupational_Exposure_SAureus_Risk_vet$Num_Estimate

Occupational_Exposure_SAureus_Risk_vet$Events_integer<-round(
Occupational_Exposure_SAureus_Risk_vet$Events)

Occupational_Exposure_SAureus_Risk_vet$No_Event<-
Occupational_Exposure_SAureus_Risk_vet$Sample.size-
Occupational_Exposure_SAureus_Risk_vet$Events_integer


model.occu.exp.vet.risk<-glmer(Events_integer/Sample.size~1+(1|id),weights=Sample.size,data=
Occupational_Exposure_SAureus_Risk_vet,family="binomial")

se.logit.occu.exp.vet.risk<-sqrt(vcov(model.occu.exp.vet.risk))

#Genes

Occupational_Exposure_SAureus_Genes<-subset(Occupational_Exposure,
Occupational_Exposure$GroupGood=="S. Aureus" &
Occupational_Exposure$Method.of.estimation=="Genes")

Occupational_Exposure_SAureus_Genes_pig<-subset(Occupational_Exposure,
Occupational_Exposure$GroupGood=="S. Aureus" &
Occupational_Exposure$Method.of.estimation=="Genes" &
Occupational_Exposure$Animal.involved.in.route=="pig")

Occupational_Exposure_SAureus_Genes_pig$id<-c(1:4)

Occupational_Exposure_SAureus_Genes_pig$Num_Estimate<- (as.numeric(gsub("[\\%,]","",
Occupational_Exposure_SAureus_Genes_pig$Estimate)))/100

Occupational_Exposure_SAureus_Genes_pig$Sample.size<-as.numeric(as.character(
Occupational_Exposure_SAureus_Genes_pig$Sample.size))

Occupational_Exposure_SAureus_Genes_pig$Events<-
Occupational_Exposure_SAureus_Genes_pig$Sample.size*
Occupational_Exposure_SAureus_Genes_pig$Num_Estimate

Occupational_Exposure_SAureus_Genes_pig$Events_integer<-round(
Occupational_Exposure_SAureus_Genes_pig$Events)

Occupational_Exposure_SAureus_Genes_pig$No_Event<-
Occupational_Exposure_SAureus_Genes_pig$Sample.size-
Occupational_Exposure_SAureus_Genes_pig$Events_integer
```

```
model.occu.exp.genes.saureus.pig<-
glmer(Events_integer/Sample.size~1+(1|id),weights=Sample.size,data=
Occupational_Exposure_SAureus_Genes_pig,family="binomial")

se.logit.occu.exp.genes.saureus.pig<-sqrt(vcov(model.occu.exp.genes.saureus.pig))


# VRE

Occupational_Exposure_VRE<-subset(Occupational_Exposure,
Occupational_Exposure$GroupGood=="VRE")


#Staphylococci

Occupational_Exposure_Staph<-subset(Occupational_Exposure,
Occupational_Exposure$GroupGood=="Staphylococci")

Occupational_Exposure_Staph_pig_risk<-subset(Occupational_Exposure,
Occupational_Exposure$GroupGood=="Staphylococci" &
Occupational_Exposure$Method.of.estimation=="Risk" &
Occupational_Exposure$Animal.involved.in.route=="pig")

Occupational_Exposure_Staph_pig_risk$id<-c(1:3)

Occupational_Exposure_Staph_pig_risk$Num_Estimate<- (as.numeric(gsub("[\\%,]","",
Occupational_Exposure_Staph_pig_risk$Estimate)))/100

Occupational_Exposure_Staph_pig_risk$Sample.size<-as.numeric(as.character(
Occupational_Exposure_Staph_pig_risk$Sample.size))

Occupational_Exposure_Staph_pig_risk$Events<-
Occupational_Exposure_Staph_pig_risk$Sample.size*
Occupational_Exposure_Staph_pig_risk$Num_Estimate

Occupational_Exposure_Staph_pig_risk$Events_integer<-round(
Occupational_Exposure_Staph_pig_risk$Events)

Occupational_Exposure_Staph_pig_risk$No_Event<-
Occupational_Exposure_Staph_pig_risk$Sample.size-
Occupational_Exposure_Staph_pig_risk$Events_integer


model.occu.exp.staph.risk.pig<-
glmer(Events_integer/Sample.size~1+(1|id),weights=Sample.size,data=
Occupational_Exposure_Staph_pig_risk,family="binomial")

se.logit.occu.exp.staph.risk.pig<-sqrt(vcov(model.occu.exp.staph.risk.pig))
```

```
Occupational_Exposure_Staph_pig_PR<-subset(Occupational_Exposure,
Occupational_Exposure$GroupGood=="Staphylococci"&
Occupational_Exposure$Method.of.estimation=="PR" &
Occupational_Exposure$Animal.involved.in.route=="pig")


# This one is no longer included as the reference group also has occupational exposure and then only 1 study remains

#Occupational_Exposure_Staph_pig_PR$logOR<-
log(Occupational_Exposure_Staph_pig_PR$Num_Estimate)

#Occupational_Exposure_Staph_pig_PR$loglb<-log(Occupational_Exposure_Staph_pig_PR$Num_lb)

#Occupational_Exposure_Staph_pig_PR$logub<-
log(Occupational_Exposure_Staph_pig_PR$Num_ub)

#Occupational_Exposure_Staph_pig_PR$diflogCI<- Occupational_Exposure_Staph_pig_PR$logub-
Occupational_Exposure_Staph_pig_PR$loglb

#Occupational_Exposure_Staph_pig_PR$logSE<-
Occupational_Exposure_Staph_pig_PR$diflogCI/3.92


#pr.occu.exp.staph.pig<-rma(yi = (Occupational_Exposure_Staph_pig_PR$logOR) , sei =
(Occupational_Exposure_Staph_pig_PR$logSE), method = "ML",measure = "RR")

#summary(pr.occu.exp.staph.pig)


#epidermis


Occupational_Exposure_epi<-subset(Occupational_Exposure,
Occupational_Exposure$GroupGood=="Staphylococcus epidermidis")

# no meta. time frame too different

#homolyticus

Occupational_Exposure_haemo_PR_pig<-subset(Occupational_Exposure,
Occupational_Exposure$GroupGood=="Staphylococcus haemolyticus")

# no meta. time frame too different


############################################################ Pets to humans

Pet_to_human_ecoli_OR<-subset(Pet_to_human, Pet_to_human$GroupGood=="E.Coli"
&Pet_to_human$Method.of.estimation=="OR")

Pet_to_human_ecoli_OR$logOR<-log(Pet_to_human_ecoli_OR$Num_Estimate)
```

```
Pet_to_human_ecoli_OR$loglb<-log(Pet_to_human_ecoli_OR$Num_lb)

Pet_to_human_ecoli_OR$logub<-log(Pet_to_human_ecoli_OR$Num_ub)

Pet_to_human_ecoli_OR$diflogCI<- Pet_to_human_ecoli_OR$logub-Pet_to_human_ecoli_OR$loglb

Pet_to_human_ecoli_OR$logSE<-Pet_to_human_ecoli_OR$diflogCI/3.92


OR.pet.tp.human.ecoli<-rma(yi = (Pet_to_human_ecoli_OR$logOR) , sei =
(Pet_to_human_ecoli_OR$logSE), method = "ML",measure = "OR")

summary(OR.pet.tp.human.ecoli)


Pet_to_human_entero_OR<-subset(Pet_to_human,
Pet_to_human$GroupGood=="Enterobacteriaceae(multiple or unspecified)"
&Pet_to_human$Method.of.estimation=="OR")

Pet_to_human_entero_OR$logOR<-log(Pet_to_human_entero_OR$Num_Estimate)

Pet_to_human_entero_OR$loglb<-log(Pet_to_human_entero_OR$Num_lb)

Pet_to_human_entero_OR$logub<-log(Pet_to_human_entero_OR$Num_ub)

Pet_to_human_entero_OR$diflogCI<- Pet_to_human_entero_OR$logub-
Pet_to_human_entero_OR$loglb

Pet_to_human_entero_OR$logSE<-Pet_to_human_entero_OR$diflogCI/3.92


#OR.pet.tp.human.entero<-rma(yi = (Pet_to_human_entero_OR$logOR) , sei =
(Pet_to_human_entero_OR$logSE), method = "ML",measure = "OR")

#summary(OR.pet.tp.human.entero)

#no similar time span


Pet_to_human_staph_OR<-subset(Pet_to_human, Pet_to_human$GroupGood=="Staphylococci"
&Pet_to_human$Method.of.estimation=="OR")

Pet_to_human_staph_OR$logOR<-log(Pet_to_human_staph_OR$Num_Estimate)

Pet_to_human_staph_OR$loglb<-log(Pet_to_human_staph_OR$Num_lb)

Pet_to_human_staph_OR$logub<-log(Pet_to_human_staph_OR$Num_ub)

Pet_to_human_staph_OR$diflogCI<- Pet_to_human_staph_OR$logub-
Pet_to_human_staph_OR$loglb

Pet_to_human_staph_OR$logSE<-Pet_to_human_staph_OR$diflogCI/3.92
```

```
OR.pet.tp.human.staph<-rma(yi = (Pet_to_human_staph_OR$logOR) , sei =
(Pet_to_human_staph_OR$logSE), method = "ML",measure = "OR")

summary(OR.pet.tp.human.staph)


#S Areus

Risk_Pet_to_human_saureus<-subset(Pet_to_human, Pet_to_human$GroupGood=="S. Aureus" &
Pet_to_human$Method.of.estimation=="Risk")

Genes_Pet_to_human_saureus<-subset(Pet_to_human, Pet_to_human$GroupGood=="S. Aureus" &
Pet_to_human$Method.of.estimation=="Genes")

Risk_Pet_to_human_saureus<-Risk_Pet_to_human_saureus[c(1:2,5),]

Risk_Pet_to_human_saureus$id<-c(1:3)

Risk_Pet_to_human_saureus$Num_Estimate<- (as.numeric(gsub("[\\%,]","",
Risk_Pet_to_human_saureus$Estimate)))/100

Risk_Pet_to_human_saureus$Sample.size<-as.numeric(as.character(
Risk_Pet_to_human_saureus$Sample.size))

Risk_Pet_to_human_saureus$Events<- Risk_Pet_to_human_saureus$Sample.size*
Risk_Pet_to_human_saureus$Num_Estimate

Risk_Pet_to_human_saureus$Events_integer<-round( Risk_Pet_to_human_saureus$Events)

Risk_Pet_to_human_saureus$No_Event<- Risk_Pet_to_human_saureus$Sample.size-
Risk_Pet_to_human_saureus$Events_integer


model.risk.pethuman.saureus<-
glmer(Events_integer/Sample.size~1+(1|id),weights=Sample.size,data=
Risk_Pet_to_human_saureus,family="binomial")

se.logit.risk.pethuman.saureus<-sqrt(vcov(model.risk.pethuman.saureus))


Genes_Pet_to_human_saureus_horse<-Genes_Pet_to_human_saureus[c(2,5,9),] # Eventually
decided that these are two different two use in paper

Genes_Pet_to_human_saureus<-Genes_Pet_to_human_saureus[c(1,3,4,6,8,10),] # one estimate is
on % of transmission, so I will leave that one out


Genes_Pet_to_human_saureus<-Genes_Pet_to_human_saureus[c(2:6),]
```

```
Genes_Pet_to_human_saureus$id<-c(1:5)

Genes_Pet_to_human_saureus$Num_Estimate<- (as.numeric(gsub("[\\%,]","",
Genes_Pet_to_human_saureus$Estimate)))/100

Genes_Pet_to_human_saureus$Sample.size<-as.numeric(as.character(
Genes_Pet_to_human_saureus$Sample.size))

Genes_Pet_to_human_saureus$Events<- Genes_Pet_to_human_saureus$Sample.size*
Genes_Pet_to_human_saureus$Num_Estimate

Genes_Pet_to_human_saureus$Events_integer<-round( Genes_Pet_to_human_saureus$Events)

Genes_Pet_to_human_saureus$No_Event<- Genes_Pet_to_human_saureus$Sample.size-
Genes_Pet_to_human_saureus$Events_integer


modelGenes_Pet_to_human_saureus<-
glmer(Events_integer/Sample.size~1+(1|id),weights=Sample.size,data=
Genes_Pet_to_human_saureus,family="binomial")

se.logitGenes_Pet_to_human_saureus<-sqrt(vcov(modelGenes_Pet_to_human_saureus))


kiekje<-subset(Transmission, Transmission$GroupGood=="Other")

Genes_Pet_to_human_saureus_horse$id<-c(1:3)

Genes_Pet_to_human_saureus_horse$Num_Estimate<- (as.numeric(gsub("[\\%,]","",
Genes_Pet_to_human_saureus_horse$Estimate)))/100

Genes_Pet_to_human_saureus_horse$Sample.size<-as.numeric(as.character(
Genes_Pet_to_human_saureus_horse$Sample.size))

Genes_Pet_to_human_saureus_horse$Events<-
Genes_Pet_to_human_saureus_horse$Sample.size*
Genes_Pet_to_human_saureus_horse$Num_Estimate

Genes_Pet_to_human_saureus_horse$Events_integer<-round(
Genes_Pet_to_human_saureus_horse$Events)

Genes_Pet_to_human_saureus_horse$No_Event<-
Genes_Pet_to_human_saureus_horse$Sample.size-
Genes_Pet_to_human_saureus_horse$Events_integer


modelGenes_Pet_to_human_saureus_horse<-
glmer(Events_integer/Sample.size~1+(1|id),weights=Sample.size,data=
Genes_Pet_to_human_saureus_horse,family="binomial")

se.logitGenes_Pet_to_human_saureus_horse<-
sqrt(vcov(modelGenes_Pet_to_human_saureus_horse))
```

```
############################################################ Prior colonised patient in room

Prior_col_patient_abau<-subset(Prior_col_patient, Prior_col_patient$GroupGood=="Acinetobacter baumannii")

Prior_col_patient_pseu<-subset(Prior_col_patient, Prior_col_patient$GroupGood=="Pseudomonas aeruginosa")

# Do not pool, seems like the same study just published elsewhere


############################################################ Contaminated room

Contaminated_room_VRE<-subset(Contaminated_room, Contaminated_room$GroupGood=="VRE")


############################################################ Space sharing

Sharing_room<-Space_sharing[c(2,3,5,6,7,11),]

Sharing_room_entero_OR<-subset(Sharing_room, Sharing_room$GroupGood=="Enterobacteriaceae(multiple or unspecified)"&Sharing_room$Method.of.estimation=="OR")


Sharing_room_entero_OR$logOR<-log(Sharing_room_entero_OR$Num_Estimate)

Sharing_room_entero_OR$loglb<-log(Sharing_room_entero_OR$Num_lb)

Sharing_room_entero_OR$logub<-log(Sharing_room_entero_OR$Num_ub)

Sharing_room_entero_OR$diflogCI<- Sharing_room_entero_OR$logub-Sharing_room_entero_OR$loglb

Sharing_room_entero_OR$logSE<-Sharing_room_entero_OR$diflogCI/3.92


OR.sharingroom.or<-rma(yi = (Sharing_room_entero_OR$logOR) , sei = (Sharing_room_entero_OR$logSE), method = "ML",measure = "OR")

summary(OR.sharingroom.or)


########################################################### Travelling

# OR

  # e coli

Travelling_OR_Ecoli<-subset(Travelling, Travelling$Method.of.estimation=="OR" & Travelling$GroupGood=="E.Coli")
```

```
Travelling_OR_Ecoli_SA<-subset(Travelling_OR_Ecoli, Travelling_OR_Ecoli$Transmission.route=="To India" | Travelling_OR_Ecoli$Transmission.route=="India "|Travelling_OR_Ecoli$Transmission.route=="Afghanistan")


Travelling_OR_Ecoli_SA$logOR<-log(Travelling_OR_Ecoli_SA$Num_Estimate)

Travelling_OR_Ecoli_SA$loglb<-log(Travelling_OR_Ecoli_SA$Num_lb)

Travelling_OR_Ecoli_SA$logub<-log(Travelling_OR_Ecoli_SA$Num_ub)

Travelling_OR_Ecoli_SA$diflogCI<- Travelling_OR_Ecoli_SA$logub-Travelling_OR_Ecoli_SA$loglb

Travelling_OR_Ecoli_SA$logSE<-Travelling_OR_Ecoli_SA$diflogCI/3.92


OR.travel_SA_Ecoli<-rma(yi = (Travelling_OR_Ecoli_SA$logOR) , sei = (Travelling_OR_Ecoli_SA$logSE), method = "ML",measure = "OR")

summary(OR.travel_SA_Ecoli)


 # Entero

Travelling_OR_Entero<-subset(Travelling, Travelling$Method.of.estimation=="OR" & Travelling$GroupGood==            "Enterobacteriaceae(multiple or unspecified)")

Travelling_OR_Entero_SEA<-subset(Travelling_OR_Entero, Travelling_OR_Entero$Transmission.route=="South East Asia last 3 months"|

                        Travelling_OR_Entero$Transmission.route=="South east Asia last 12 months"| Travelling_OR_Entero$Transmission.route=="Wester pacific last 3 months"|

                        Travelling_OR_Entero$Transmission.route=="Wester pacific last 12 months")

Travelling_OR_Entero_SEA$logOR<-log(Travelling_OR_Entero_SEA$Num_Estimate)

Travelling_OR_Entero_SEA$loglb<-log(Travelling_OR_Entero_SEA$Num_lb)

Travelling_OR_Entero_SEA$logub<-log(Travelling_OR_Entero_SEA$Num_ub)

Travelling_OR_Entero_SEA$diflogCI<- Travelling_OR_Entero_SEA$logub-Travelling_OR_Entero_SEA$loglb

Travelling_OR_Entero_SEA$logSE<-Travelling_OR_Entero_SEA$diflogCI/3.92

OR.travel_SEA_Entero<-rma(yi = (Travelling_OR_Entero_SEA$logOR) , sei = (Travelling_OR_Entero_SEA$logSE), method = "ML",measure = "OR")

summary(OR.travel_SEA_Entero)


Travelling_OR_Entero_SA<-subset(Travelling_OR_Entero, Travelling_OR_Entero$Transmission.route=="India"|
```

```
Travelling_OR_Entero$Transmission.route=="to India")

Travelling_OR_Entero_SA$logOR<-log(Travelling_OR_Entero_SA$Num_Estimate)
Travelling_OR_Entero_SA$loglb<-log(Travelling_OR_Entero_SA$Num_lb)
Travelling_OR_Entero_SA$logub<-log(Travelling_OR_Entero_SA$Num_ub)
Travelling_OR_Entero_SA$diflogCI<- Travelling_OR_Entero_SA$logub-
Travelling_OR_Entero_SA$loglb
Travelling_OR_Entero_SA$logSE<-Travelling_OR_Entero_SA$diflogCI/3.92
OR.travel_SA_Entero<-rma(yi = (Travelling_OR_Entero_SA$logOR) , sei =
(Travelling_OR_Entero_SA$logSE), method = "ML",measure = "OR")
summary(OR.travel_SA_Entero)

view(Travelling_OR_Entero_SA)
view(Travelling_OR_Entero_SEA)

Travelling_OR_Entero_America<-subset(Travelling_OR_Entero,
Travelling_OR_Entero$Transmission.route=="America last 3 months"|
Travelling_OR_Entero$Transmission.route=="America last 12 months")

#Travelling_OR_Entero_America$Num_ub[1]<-170.6387
#Travelling_OR_Entero_America$Num_ub[2]<-160.8023
Travelling_OR_Entero_America$logOR<-log(Travelling_OR_Entero_America$Num_Estimate)
Travelling_OR_Entero_America$loglb<-log(Travelling_OR_Entero_America$Num_lb)
Travelling_OR_Entero_America$logub<-log(Travelling_OR_Entero_America$Num_ub)
Travelling_OR_Entero_America$diflogCI<- Travelling_OR_Entero_America$logub-
Travelling_OR_Entero_America$loglb
Travelling_OR_Entero_America$logSE<-Travelling_OR_Entero_America$diflogCI/3.92
OR.travel_Amerika_enter<-rma(yi = (Travelling_OR_Entero_America$logOR) , sei =
(Travelling_OR_Entero_America$logSE), method = "ML",measure = "OR")
summary(OR.travel_Amerika_enter)

Travelling_OR_Entero_WA<-subset(Travelling_OR_Entero,
Travelling_OR_Entero$Transmission.route=="Eastern Mediterranean last 12 months"|
```

```
Travelling_OR_Entero$Transmission.route=="Eastern Mediterranean last 3 months"|
Travelling_OR_Entero$Transmission.route=="to Turkey")

Travelling_OR_Entero_WA$logOR<-log(Travelling_OR_Entero_WA$Num_Estimate)

Travelling_OR_Entero_WA$loglb<-log(Travelling_OR_Entero_WA$Num_lb)

Travelling_OR_Entero_WA$logub<-log(Travelling_OR_Entero_WA$Num_ub)

Travelling_OR_Entero_WA$diflogCI<- Travelling_OR_Entero_WA$logub-
Travelling_OR_Entero_WA$loglb

Travelling_OR_Entero_WA$logSE<-Travelling_OR_Entero_WA$diflogCI/3.92

OR.travel_WA_entero<-rma(yi = (Travelling_OR_Entero_WA$logOR) , sei =
(Travelling_OR_Entero_WA$logSE), method = "ML",measure = "OR")

summary(OR.travel_WA_entero)


Travelling_OR_Entero_Africa<-subset(Travelling_OR_Entero,
Travelling_OR_Entero$Transmission.route=="Africa last 3 months"|
Travelling_OR_Entero$Transmission.route=="Africa last 12 months"|
Travelling_OR_Entero$Transmission.route=="to Northern Africa" |
Travelling_OR_Entero$Transmission.route=="Africa north of equator")


Travelling_OR_Entero_Africa$logOR<-log(Travelling_OR_Entero_Africa$Num_Estimate)

Travelling_OR_Entero_Africa$loglb<-log(Travelling_OR_Entero_Africa$Num_lb)

Travelling_OR_Entero_Africa$logub<-log(Travelling_OR_Entero_Africa$Num_ub)

Travelling_OR_Entero_Africa$diflogCI<- Travelling_OR_Entero_Africa$logub-
Travelling_OR_Entero_Africa$loglb

Travelling_OR_Entero_Africa$logSE<-Travelling_OR_Entero_Africa$diflogCI/3.92

OR.travel_Africa_enter<-rma(yi = (Travelling_OR_Entero_Africa$logOR) , sei =
(Travelling_OR_Entero_Africa$logSE), method = "ML",measure = "OR")

summary(OR.travel_Africa_enter)


Travelling_OR_Entero_LatinAmerica<-subset(Travelling_OR_Entero,
Travelling_OR_Entero$Transmission.route=="Latin America")


Travelling_OR_Entero_LatinAmerica$logOR<-
log(Travelling_OR_Entero_LatinAmerica$Num_Estimate)

Travelling_OR_Entero_LatinAmerica$loglb<-log(Travelling_OR_Entero_LatinAmerica$Num_lb)

Travelling_OR_Entero_LatinAmerica$logub<-log(Travelling_OR_Entero_LatinAmerica$Num_ub)
```

```
Travelling_OR_Entero_LatinAmerica$diflogCI<- Travelling_OR_Entero_LatinAmerica$logub-
Travelling_OR_Entero_LatinAmerica$loglb

Travelling_OR_Entero_LatinAmerica$logSE<-Travelling_OR_Entero_LatinAmerica$diflogCI/3.92

OR.travel_LA_entero<-rma(yi = (Travelling_OR_Entero_LatinAmerica$logOR) , sei =
(Travelling_OR_Entero_LatinAmerica$logSE), method = "ML",measure = "OR")

summary(OR.travel_LA_entero)


Travelling_OR_Entero_Asia_unsp<-subset(Travelling_OR_Entero,
Travelling_OR_Entero$Transmission.route=="Asia
"|Travelling_OR_Entero$Transmission.route=="Asia (expect for India)")


Travelling_OR_Entero_Asia_unsp$logOR<-log(Travelling_OR_Entero_Asia_unsp$Num_Estimate)

Travelling_OR_Entero_Asia_unsp$loglb<-log(Travelling_OR_Entero_Asia_unsp$Num_lb)

Travelling_OR_Entero_Asia_unsp$logub<-log(Travelling_OR_Entero_Asia_unsp$Num_ub)

Travelling_OR_Entero_Asia_unsp$diflogCI<- Travelling_OR_Entero_Asia_unsp$logub-
Travelling_OR_Entero_Asia_unsp$loglb

Travelling_OR_Entero_Asia_unsp$logSE<-Travelling_OR_Entero_Asia_unsp$diflogCI/3.92

OR.travel_Asia_entero<-rma(yi = (Travelling_OR_Entero_Asia_unsp$logOR) , sei =
(Travelling_OR_Entero_Asia_unsp$logSE), method = "ML",measure = "OR")

summary(OR.travel_Asia_entero)


Travelling_OR_Entero_Europe<-subset(Travelling_OR_Entero,
Travelling_OR_Entero$Transmission.route=="Europe last 12
months"|Travelling_OR_Entero$Transmission.route=="Europe in last 3
months"|Travelling_OR_Entero$Transmission.route=="to Southern/Eastern Europe")


Travelling_OR_Entero_Europe$logOR<-log(Travelling_OR_Entero_Europe$Num_Estimate)

Travelling_OR_Entero_Europe$loglb<-log(Travelling_OR_Entero_Europe$Num_lb)

Travelling_OR_Entero_Europe$logub<-log(Travelling_OR_Entero_Europe$Num_ub)

Travelling_OR_Entero_Europe$diflogCI<- Travelling_OR_Entero_Europe$logub-
Travelling_OR_Entero_Europe$loglb

Travelling_OR_Entero_Europe$logSE<-Travelling_OR_Entero_Europe$diflogCI/3.92

OR.travel_Europe_entero<-rma(yi = (Travelling_OR_Entero_Europe$logOR) , sei =
(Travelling_OR_Entero_Europe$logSE), method = "ML",measure = "OR")

summary(OR.travel_Europe_entero)
```

```
# S aureus

Travelling_OR_Saereus<-subset(Travelling, Travelling$Method.of.estimation=="OR" & Travelling$GroupGood=="S. Aureus")

Travelling_OR_saures_South_asia<-subset(Travelling_OR_Saereus, Travelling_OR_Saereus$Transmission.route=="South Asia")


Travelling_OR_saures_South_asia$logOR<-log(Travelling_OR_saures_South_asia$Num_Estimate)

Travelling_OR_saures_South_asia$loglb<-log(Travelling_OR_saures_South_asia$Num_lb)

Travelling_OR_saures_South_asia$logub<-log(Travelling_OR_saures_South_asia$Num_ub)

Travelling_OR_saures_South_asia$diflogCI<- Travelling_OR_saures_South_asia$logub- Travelling_OR_saures_South_asia$loglb

Travelling_OR_saures_South_asia$logSE<-Travelling_OR_saures_South_asia$diflogCI/3.92


OR.travel_SA_SAreus<-rma(yi = (Travelling_OR_saures_South_asia$logOR) , sei = (Travelling_OR_saures_South_asia$logSE), method = "ML",measure = "OR")

summary(OR.travel_SA_SAreus)


# Risk

Travelling_Risk<-subset(Travelling, Travelling$Method.of.estimation=="Risk")

# ecoli

Travelling_Risk_ecoli<-subset(Travelling_Risk, Travelling_Risk$GroupGood=="E.Coli")

Travelling_Risk_ecoli_eu<-
subset(Travelling_Risk_ecoli,Travelling_Risk_ecoli$Transmission.route=="Northern Europe"|Travelling_Risk_ecoli$Transmission.route=="Eastern Europe" |Travelling_Risk_ecoli$Transmission.route=="Southern Europe" |Travelling_Risk_ecoli$Transmission.route=="Europe")

Travelling_Risk_ecoli_eu$id<-c(1:4)

Travelling_Risk_ecoli_eu$Num_Estimate<- (as.numeric(gsub("[\\%,]","", Travelling_Risk_ecoli_eu$Estimate)))/100

Travelling_Risk_ecoli_eu$Sample.size<-as.numeric(as.character( Travelling_Risk_ecoli_eu$Sample.size))

Travelling_Risk_ecoli_eu$Events<- Travelling_Risk_ecoli_eu$Sample.size* Travelling_Risk_ecoli_eu$Num_Estimate

Travelling_Risk_ecoli_eu$Events_integer<-round( Travelling_Risk_ecoli_eu$Events)
```

```
Travelling_Risk_ecoli_eu$No_Event<- Travelling_Risk_ecoli_eu$Sample.size-
Travelling_Risk_ecoli_eu$Events_integer


model.risk.travel.eu.ecoli<-glmer(Events_integer/Sample.size~1+(1|id),weights=Sample.size,data=
Travelling_Risk_ecoli_eu,family="binomial")

se.logit.risk.travel.eu.ecoli<-sqrt(vcov(model.risk.travel.eu.ecoli))


Travelling_Risk_ecoli_SA<-
subset(Travelling_Risk_ecoli,Travelling_Risk_ecoli$Transmission.route=="India"|Travelling_Risk_ecol
i$Transmission.route=="South Asia"|Travelling_Risk_ecoli$Transmission.route=="Afghanistan")

Travelling_Risk_ecoli_SA$id<-c(1:3)

Travelling_Risk_ecoli_SA$Num_Estimate<- (as.numeric(gsub("[\\%,]","",
Travelling_Risk_ecoli_SA$Estimate)))/100

Travelling_Risk_ecoli_SA$Sample.size<-as.numeric(as.character(
Travelling_Risk_ecoli_SA$Sample.size))

Travelling_Risk_ecoli_SA$Events<- Travelling_Risk_ecoli_SA$Sample.size*
Travelling_Risk_ecoli_SA$Num_Estimate

Travelling_Risk_ecoli_SA$Events_integer<-round( Travelling_Risk_ecoli_SA$Events)

Travelling_Risk_ecoli_SA$No_Event<- Travelling_Risk_ecoli_SA$Sample.size-
Travelling_Risk_ecoli_SA$Events_integer


model.risk.travel.SA.ecoli<-glmer(Events_integer/Sample.size~1+(1|id),weights=Sample.size,data=
Travelling_Risk_ecoli_SA,family="binomial")

se.logit.risk.travel.SA.ecoli<-sqrt(vcov(model.risk.travel.SA.ecoli))


Travelling_Risk_ecoli_LA<-
subset(Travelling_Risk_ecoli,Travelling_Risk_ecoli$Transmission.route=="Southern
America"|Travelling_Risk_ecoli$Transmission.route=="Mexico, Caribean & Central
america"|Travelling_Risk_ecoli$Transmission.route=="South America")

Travelling_Risk_ecoli_LA$id<-c(1:3)

Travelling_Risk_ecoli_LA$Num_Estimate<- (as.numeric(gsub("[\\%,]","",
Travelling_Risk_ecoli_LA$Estimate)))/100

Travelling_Risk_ecoli_LA$Sample.size<-as.numeric(as.character(
Travelling_Risk_ecoli_LA$Sample.size))

Travelling_Risk_ecoli_LA$Events<- Travelling_Risk_ecoli_LA$Sample.size*
Travelling_Risk_ecoli_LA$Num_Estimate
```

```
Travelling_Risk_ecoli_LA$Events_integer<-round( Travelling_Risk_ecoli_LA$Events)

Travelling_Risk_ecoli_LA$No_Event<- Travelling_Risk_ecoli_LA$Sample.size-
Travelling_Risk_ecoli_LA$Events_integer


model.risk.travel.ecoli.LA<-glmer(Events_integer/Sample.size~1+(1|id),weights=Sample.size,data=
Travelling_Risk_ecoli_LA,family="binomial")

se.logit.model.risk.travel.ecoli.LA<-sqrt(vcov(model.risk.travel.ecoli.LA))


Travelling_Risk_ecoli_Asia_un<-
subset(Travelling_Risk_ecoli,Travelling_Risk_ecoli$Transmission.route=="Asia")

Travelling_Risk_ecoli_Asia_un$id<-c(1:2)

Travelling_Risk_ecoli_Asia_un$Num_Estimate<- (as.numeric(gsub("[\\%,]","",
Travelling_Risk_ecoli_Asia_un$Estimate)))/100

Travelling_Risk_ecoli_Asia_un$Sample.size<-as.numeric(as.character(
Travelling_Risk_ecoli_Asia_un$Sample.size))

Travelling_Risk_ecoli_Asia_un$Events<- Travelling_Risk_ecoli_Asia_un$Sample.size*
Travelling_Risk_ecoli_Asia_un$Num_Estimate

Travelling_Risk_ecoli_Asia_un$Events_integer<-round( Travelling_Risk_ecoli_Asia_un$Events)

Travelling_Risk_ecoli_Asia_un$No_Event<- Travelling_Risk_ecoli_Asia_un$Sample.size-
Travelling_Risk_ecoli_Asia_un$Events_integer


model.risk.travel.asia.ecoli<-glmer(Events_integer/Sample.size~1+(1|id),weights=Sample.size,data=
Travelling_Risk_ecoli_Asia_un,family="binomial")

se.logit.risk.travel.asia.ecoli<-sqrt(vcov(model.risk.travel.asia.ecoli))


Travelling_Risk_ecoli_Africa<-
subset(Travelling_Risk_ecoli,Travelling_Risk_ecoli$Transmission.route=="Africa"|Travelling_Risk_ec
oli$Transmission.route=="Cote d'Ivoire" |Travelling_Risk_ecoli$Transmission.route=="French
Guiana")

Travelling_Risk_ecoli_Africa$id<-c(1:4)

Travelling_Risk_ecoli_Africa$Num_Estimate<- (as.numeric(gsub("[\\%,]","",
Travelling_Risk_ecoli_Africa$Estimate)))/100

Travelling_Risk_ecoli_Africa$Sample.size<-as.numeric(as.character(
Travelling_Risk_ecoli_Africa$Sample.size))

Travelling_Risk_ecoli_Africa$Events<- Travelling_Risk_ecoli_Africa$Sample.size*
Travelling_Risk_ecoli_Africa$Num_Estimate
```

```
Travelling_Risk_ecoli_Africa$Events_integer<-round( Travelling_Risk_ecoli_Africa$Events)

Travelling_Risk_ecoli_Africa$No_Event<- Travelling_Risk_ecoli_Africa$Sample.size-
Travelling_Risk_ecoli_Africa$Events_integer


model.risk.travel.africa.ecoli<-
glmer(Events_integer/Sample.size~1+(1|id),weights=Sample.size,data=
Travelling_Risk_ecoli_Africa,family="binomial")

se.logit.risk.travel.africa.ecoli<-sqrt(vcov(model.risk.travel.africa.ecoli))



#entero

Travelling_Risk_entero<-subset(Travelling_Risk,
Travelling_Risk$GroupGood=="Enterobacteriaceae(multiple or unspecified)")

Travelling_Risk_entero_africa<-subset(Travelling_Risk_entero,
Travelling_Risk_entero$Transmission.route=="Africa"|
Travelling_Risk_entero$Transmission.route=="North Africa"|
Travelling_Risk_entero$Transmission.route=="Central Africa"|

                    Travelling_Risk_entero$Transmission.route=="Southern Africa"|
Travelling_Risk_entero$Transmission.route=="Northern Africa"|
Travelling_Risk_entero$Transmission.route=="Middle and Eastern Africa"|
Travelling_Risk_entero$Transmission.route=="Western Africa"|


Travelling_Risk_entero$Transmission.route=="Africa"|Travelling_Risk_entero$Transmission.route==
"West and Central Africa"| Travelling_Risk_entero$Transmission.route=="East Africa")

Travelling_Risk_entero_africa$id<-c(1:12)

Travelling_Risk_entero_africa$Num_Estimate<- (as.numeric(gsub("[\\%,]","",
Travelling_Risk_entero_africa$Estimate)))/100

Travelling_Risk_entero_africa$Sample.size<-as.numeric(as.character(
Travelling_Risk_entero_africa$Sample.size))

Travelling_Risk_entero_africa$Events<- Travelling_Risk_entero_africa$Sample.size*
Travelling_Risk_entero_africa$Num_Estimate

Travelling_Risk_entero_africa$Events_integer<-round( Travelling_Risk_entero_africa$Events)

Travelling_Risk_entero_africa$No_Event<- Travelling_Risk_entero_africa$Sample.size-
Travelling_Risk_entero_africa$Events_integer
```

```
model.risk.travel.africa.entero<-
glmer(Events_integer/Sample.size~1+(1|id),weights=Sample.size,data=
Travelling_Risk_entero_africa,family="binomial")

se.logit.risk.travel.africa.entero<-sqrt(vcov(model.risk.travel.africa.entero))


Travelling_Risk_entero_LA<-subset(Travelling_Risk_entero,
Travelling_Risk_entero$Transmission.route=="Central America"|
Travelling_Risk_entero$Transmission.route=="South America"|
Travelling_Risk_entero$Transmission.route=="Central America and Caribbean"|

                                    Travelling_Risk_entero$Transmission.route=="Carribean and Central
America"| Travelling_Risk_entero$Transmission.route=="Latin America"|
Travelling_Risk_entero$Transmission.route=="Central America and the Caribbean")

Travelling_Risk_entero_LA$id<-c(1:9)

Travelling_Risk_entero_LA$Num_Estimate<- (as.numeric(gsub("[\\%,]","",
Travelling_Risk_entero_LA$Estimate)))/100

Travelling_Risk_entero_LA$Sample.size<-as.numeric(as.character(
Travelling_Risk_entero_LA$Sample.size))

Travelling_Risk_entero_LA$Events<- Travelling_Risk_entero_LA$Sample.size*
Travelling_Risk_entero_LA$Num_Estimate

Travelling_Risk_entero_LA$Events_integer<-round( Travelling_Risk_entero_LA$Events)

Travelling_Risk_entero_LA$No_Event<- Travelling_Risk_entero_LA$Sample.size-
Travelling_Risk_entero_LA$Events_integer


model.risk.travel.LA_enter<-glmer(Events_integer/Sample.size~1+(1|id),weights=Sample.size,data=
Travelling_Risk_entero_LA,family="binomial")

se.logit.risk.travel.LA_enter<-sqrt(vcov(model.risk.travel.LA_enter))


Travelling_Risk_entero_SA<-subset(Travelling_Risk_entero,
Travelling_Risk_entero$Transmission.route=="India"|
Travelling_Risk_entero$Transmission.route=="South Asia"|
Travelling_Risk_entero$Transmission.route=="Souther Asia")

Travelling_Risk_entero_SA$id<-c(1:5)

Travelling_Risk_entero_SA$Num_Estimate<- (as.numeric(gsub("[\\%,]","",
Travelling_Risk_entero_SA$Estimate)))/100

Travelling_Risk_entero_SA$Sample.size<-as.numeric(as.character(
Travelling_Risk_entero_SA$Sample.size))
```

```
Travelling_Risk_entero_SA$Events<- Travelling_Risk_entero_SA$Sample.size*
Travelling_Risk_entero_SA$Num_Estimate

Travelling_Risk_entero_SA$Events_integer<-round( Travelling_Risk_entero_SA$Events)

Travelling_Risk_entero_SA$No_Event<- Travelling_Risk_entero_SA$Sample.size-
Travelling_Risk_entero_SA$Events_integer


#view(Travelling_Risk_entero_SA)


model.risk.travel.SA.entero<-glmer(Events_integer/Sample.size~1+(1|id),weights=Sample.size,data=
Travelling_Risk_entero_SA,family="binomial")

se.logit.risk.travel.SA.entero<-sqrt(vcov(model.risk.travel.SA.entero))


Travelling_Risk_entero_WA<-subset(Travelling_Risk_entero,
Travelling_Risk_entero$Transmission.route=="Middle East"|
Travelling_Risk_entero$Transmission.route=="Western Asia")

Travelling_Risk_entero_WA$id<-c(1:3)

Travelling_Risk_entero_WA$Num_Estimate<- (as.numeric(gsub("[\\%,]","",
Travelling_Risk_entero_WA$Estimate)))/100

Travelling_Risk_entero_WA$Sample.size<-as.numeric(as.character(
Travelling_Risk_entero_WA$Sample.size))

Travelling_Risk_entero_WA$Events<- Travelling_Risk_entero_WA$Sample.size*
Travelling_Risk_entero_WA$Num_Estimate

Travelling_Risk_entero_WA$Events_integer<-round( Travelling_Risk_entero_WA$Events)

Travelling_Risk_entero_WA$No_Event<- Travelling_Risk_entero_WA$Sample.size-
Travelling_Risk_entero_WA$Events_integer


model.risk.travel.wa.entero<-
glmer(Events_integer/Sample.size~1+(1|id),weights=Sample.size,data=
Travelling_Risk_entero_WA,family="binomial")

se.logit.risk.travel.wa.entero<-sqrt(vcov(model.risk.travel.wa.entero))


Travelling_Risk_entero_SEA<-subset(Travelling_Risk_entero,
Travelling_Risk_entero$Transmission.route=="Southeast Asia"|
Travelling_Risk_entero$Transmission.route=="Southeastern Asia"|
Travelling_Risk_entero$Transmission.route=="South East Asia")

Travelling_Risk_entero_SEA$id<-c(1:3)
```

```
Travelling_Risk_entero_SEA$Num_Estimate<- (as.numeric(gsub("[\\%,]","",
Travelling_Risk_entero_SEA$Estimate)))/100

Travelling_Risk_entero_SEA$Sample.size<-as.numeric(as.character(
Travelling_Risk_entero_SEA$Sample.size))

Travelling_Risk_entero_SEA$Events<- Travelling_Risk_entero_SEA$Sample.size*
Travelling_Risk_entero_SEA$Num_Estimate

Travelling_Risk_entero_SEA$Events_integer<-round( Travelling_Risk_entero_SEA$Events)

Travelling_Risk_entero_SEA$No_Event<- Travelling_Risk_entero_SEA$Sample.size-
Travelling_Risk_entero_SEA$Events_integer


model.risk.travel.SEA.entero<-
glmer(Events_integer/Sample.size~1+(1|id),weights=Sample.size,data=
Travelling_Risk_entero_SEA,family="binomial")

se.logit.risk.travel.SEA.entero<-sqrt(vcov(model.risk.travel.SEA.entero))

#view(Travelling_Risk_entero_SEA)


Travelling_Risk_entero_EU<-subset(Travelling_Risk_entero,
Travelling_Risk_entero$Transmission.route=="Southern Europe")

Travelling_Risk_entero_EU$id<-c(1:2)

Travelling_Risk_entero_EU$Num_Estimate<- (as.numeric(gsub("[\\%,]","",
Travelling_Risk_entero_EU$Estimate)))/100

Travelling_Risk_entero_EU$Sample.size<-as.numeric(as.character(
Travelling_Risk_entero_EU$Sample.size))

Travelling_Risk_entero_EU$Events<- Travelling_Risk_entero_EU$Sample.size*
Travelling_Risk_entero_EU$Num_Estimate

Travelling_Risk_entero_EU$Events_integer<-round( Travelling_Risk_entero_EU$Events)

Travelling_Risk_entero_EU$No_Event<- Travelling_Risk_entero_EU$Sample.size-
Travelling_Risk_entero_EU$Events_integer


model.risk.travel.entero.eu<-glmer(Events_integer/Sample.size~1+(1|id),weights=Sample.size,data=
Travelling_Risk_entero_EU,family="binomial")

se.logit.risk.travel.entero.eu<-sqrt(vcov(model.risk.travel.entero.eu))


Travelling_Risk_entero_Asia_unspec<-subset(Travelling_Risk_entero,
Travelling_Risk_entero$Transmission.route=="Asia"|
```

```
Travelling_Risk_entero$Transmission.route=="Asia (India, Thailand, Laos, Cambodia, and Vietnam excluded)")

Travelling_Risk_entero_Asia_unspec$id<-c(1:3)

Travelling_Risk_entero_Asia_unspec$Num_Estimate<- (as.numeric(gsub("[\\%,]","",
Travelling_Risk_entero_Asia_unspec$Estimate)))/100

Travelling_Risk_entero_Asia_unspec$Sample.size<-as.numeric(as.character(
Travelling_Risk_entero_Asia_unspec$Sample.size))

Travelling_Risk_entero_Asia_unspec$Events<- Travelling_Risk_entero_Asia_unspec$Sample.size*
Travelling_Risk_entero_Asia_unspec$Num_Estimate

Travelling_Risk_entero_Asia_unspec$Events_integer<-round(
Travelling_Risk_entero_Asia_unspec$Events)

Travelling_Risk_entero_Asia_unspec$No_Event<- Travelling_Risk_entero_Asia_unspec$Sample.size-
Travelling_Risk_entero_Asia_unspec$Events_integer


model.risk.travel.asia.entero<-
glmer(Events_integer/Sample.size~1+(1|id),weights=Sample.size,data=
Travelling_Risk_entero_Asia_unspec,family="binomial")

se.logit.risk.travel.asia.entero<-sqrt(vcov(model.risk.travel.asia.entero))


Travelling_Risk_entero_CentralEastA<-subset(Travelling_Risk_entero,
Travelling_Risk_entero$Transmission.route=="Central Asia"|
Travelling_Risk_entero$Transmission.route=="Central and Eastern Asia"|
Travelling_Risk_entero$Transmission.route=="East Asia")

Travelling_Risk_entero_CentralEastA$id<-c(1:3)

Travelling_Risk_entero_CentralEastA$Num_Estimate<- (as.numeric(gsub("[\\%,]","",
Travelling_Risk_entero_CentralEastA$Estimate)))/100

Travelling_Risk_entero_CentralEastA$Sample.size<-as.numeric(as.character(
Travelling_Risk_entero_CentralEastA$Sample.size))

Travelling_Risk_entero_CentralEastA$Events<- Travelling_Risk_entero_CentralEastA$Sample.size*
Travelling_Risk_entero_CentralEastA$Num_Estimate

Travelling_Risk_entero_CentralEastA$Events_integer<-round(
Travelling_Risk_entero_CentralEastA$Events)

Travelling_Risk_entero_CentralEastA$No_Event<-
Travelling_Risk_entero_CentralEastA$Sample.size-
Travelling_Risk_entero_CentralEastA$Events_integer
```

```
model.risk.travel.centraleast.entero<-
glmer(Events_integer/Sample.size~1+(1|id),weights=Sample.size,data=
Travelling_Risk_entero_CentralEastA,family="binomial")

se.logit.risk.travel.centraleast.entero<-sqrt(vcov(model.risk.travel.centraleast.entero))


#PR

Travelling_PR<-subset(Travelling, Travelling$Method.of.estimation=="PR") #8, 1 entero multi de rest
1. coli

# non because unspecific multiple countries or only1 estimate


# Potential new extra meta-analysis based on >2 estimates


Animal_animal_risk_other<-subset(Animal_to_animal,
Animal_to_animal$Method.of.estimation=="Risk")# no to different

air_to_animal<-subset(Transmission, Transmission$Transmission.Group=="Air to animal")# no to
different

#Eating_meat # no to different

environement_to_human<-subset(Transmission, Transmission$Transmission.Group=='Environment -
> human')# no to different

food_to_animal<-subset(Transmission, Transmission$Transmission.Group=="Food-> animal")# no to
different

human_to_air<-subset(Transmission, Transmission$Transmission.Group=="Human-> Air")# no to
different

Interventions<-subset(Transmission, Transmission$Transmission.Group=="Intervention")# no to
different

motheranimal_to_baby<-subset(Transmission, Transmission$Transmission.Group=="Mother
(animal) -> child")

Transmission$Sample.size[667]<-13 # WEL

motheranimal_to_baby<-subset(Transmission, Transmission$Transmission.Group=="Mother
(animal) -> child")


Genes_motheranimal_to_baby<-subset(motheranimal_to_baby,
motheranimal_to_baby$Method.of.estimation=="Genes")

Genes_motheranimal_to_baby$id<-c(1:2)

Genes_motheranimal_to_baby$Num_Estimate<- (as.numeric(gsub("[\\%,]","",
Genes_motheranimal_to_baby$Estimate)))/100
```

```
Genes_motheranimal_to_baby$Sample.size<-as.numeric(as.character(
Genes_motheranimal_to_baby$Sample.size))

Genes_motheranimal_to_baby$Events<- Genes_motheranimal_to_baby$Sample.size*
Genes_motheranimal_to_baby$Num_Estimate

Genes_motheranimal_to_baby$Events_integer<-round( Genes_motheranimal_to_baby$Events)

Genes_motheranimal_to_baby$No_Event<- Genes_motheranimal_to_baby$Sample.size-
Genes_motheranimal_to_baby$Events_integer


modelGenes_motheranimal_to_baby<-
glmer(Events_integer/Sample.size~1+(1|id),weights=Sample.size,data=
Genes_motheranimal_to_baby,family="binomial")

se.logitGenes_motheranimal_to_baby<-sqrt(vcov(modelGenes_motheranimal_to_baby))


organtransfer_risk<-subset(Transmission, Transmission$Transmission.Group=="Organ") # yes, two
of 4 can be pooled for OR


Transmission$uni.of.multi[236]<-"uni"

Other_Bathing_Showering<-subset(Transmission, Transmission$Transmission.Group=="Other
Bathing/Showering")

Other_Bathing_Showering<-Other_Bathing_Showering[c(1:2),] # Wel


Other_Bathing_Showering$logOR<-log(Other_Bathing_Showering$Num_Estimate)

Other_Bathing_Showering$loglb<-log(Other_Bathing_Showering$Num_lb)

Other_Bathing_Showering$logub<-log(Other_Bathing_Showering$Num_ub)

Other_Bathing_Showering$diflogCI<- Other_Bathing_Showering$logub-
Other_Bathing_Showering$loglb

Other_Bathing_Showering$logSE<-Other_Bathing_Showering$diflogCI/3.92


OR.bathingshowering<-rma(yi = (Other_Bathing_Showering$logOR) , sei =
(Other_Bathing_Showering$logSE), method = "ML",measure = "OR")

summary(OR.bathingshowering) #1 is body use instead of hand, change here


Transmission$Sample.size<-as.numeric(as.character(paste(Transmission$Sample.size)))

Transmission$Sample.size[234]<-"232"
```

```
Transmission$Num_ub[234]<-"0.778"

Transmission$Num_lb[234]<-"0.118"

Transmission$Num_Estimate[234]<-"0.303"


Other_soap<-subset(Transmission, Transmission$Transmission.Group=="Other Soap")

Other_soap<-Other_soap[c(1:3),] # Yes


Other_soap$Num_Estimate<-as.numeric(Other_soap$Num_Estimate)

Other_soap$logOR<-log(Other_soap$Num_Estimate)

Other_soap$Num_lb<-as.numeric(Other_soap$Num_lb)

Other_soap$loglb<-log(Other_soap$Num_lb)

Other_soap$Num_ub<-as.numeric(Other_soap$Num_ub)

Other_soap$logub<-log(Other_soap$Num_ub)

Other_soap$diflogCI<- Other_soap$logub-Other_soap$loglb

Other_soap$logSE<-Other_soap$diflogCI/3.92


OR.soap<-rma(yi = (Other_soap$logOR) , sei = (Other_soap$logSE), method = "ML",measure = "OR")

summary(OR.soap) #1 is body use instead of hand, change here


veggies<-subset(Transmission, Transmission$Transmission.Group=="Vegetables-> human") # No

water_to_plant<-subset(Transmission, Transmission$Transmission.Group=="Water-> Plant") # No


# Cases per day meta-analysis

#view(Casesperday)


# Check time line occupational exposure

Occupational_exp_or<-subset(Occupational_Exposure,
Occupational_Exposure$Method.of.estimation=="OR")

Occupational_exp_risk<-subset(Occupational_Exposure,
Occupational_Exposure$Method.of.estimation=="Risk")

Occupational_exp_genes<-subset(Occupational_Exposure,
Occupational_Exposure$Method.of.estimation=="Genes")
```

```
Occupational_exp_PR<-subset(Occupational_Exposure, Occupational_Exposure$Method.of.estimation=="PR")


# Now we are gonna create a table of all meta analysis

# This code will not work if you deleted or added new meta analysis

# Then you will also have to add them in the way this table is created

########################################################## Table creation

TransmissionRoute<-c("Animal to Air", "Poultry to Air", "Pig to Air", "", "Cattle to Air", "Animal to Animal", "Pet to Pet","Pig to Pig", "Animal to Environment", "Poultry to Environment", "Pig to Environment", "Breast feeding",

                "", "", "", "", "Contact with an Infected Person", "","","","","","","","","","","","",

                "Eating meat to human", "White", "Red","General","Red",

                "Family member colonised", "", "", "","","","",

                "Family member occupational exposure", "Farming", "Hospital",

                "Human to nearby Environment", "","","","",

                "Livestock to drinking water", "Cattle", "Pig", "Poultry",

                "Mother to Child", "", "","",

                "Occupational Exposure", "Cattle", "Pig", "", "", "", "", "", #"",

                "Poultry", #"",

                "", "Veterinarian", "",

                "Pet to human", "", "", "",  "","horse", "Space sharing",

                "Travelling", "South Asia", "", "", "", "", "South East Asia", "", "Western Asia", "", "Central & East Asia", "Asia unspecified", "", "",

                "Latin America", "","", "America", "Africa", "", "", "Europe", "", "", "Animal mother to baby animal", "Showering daily", "Antibacterial soap",

                "Family member colonized T", "Family member colonized R", "Family member colonsied E coli risk")

Pathogen<-c(" ", "E.coli", "E.coli", "S. Aureus", "S. Aureus"," ", "S. pseudintermedius","S. areus", " ", "E. coli", "E.coli", "", "E. coli", "Enterobacteriaceae", "S. areus", "S. pneumoniae",

      "", "E.coli", "S. Aureus", "S. Aureus", "S. Areus","S. Aureus", "S. Aureus", "VRE", "A. Baumanni", "A. Baumanni","P. Aeruginosa","P. Aeruginosa", "S. epidermidis",

      "", "E. coli", "E. coli","E. coli", "E.coli", "", "E.Coli", "Enterobacteriaceae", "S. Aureus", "S. areus", "S. areus", "P.aeruginose", "", "S. Aureus", "S. Aureus",
```

```
        "","S. Aureus", "VRE", "A. baumanni","A. calcoaceticus", "", "E. coli","E. coli","E. coli", "", "S. Aureus", "S. Aureus","Group B Streptococci",
        "","S. Areus","Enterobacteriaceae","S.Aureus", "", "", "", "Staphylococci", #"",
        "E. coli", "S. Aureus", #"",
        "S. Areus", "",
        "","E. coli", "S.Aureus","S.Aureus","Staphylococci","S.Aureus", "Enterobacteria",
        "", "S. aureus", "E. coli", "E.coli", "Enterobacteriaceae","Enterobacteriaceae",
        "Enterobacteriaceae", "Enterobacteriaceae",
        "Enterobacteriaceae", "Enterobacteriaceae",
        "Enterobacteriaceae",
        "E. coli", "Enterobacteriaceae","Enterobacteriaceae",
        "E. coli", "Enterobacteriaceae", "Enterobacteriaceae",
        "Enterobacteriaceae",
        "E. coli", "Enterobacteriaceae","Enterobacteriaceae",
        "E. coli", "Enterobacteriaceae","Enterobacteriaceae",
        "Staphylococcus pseudintermedius",
        "S. aureus",
        "S. aureus",
        "S. aureus", "E. coli", "E. coli")

MethodofEstimation<- c("", "Risk", "Risk", "Risk", "Risk", " ", "Risk", "R0", " ", "Risk", "Risk", "", "OR", "OR", "Genes", "OR", "",
            "Risk", "Genes", "OR","R0", "Risk", "RR","Risk", "OR", "Risk", "Risk", "Genes", "Risk", "", "OR", "OR","OR","PR",
            "", "OR", "PR", "OR", "Risk", "Genes","Genes", "", "OR", "OR",
            "", "Risk", "Risk", "Risk", "Risk", "","OR", "OR","OR", "", "OR", "Risk","Risk",
            "", "OR", "Risk", "OR", "PR", "Risk", "Genes", "Risk", #"PR",
            "OR",#"OR",
            "PR", "OR","Risk",
            "", "OR", "Risk", "Genes", "OR","Genes","OR",
            "", "OR", "OR", "Risk", "OR", "Risk",
            "OR", "Risk",
```

```
            "OR", "Risk",
            "Risk",
            "Risk", "OR", "Risk",
            "Risk", "OR", "Risk",
            "OR",
            "Risk", "OR", "Risk",
            "Risk", "OR", "Risk",
            "Genes",
            "OR",
            "OR",
            "Genes", "Genes",
            "Risk")

Random_effects<-round((c(1/(1+exp(-model.AtoA_EcoliRisk_poultry@beta)), 1/(1+exp(-model.pig_to_air_Ecoli_Risk@beta)), 1/(1+exp(-model.pig_to_air_Saureus_Risk@beta)), 1/(1+exp(-model.cattle.to.air.risk@beta)))),3)

Random_effects<-c("", Random_effects,"", round((c(1/(1+exp(-model.pet_to_pet_speudo.risk@beta)), exp(r0.animalanimal$beta))),3))

Random_effects<-c(Random_effects,"",round((c(1/(1+exp(-model.poultry_to_env@beta)),1/(1+exp(-model.pig_to_env@beta)))),3), "",

        round((c(exp(or.breastf$beta), exp(or.breastf_entero$beta),1/(1+exp(-model.breast.areus@beta)),exp(or.breastf_spneumo$beta))),3), "",

        round((c(1/(1+exp(-model.cont.inf.ecoli_risk@beta)), #1/(1+exp(-model.cont.inf.entero@beta)),

            1/(1+exp(-model.cont.inf.saureus_genes@beta)), exp(or.cont.inf.saureus$beta),exp(r0.cont.inf.saureus$beta), 1/(1+exp(-model.cont.inf.saureus_Risk@beta)), exp(rr.contact.inf.saureus$beta),

            1/(1+exp(-model.cont.inf.vre_Risk@beta)), exp(or.cont.inf.abaum$beta), 1/(1+exp(-model.cont.inf.abau_Risk@beta)),

            1/(1+exp(-model.cont.inf.pau@beta)), 1/(1+exp(-model.cont.inf.pau.genes@beta)), 1/(1+exp(-model.cont.inf.epi.risk@beta)))),3), "",

        round(c(exp(or.white$beta),exp(or.red$beta),exp(or.general$beta), exp(pr.redmeat$beta)),3), "", round(c(exp(or.fam.mem.col.ecoli$beta), exp(pr.fam.mem.col.enter$beta), exp(or.fam.mem.col.saureus$beta),1/(1+exp(-model.fam_col_sau_risk@beta)), 1/(1+exp(-model.fam_col_sau_genes@beta)),1/(1+exp(-model.fam.pau.genes@beta))),3), "",
```

```
round(c(exp(or.fam.occu.farm$beta), exp(or.fam.occu.hosp$beta)),3),

"", round(c(1/(1+exp(-model.humantonearenv.saureus.risk@beta)),1/(1+exp(-model.humantonearenv.VRE.risk@beta)),1/(1+exp(-model.humantonearenv.abau.risk@beta)),1/(1+exp(-model.humantonearenv.acalco.risk@beta))),3), "",

round(c(exp(or.livedrink_cattle$beta), exp(or.livedrink_pig$beta), exp(or.livedrink_poultry$beta)),3), "",

round(c(exp(or.mother.to.child$beta), 1/(1+exp(-model.mother.to.child.risk@beta)),1/(1+exp(-model.mother.to.child.risk.GB@beta))),3), "",

round(c(exp(or.occu.exp.cattle.saureus$beta),  1/(1+exp(-model.occu.exp.pig.entero@beta)), exp(or.occu.exp.pig.saureus$beta), exp(pr.occu.exp.saureus.pig$beta),

 1/(1+exp(-model.occu.exp.pig.saureus@beta)),  1/(1+exp(-model.occu.exp.genes.saureus.pig@beta)),  1/(1+exp(-model.occu.exp.staph.risk.pig@beta)), #exp(pr.occu.exp.staph.pig$beta),

 exp(or.occu.exp.poultry$beta) ,#exp(or.occu.exp.poultry.saureus$beta),

 exp(pr.occu.exp.saureus.poultry$beta), exp(or.occu.exp.vets.sarues$beta), 1/(1+exp(-model.occu.exp.vet.risk@beta))),3), "",

round(c(exp(OR.pet.tp.human.ecoli$beta), 1/(1+exp(-model.risk.pethuman.saureus@beta)), 1/(1+exp(-modelGenes_Pet_to_human_saureus@beta)) ,exp(OR.pet.tp.human.staph$beta),1/(1+exp(-modelGenes_Pet_to_human_saureus_horse@beta)), exp(OR.sharingroom.or$beta)),3), "",

round(c(exp(OR.travel_SA_SAreus$beta), exp(OR.travel_SA_Ecoli$beta), 1/(1+exp(-model.risk.travel.SA.ecoli@beta)), exp(OR.travel_SA_Entero$beta), 1/(1+exp(-model.risk.travel.SA.entero@beta)),

exp(OR.travel_SEA_Entero$beta), 1/(1+exp(-model.risk.travel.SEA.entero@beta)),

exp(OR.travel_WA_entero$beta), 1/(1+exp(-model.risk.travel.wa.entero@beta)),

1/(1+exp(-model.risk.travel.centraleast.entero@beta)),

1/(1+exp(-model.risk.travel.asia.ecoli@beta)), exp(OR.travel_Asia_entero$beta),1/(1+exp(-model.risk.travel.asia.entero@beta)),

1/(1+exp(-model.risk.travel.ecoli.LA@beta)), exp(OR.travel_LA_entero$beta), 1/(1+exp(-model.risk.travel.LA_enter@beta)),

exp(OR.travel_Amerika_enter$beta),

1/(1+exp(-model.risk.travel.africa.ecoli@beta)), exp(OR.travel_Africa_enter$beta), 1/(1+exp(-model.risk.travel.africa.entero@beta)),

1/(1+exp(-model.risk.travel.eu.ecoli@beta)),exp(OR.travel_Europe_entero$beta), 1/(1+exp(-model.risk.travel.entero.eu@beta)),

1/(1+exp(-modelGenes_motheranimal_to_baby@beta)),
```

```
exp(OR.bathingshowering$beta), exp(OR.soap$beta),
1/(1+exp(-model.fam_col_sau_genes_T@beta)),
1/(1+exp(-model.Fam_col_ecoli_genes@beta)),
1/(1+exp(-model.Family_member_colonised_ecoli_risk@beta))),3))

LowerBR<-round(c(1/(1+exp(-(model.AtoA_EcoliRisk_poultry@beta-1.96*se.logit.AtoApoultry@x))), 1/(1+exp(-(model.pig_to_air_Ecoli_Risk@beta-1.96*se.logit.AtoApig@x))), 1/(1+exp(-(model.pig_to_air_Saureus_Risk@beta-1.96*se.logit.AtoApigAreus@x))),1/(1+exp(-(model.cattle.to.air.risk@beta-1.96*se.logit.AtoAcattle@x)))),3)

LowerBR<-c("", LowerBR," ", round(c(1/(1+exp(-(model.pet_to_pet_speudo.risk@beta-1.96*se.logit.pet.Spseudo@x))), exp(r0.animalanimal$ci.lb)),3))

LowerBR<-c(LowerBR, " ", round(c(1/(1+exp(-(model.poultry_to_env@beta-1.96*se.logit.poultry_env@x))), 1/(1+exp(-(model.pig_to_env@beta-1.96*se.logit.pig_env@x)))),3), "",

    round(c(exp(or.breastf$ci.lb), exp(or.breastf_entero$ci.lb),1/(1+exp(-(model.breast.areus@beta-1.96*se.logit.breast@x))) , exp(or.breastf_spneumo$ci.lb)),3), "",

    round((c(1/(1+exp(-(model.cont.inf.ecoli_risk@beta-1.96*se.logit.cont.inf.ecoli.risk@x))),

        #1/(1+exp(-(model.cont.inf.entero@beta-1.96*se.logit.cont.inf.entero@x))),

        1/(1+exp(-(model.cont.inf.saureus_genes@beta-1.96*se.logit.cont.inf.saureus.genes@x))),

        exp(or.cont.inf.saureus$ci.lb),exp(r0.cont.inf.saureus$ci.lb), 1/(1+exp(-(model.cont.inf.saureus_Risk@beta-1.96*se.logit.cont.inf.saureus.Risk@x))), exp(rr.contact.inf.saureus$ci.lb),1/(1+exp(-(model.cont.inf.vre_Risk@beta-1.96*se.logit.cont.inf.vre.Risk@x))),

        exp(or.cont.inf.abaum$ci.lb), 1/(1+exp(-(model.cont.inf.abau_Risk@beta-1.96*se.logit.cont.inf.abau@x))),1/(1+exp(-(model.cont.inf.pau@beta-1.96*se.logit.cont.inf.pau@x))),

        1/(1+exp(-(model.cont.inf.pau.genes@beta-1.96*se.logit.cont.inf.pau.genes@x))), 1/(1+exp(-(model.cont.inf.epi.risk@beta-1.96*se.logit.cont.inf.epi.risk@x))))),3), "",

    round(c(exp(or.white$ci.lb),exp(or.red$ci.lb),exp(or.general$ci.lb), exp(pr.meat_ecoli$ci.lb)),3), "", round(c(exp(or.fam.mem.col.ecoli$ci.lb), exp(pr.fam.mem.col.enter$ci.lb), exp(or.fam.mem.col.saureus$ci.lb),1/(1+exp(-(model.fam_col_sau_risk@beta-1.96*se.logit.fam_col_sau_risk@x))), 1/(1+exp(-(model.fam_col_sau_genes@beta-1.96*se.logit.fam_col_sau_genes@x))),1/(1+exp(-(model.fam.pau.genes@beta-1.96*se.logit.fam.pau.genes@x)))),3), "",

    round(c(exp(or.fam.occu.farm$ci.lb), exp(or.fam.occu.hosp$ci.lb)),3),"",

    round(c(1/(1+exp(-(model.humantonearenv.saureus.risk@beta-1.96*se.logithumantonearenv.saureus.risk@x))),1/(1+exp(-(model.humantonearenv.VRE.risk@beta-
```

```
1.96*se.logithumantonearenv.VRE.risk@x))),1/(1+exp(-(model.humantonearenv.abau.risk@beta-1.96*se.logithumantonearenv.abau.risk@x))),1/(1+exp(-(model.humantonearenv.acalco.risk@beta-1.96*se.logithumantonearenv.acalco.risk@x)))),3), "",

        round(c( exp(or.livedrink_cattle$ci.lb), exp(or.livedrink_pig$ci.lb), exp(or.livedrink_poultry$ci.lb)),3), "",

        round(c(exp(or.mother.to.child$ci.lb), 1/(1+exp(-(model.mother.to.child.risk@beta-1.96*se.logit.mother.to.child@x))), 1/(1+exp(-(model.mother.to.child.risk.GB@beta-1.96*se.logit.mother.to.child.GB@x)))),3),"",

        round(c(exp(or.occu.exp.cattle.saureus$ci.lb),  1/(1+exp(-(model.occu.exp.pig.entero@beta-1.96*se.logit.occu.exp.pig.entero@x))), exp(or.occu.exp.pig.saureus$ci.lb), exp(pr.occu.exp.saureus.pig$ci.lb),

                1/(1+exp(-(model.occu.exp.pig.saureus@beta-1.96*se.logit.occu.exp.pig.saureus@x))), 1/(1+exp(-(model.occu.exp.genes.saureus.pig@beta-1.96*se.logit.occu.exp.genes.saureus.pig@x))),1/(1+exp(-(model.occu.exp.staph.risk.pig@beta-1.96*se.logit.occu.exp.staph.risk.pig@x))), #exp(pr.occu.exp.staph.pig$ci.lb),

                exp(or.occu.exp.poultry$ci.lb) ,#exp(or.occu.exp.poultry.saureus$ci.lb),

                exp(pr.occu.exp.saureus.poultry$ci.lb), exp(or.occu.exp.vets.sarues$ci.lb), 1/(1+exp(-(model.occu.exp.vet.risk@beta-1.96*se.logit.occu.exp.vet.risk@x)))),3), "",

        round(c(exp(OR.pet.tp.human.ecoli$ci.lb),  1/(1+exp(-(model.risk.pethuman.saureus@beta-1.96*se.logit.risk.pethuman.saureus@x))), 1/(1+exp(-(modelGenes_Pet_to_human_saureus@beta-1.96*se.logitGenes_Pet_to_human_saureus@x))),exp(OR.pet.tp.human.staph$ci.lb), 1/(1+exp(-(modelGenes_Pet_to_human_saureus_horse@beta-1.96*se.logitGenes_Pet_to_human_saureus_horse@x))), exp(OR.sharingroom.or$ci.lb)),3), "",

        round(c(exp(OR.travel_SA_SAreus$ci.lb), exp(OR.travel_SA_Ecoli$ci.lb), 1/(1+exp(-(model.risk.travel.SA.ecoli@beta-1.96*se.logit.risk.travel.SA.ecoli@x))), exp(OR.travel_SA_Entero$ci.lb), 1/(1+exp(-(model.risk.travel.SA.entero@beta-1.96*se.logit.risk.travel.SA.entero@x))),

                exp(OR.travel_SEA_Entero$ci.lb), 1/(1+exp(-(model.risk.travel.SEA.entero@beta-1.96*se.logit.risk.travel.SEA.entero@x))),

                exp(OR.travel_WA_entero$ci.lb), 1/(1+exp(-(model.risk.travel.wa.entero@beta-1.96*se.logit.risk.travel.wa.entero@x))),

                1/(1+exp(-(model.risk.travel.centraleast.entero@beta-1.96*se.logit.risk.travel.centraleast.entero@x))),

                1/(1+exp(-(model.risk.travel.asia.ecoli@beta-1.96*se.logit.risk.travel.asia.ecoli@x))), exp(OR.travel_Asia_entero$ci.lb),1/(1+exp(-(model.risk.travel.asia.entero@beta-1.96*se.logit.risk.travel.asia.entero@x))),

                1/(1+exp(-(model.risk.travel.ecoli.LA@beta-1.96*se.logit.model.risk.travel.ecoli.LA@x))), exp(OR.travel_LA_entero$ci.lb), 1/(1+exp(-(model.risk.travel.LA_enter@beta-1.96*se.logit.risk.travel.LA_enter@x))),

                exp(OR.travel_Amerika_enter$ci.lb),
```

```
        1/(1+exp(-(model.risk.travel.africa.ecoli@beta-1.96*se.logit.risk.travel.africa.ecoli@x))),
exp(OR.travel_Africa_enter$ci.lb), 1/(1+exp(-(model.risk.travel.africa.entero@beta-
1.96*se.logit.risk.travel.africa.entero@x))),

        1/(1+exp(-(model.risk.travel.eu.ecoli@beta-
1.96*se.logit.risk.travel.eu.ecoli@x))),exp(OR.travel_Europe_entero$ci.lb), 1/(1+exp(-
(model.risk.travel.entero.eu@beta-1.96*se.logit.risk.travel.entero.eu@x))),

        1/(1+exp(-(modelGenes_motheranimal_to_baby@beta-
1.96*se.logitGenes_motheranimal_to_baby@x))),

        exp(OR.bathingshowering$ci.lb), exp(OR.soap$ci.lb),

        1/(1+exp(-(model.fam_col_sau_genes_T@beta-1.96*se.logit.fam_col_sau_genes_T@x))),

        1/(1+exp(-(model.Fam_col_ecoli_genes@beta-1.96*se.logit.Fam_col_ecoli_genes@x))),

        1/(1+exp(-(model.Family_member_colonised_ecoli_risk@beta-
1.96*se.logit.Family_member_colonised_ecoli_risk@x)))),3))


UpperBR<-round(c(1/(1+exp(-(model.AtoA_EcoliRisk_poultry@beta+1.96*se.logit.AtoApoultry@x))),
1/(1+exp(-(model.pig_to_air_Ecoli_Risk@beta+1.96*se.logit.AtoApig@x))), 1/(1+exp(-
(model.pig_to_air_Saureus_Risk@beta+1.96*se.logit.AtoApigAreus@x))),1/(1+exp(-
(model.cattle.to.air.risk@beta+1.96*se.logit.AtoAcattle@x)))),3)

UpperBR<-c(" ", UpperBR,"", round(c(1/(1+exp(-
(model.pet_to_pet_speudo.risk@beta+1.96*se.logit.pet.Spseudo@x))),
exp(r0.animalanimal$ci.ub)),3))

UpperBR<-c(UpperBR, " ", round(c(1/(1+exp(-
(model.poultry_to_env@beta+1.96*se.logit.poultry_env@x))), 1/(1+exp(-
(model.pig_to_env@beta+1.96*se.logit.pig_env@x)))),3), "",

    round(c(exp(or.breastf$ci.ub), exp(or.breastf_entero$ci.ub),1/(1+exp(-
(model.breast.areus@beta+1.96*se.logit.breast@x))) , exp(or.breastf_spneumo$ci.ub)),3),"",

    round((c(1/(1+exp(-(model.cont.inf.ecoli_risk@beta+1.96*se.logit.cont.inf.ecoli.risk@x))),

        #1/(1+exp(-(model.cont.inf.entero@beta+1.96*se.logit.cont.inf.entero@x))),

        1/(1+exp(-
(model.cont.inf.saureus_genes@beta+1.96*se.logit.cont.inf.saureus.genes@x))),

        exp(or.cont.inf.saureus$ci.ub), exp(r0.cont.inf.saureus$ci.ub),1/(1+exp(-
(model.cont.inf.saureus_Risk@beta+1.96*se.logit.cont.inf.saureus.Risk@x))),
exp(rr.contact.inf.saureus$ci.ub),1/(1+exp(-
(model.cont.inf.vre_Risk@beta+1.96*se.logit.cont.inf.vre.Risk@x))),

        exp(or.cont.inf.abaum$ci.ub), 1/(1+exp(-
(model.cont.inf.abau_Risk@beta+1.96*se.logit.cont.inf.abau@x))),1/(1+exp(-
(model.cont.inf.pau@beta+1.96*se.logit.cont.inf.pau@x))),
```

```
1/(1+exp(-(model.cont.inf.pau.genes@beta+1.96*se.logit.cont.inf.pau.genes@x))),
1/(1+exp(-(model.cont.inf.epi.risk@beta+1.96*se.logit.cont.inf.epi.risk@x))))),3), "",

round(c(exp(or.white$ci.ub),exp(or.red$ci.ub),exp(or.general$ci.ub),
exp(pr.meat_ecoli$ci.ub)),3), "", round(c(exp(or.fam.mem.col.ecoli$ci.ub),
exp(pr.fam.mem.col.enter$ci.ub), exp(or.fam.mem.col.saureus$ci.ub),1/(1+exp(-
(model.fam_col_sau_risk@beta+1.96*se.logit.fam_col_sau_risk@x))),1/(1+exp(-
(model.fam_col_sau_genes@beta+1.96*se.logit.fam_col_sau_genes@x))),1/(1+exp(-
(model.fam.pau.genes@beta+1.96*se.logit.fam.pau.genes@x)))),3), "",

round(c(exp(or.fam.occu.farm$ci.ub), exp(or.fam.occu.hosp$ci.ub)),3),"",

round(c(1/(1+exp(-
(model.humantonearenv.saureus.risk@beta+1.96*se.logithumantonearenv.saureus.risk@x))),1/(1+e
xp(-(model.humantonearenv.VRE.risk@beta+1.96*se.logithumantonearenv.VRE.risk@x))),1/(1+exp(-
(model.humantonearenv.abau.risk@beta+1.96*se.logithumantonearenv.abau.risk@x))),1/(1+exp(-
(model.humantonearenv.acalco.risk@beta+1.96*se.logithumantonearenv.acalco.risk@x)))),3), "",

round(c( exp(or.livedrink_cattle$ci.ub), exp(or.livedrink_pig$ci.ub),
exp(or.livedrink_poultry$ci.ub)),3),"",

round(c(exp(or.mother.to.child$ci.ub), 1/(1+exp(-
(model.mother.to.child.risk@beta+1.96*se.logit.mother.to.child@x))), 1/(1+exp(-
(model.mother.to.child.risk.GB@beta+1.96*se.logit.mother.to.child.GB@x)))),3),"",

round(c(exp(or.occu.exp.cattle.saureus$ci.ub),  1/(1+exp(-
(model.occu.exp.pig.entero@beta+1.96*se.logit.occu.exp.pig.entero@x))),
exp(or.occu.exp.pig.saureus$ci.ub), exp(pr.occu.exp.saureus.pig$ci.ub),

1/(1+exp(-(model.occu.exp.pig.saureus@beta+1.96*se.logit.occu.exp.pig.saureus@x))),
1/(1+exp(-
(model.occu.exp.genes.saureus.pig@beta+1.96*se.logit.occu.exp.genes.saureus.pig@x))),1/(1+exp(-
(model.occu.exp.staph.risk.pig@beta+1.96*se.logit.occu.exp.staph.risk.pig@x))),
#exp(pr.occu.exp.staph.pig$ci.ub),

exp(or.occu.exp.poultry$ci.ub) ,#exp(or.occu.exp.poultry.saureus$ci.ub),

exp(pr.occu.exp.saureus.poultry$ci.ub), exp(or.occu.exp.vets.sarues$ci.ub), 1/(1+exp(-
(model.occu.exp.vet.risk@beta+1.96*se.logit.occu.exp.vet.risk@x)))),3), "",

round(c(exp(OR.pet.tp.human.ecoli$ci.ub),1/(1+exp(-
(model.risk.pethuman.saureus@beta+1.96*se.logit.risk.pethuman.saureus@x))), 1/(1+exp(-
(modelGenes_Pet_to_human_saureus@beta+1.96*se.logitGenes_Pet_to_human_saureus@x))),exp
(OR.pet.tp.human.staph$ci.ub),1/(1+exp(-
(modelGenes_Pet_to_human_saureus_horse@beta+1.96*se.logitGenes_Pet_to_human_saureus_h
orse@x))), exp(OR.sharingroom.or$ci.ub)),3),"",

round(c(exp(OR.travel_SA_SAreus$ci.ub), exp(OR.travel_SA_Ecoli$ci.ub), 1/(1+exp(-
(model.risk.travel.SA.ecoli@beta+1.96*se.logit.risk.travel.SA.ecoli@x))),
exp(OR.travel_SA_Entero$ci.ub), 1/(1+exp(-
(model.risk.travel.SA.entero@beta+1.96*se.logit.risk.travel.SA.entero@x))),
```

```
                exp(OR.travel_SEA_Entero$ci.ub), 1/(1+exp(-
(model.risk.travel.SEA.entero@beta+1.96*se.logit.risk.travel.SEA.entero@x))),

                exp(OR.travel_WA_entero$ci.ub), 1/(1+exp(-
(model.risk.travel.wa.entero@beta+1.96*se.logit.risk.travel.wa.entero@x))),

                1/(1+exp(-
(model.risk.travel.centraleast.entero@beta+1.96*se.logit.risk.travel.centraleast.entero@x))),

                1/(1+exp(-(model.risk.travel.asia.ecoli@beta+1.96*se.logit.risk.travel.asia.ecoli@x))),
exp(OR.travel_Asia_entero$ci.ub),1/(1+exp(-
(model.risk.travel.asia.entero@beta+1.96*se.logit.risk.travel.asia.entero@x))),

                1/(1+exp(-(model.risk.travel.ecoli.LA@beta+1.96*se.logit.model.risk.travel.ecoli.LA@x))),
exp(OR.travel_LA_entero$ci.ub), 1/(1+exp(-
(model.risk.travel.LA_enter@beta+1.96*se.logit.risk.travel.LA_enter@x))),

                exp(OR.travel_Amerika_enter$ci.ub),

                1/(1+exp(-(model.risk.travel.africa.ecoli@beta+1.96*se.logit.risk.travel.africa.ecoli@x))),
exp(OR.travel_Africa_enter$ci.ub), 1/(1+exp(-
(model.risk.travel.africa.entero@beta+1.96*se.logit.risk.travel.africa.entero@x))),

                1/(1+exp(-(model.risk.travel.eu.ecoli@beta+1.96*se.logit.risk.travel.eu.ecoli@x))),
exp(OR.travel_Europe_entero$ci.ub), 1/(1+exp(-
(model.risk.travel.entero.eu@beta+1.96*se.logit.risk.travel.entero.eu@x))),

                1/(1+exp(-
(modelGenes_motheranimal_to_baby@beta+1.96*se.logitGenes_motheranimal_to_baby@x))),

                exp(OR.bathingshowering$ci.ub), exp(OR.soap$ci.ub),

                1/(1+exp(-(model.fam_col_sau_genes_T@beta+1.96*se.logit.fam_col_sau_genes_T@x))),

                1/(1+exp(-(model.Fam_col_ecoli_genes@beta+1.96*se.logit.Fam_col_ecoli_genes@x))),

                1/(1+exp(-
(model.Family_member_colonised_ecoli_risk@beta+1.96*se.logit.Family_member_colonised_ecoli_
risk@x)))),3))


tabel_meta<-data.frame("Transmission Route"=TransmissionRoute, "Pathogen"=Pathogen, "Method
of Estimation"= MethodofEstimation, "Random Effect"=Random_effects, "95CI Lower
Bound"=LowerBR, "95CI Upper bound"=UpperBR)

tabel_meta


#tabel_meta$Random.Effect<-as.numeric(tabel_meta$Random.Effect)

#tabel_meta$Random.Effect<-round(tabel_meta$Random.Effect, digits=2)

#tabel_meta$X95CI.Lower.Bound<-as.numeric(tabel_meta$X95CI.Lower.Bound)

#tabel_meta$X95CI.Lower.Bound<-round(tabel_meta$X95CI.Lower.Bound, digits=2)
```

```
#tabel_meta$X95CI.Upper.bound<-as.numeric(tabel_meta$X95CI.Upper.bound)

#tabel_meta$X95CI.Upper.bound<-round(tabel_meta$X95CI.Upper.bound, digits=2)

# Rounding for in paper


write.xlsx(tabel_meta, "PATH TO YOUR PC/Data meta03022021.xlsx")

# Replace "PATH TO YOUR PC" with the location you want to save the data in.

#WATCH OUT! If you want to run the forest plot script, do not run the underneath "rm" part




# Clean up environment leaving only the dataset

rm(Random_effects, LowerBR, UpperBR)

rm(model.AtoA_EcoliRisk_poultry, model.pig_to_air_Ecoli_Risk, model.poultry_to_env,
model.pig_to_air_Saureus_Risk, model.cattle.to.air.risk,

   model.pet_to_pet_speudo.risk, model.pig_to_env, or.breastf, or.breastf_entero,
model.breast.areus, or.breastf_spneumo,

   model.cont.inf.ecoli_risk,
model.cont.inf.saureus_genes,model.cont.inf.saureus_Risk,model.cont.inf.abau_Risk,
model.cont.inf.vre_Risk,

   model.cont.inf.epi.risk, model.cont.inf.pau, model.cont.inf.pau.genes, Travelling,
Travelling_OR_Ecoli, Travelling_OR_Ecoli_SA, Travelling_OR_Saereus,

   Travelling_OR_Entero, Travelling_OR_Entero_Africa, Travelling_OR_Entero_America,
Travelling_OR_Entero_Asia_unsp,

   Travelling_OR_Entero_Europe, Travelling_OR_Entero_LatinAmerica, Travelling_OR_Entero_SA,
Travelling_OR_Entero_SEA, Travelling_OR_Entero_WA, Travelling_Risk_entero_WA,
Travelling_Risk_entero_SEA,

   Travelling_Risk_entero_SA, Travelling_Risk_entero_LA, Travelling_Risk_entero_EU,
Travelling_Risk_entero_CentralEastA, Travelling_Risk_entero_Asia_unspec,
Travelling_Risk_entero_africa,

   Travelling_Risk_entero, Travelling_Risk_ecoli_LA, Travelling_Risk_ecoli_eu,
Travelling_Risk_ecoli_Asia_un, Travelling_Risk_ecoli_Africa, Travelling_Risk_ecoli,
Travelling_OR_saures_South_asia,

   Travelling_PR, Travelling_Risk, Acquisition, Animal_contact, Animal_to_air,
Animal_to_air_Ecoli_Risk, Animal_to_air_Sareus_Risk, Animal_to_animal, Animal_to_environment,

   Animal_to_environment_Ecoli, aureus, BacterialIntake, breast_areus, Breast_feeding,
BreastF_ecoli, BreastF_ecoli_OR, animal_to_animal_Saurues, animal_to_animal_Saurues_r0,
```

BreastF_spneumo_OR, BreastF_spneumo, BreastF_entero_OR, BreastF_entero, Casesperday, chicken_to_chicken_EColiTR, TRratio, whitemeat_or, Travelling_Risk_ecoli_SA, Table_Modelling_methods, Table_Statistical_methods,

air_to_animal, Animal_animal_risk_other, Cattle_to_air_Sareus_Risk, cattle_to_cattle_Saureus, Contact_with_infected, Contact_with_infected_Abau_OR, Contact_with_infected_Abau_Risk,

Cont_Infeced_ecoli_Risk, Contact_with_infected_VRE_Risk, Contact_with_infected_VRE_R0, Contact_with_infected_VRE, Contact_with_infected_Saurues_TR, Contact_with_infected_Saurues_RR,

Contact_with_infected_Saurues_Risk, Contact_with_infected_Saurues_OR, Contact_with_infected_Saurues_IOR, Contact_with_infected_Saurues_Genes, Contact_with_infected_Saurues_Cases,

Contact_with_infected_paerug_risk, Contact_with_infected_paerug_IOR, Contact_with_infected_paerug_genes, Contact_with_infected_epi_risk, Contact_with_infected_Abau_R0, Contact_with_infected_Saurues_r0,

Contaminated_room, Contaminated_room_VRE, Eating_meat, Eating_meat_E.coli_OR, Eating_meat_E.coli_PR, Fam_col_ecoli_genes, Fam_with_infected_paerug_genes,Family_member_colonised,

Family_member_colonised_ecoli_OR, fam_col_sau_genes,fam_col_sau_genes_T,fam_col_sau_risk, environement_to_human, Family_member_colonised_ecoli_risk, Family_member_colonised_entero_PR,

Family_member_colonised_saureus_OR, Family_member_occupational_exposure_VRE, Family_member_occupational_exposure_saureus_hospital, Family_member_occupational_exposure_saureus_farming, Family_member_occupational_exposure_saureus,

Family_member_occupational_exposure, Genes, Genes_motheranimal_to_baby, Genes_Pet_to_human_saureus, Genes_Pet_to_human_saureus_horse, generalmeat_or, human_to_air,

Human_to_nearbyenv, Human_to_nearbyenv_abau_Risk, Human_to_nearbyenv_acalco_Risk, Human_to_nearbyenv_Saureus_Risk, Human_to_nearbyenv_VRE_Risk,

food_to_animal, Incidence, Interventions, Incidence_rate_ratio, kiekje, Livestock_to_drinking_water, Livestock_to_drinking_water_cattle, Livestock_to_drinking_water_pig,

Livestock_to_drinking_water_poultry, Modelling, modelGenes_Pet_to_human_saureus_horse, modelGenes_Pet_to_human_saureus, modelGenes_motheranimal_to_baby, model.risk.travel.wa.entero,

model.risk.travel.SEA.entero, model.risk.travel.SA.entero, model.risk.travel.SA.ecoli, model.risk.travel.LA_enter, model.risk.travel.eu.ecoli, model.risk.travel.entero.eu, model.risk.travel.ecoli.LA,

model.risk.travel.centraleast.entero, model.risk.travel.asia.entero, model.risk.travel.asia.ecoli, model.risk.travel.africa.entero, model.risk.travel.africa.ecoli, model.risk.pethuman.saureus,

model.occu.exp.vet.risk, model.occu.exp.staph.risk.pig, model.occu.exp.pig.saureus, model.occu.exp.pig.entero, model.occu.exp.genes.saureus.pig, model.fam.pau.genes,

model.Fam_col_ecoli_genes, model.mother.to.child.risk, model.fam_col_sau_genes, model.fam_col_sau_genes_T, model.humantonearenv.VRE.risk, model.fam_col_sau_risk, model.Family_member_colonised_ecoli_risk,

model.humantonearenv.abau.risk, model.humantonearenv.acalco.risk, model.humantonearenv.saureus.risk, model.mother.to.child.risk.GB,motheranimal_to_baby, Mother_to_child,

Mother_to_child_Aaureus_OR, Mother_to_child_entero_Risk, Mother_to_child_groupB_Risk, Mother_to_child_Saureus_Risk, Nearby_env_tohuman, Non_commercial_animal_keeping,

Occupational_exp_genes, Occupational_exp_or, Occupational_exp_PR, Occupational_exp_risk, Occupational_Exposure, Occupational_Exposure_E.coli_Genes, Occupational_Exposure_E.coli_OR_poultry,

Occupational_Exposure_E.coli_PR, Occupational_Exposure_E.coli_Risk, Occupational_Exposure_Entero_genes, Occupational_Exposure_Entero_OR, Occupational_Exposure_Entero_Risk,

Occupational_Exposure_VRE, Occupational_Exposure_Staph_pig_risk, Occupational_Exposure_Staph_pig_PR, Occupational_Exposure_Staph, Occupational_Exposure_SAureus_Risk_vet,

Occupational_Exposure_SAureus_Risk_pig, Occupational_Exposure_SAureus_Risk, Occupational_Exposure_SAureus_PR_poultry, Occupational_Exposure_SAureus_PR_Pig,

Occupational_Exposure_SAureus_PR, Occupational_Exposure_SAureus_OR_vets, Occupational_Exposure_SAureus_OR_pig, Occupational_Exposure_SAureus_OR_cattle, Occupational_Exposure_SAureus_OR,

Occupational_Exposure_SAureus_Genes, Occupational_Exposure_Entero_Risk_pig, Occupational_Exposure_SAureus_Genes_pig, Occupational_Exposure_haemo_PR_pig, Occupational_Exposure_epi,

OR, OR_Animal_contact, OR_Contact_inf_person, OR_Contaminated_room, OR_Eating_Meat, OR_Family_mem_colonised, OR_Family_member_occu_exp, OR_Fomites, OR_Livestock_to_drink_water,OR_Mother_to_Child,

OR_Nearbyfarm_to_human, OR_Occupational_Exp, OR_Pet_to_human, OR_Prior_col_roomoccu, OR_Space_sharing, OR_Travelling, OR_Waterdrink_to_human,

OR.bathingshowering, or.cont.inf.abaum, or.cont.inf.saureus, or.fam.mem.col.ecoli, or.fam.mem.col.saureus, or.fam.occu.farm,or.fam.occu.hosp, or.general, or.livedrink_cattle, or.livedrink_pig, or.livedrink_poultry,

or.meat_ecoli, or.mother.to.child, or.occu.exp.cattle.saureus, or.occu.exp.pig.saureus, or.occu.exp.poultry, or.occu.exp.vets.sarues, OR.pet.tp.human.staph, OR.pet.tp.human.ecoli,

or.red, OR.sharingroom.or, OR.soap, OR.travel_Africa_enter, OR.travel_Amerika_enter, OR.travel_Asia_entero, OR.travel_Europe_entero, OR.travel_LA_entero,

OR.travel_SA_Ecoli, OR.travel_SA_SAreus, OR.travel_SEA_Entero, OR.travel_WA_entero, OR.travel_SA_Entero, or.white,

organtransfer_risk, Other_Bathing_Showering, Other_soap, Pet_to_human, Pet_to_human_ecoli_OR, Pet_to_human_entero_OR, Pet_to_human_staph_OR, pet_to_pet, pet_to_pet_risk_speudi,

pig_to_air_Ecoli_Risk, pig_to_env_ecoli, pig_to_pig_Saureus, Pig_to_air_Sareus_Risk,

poultry_to_air_Ecoli_Risk, poultry_to_env, PR, PR_alles, PR_Fam_Col, PR_Fam_Occu_Exp, PR_Occupation_Exp, PR_Pet_Human, PR_SpaceShare, PR_Travel,pr.fam.mem.col.enter,

pr.meat_ecoli, pr.occu.exp.saureus.pig, pr.occu.exp.saureus.poultry, pr.redmeat,

Prior_col_patient, Prior_col_patient_abau, Prior_col_patient_pseu, r0.animalanimal, r0.cont.inf.saureus, red_pr, Risk,

redmeat_or, Risk_Animal_to_water, Risk_Animal_to_Air, Risk_Animal_to_Animal, Risk_Animal_to_environment, Risk_Cont_Inf_Person, Risk_Fam_mem_occu_exposure, Risk_Fomites,

Risk_Food_to_animal, Risk_Household_Col, Risk_hum_to_nearenvironment, Risk_Human_to_Air, Risk_Intervention, Risk_Water_drink_Human, Risk_Water_exposure_to_human,

Risk_Travelling, Risk_Pet_to_human_saureus, Risk_Pet_to_human, Risk_Organ, Risk_Occupational_Exposure, Risk_Motheranimal_to_animalchild, Risk_Mother_to_child,

R0_VRE_humantonrear, RR, rr.contact.inf.saureus, se.logit.AtoAcattle, se.logit.AtoApig, se.logit.AtoApigAreus, se.logit.AtoApoultry, se.logit.breast, se.logit.cont.inf.abau,

se.logit.cont.inf.ecoli.risk, se.logit.cont.inf.epi.risk, se.logit.cont.inf.pau, se.logit.cont.inf.pau.genes, se.logit.cont.inf.saureus.Risk, se.logithumantonearenv.VRE.risk, se.logithumantonearenv.acalco.risk, se.logitGenes_Pet_to_human_saureus_horse,

se.logitGenes_Pet_to_human_saureus, se.logit.risk.travel.wa.entero, se.logit.risk.travel.SA.entero, se.logit.risk.travel.SEA.entero, se.logit.risk.travel.LA_enter, se.logit.risk.travel.eu.ecoli,

se.logit.risk.travel.entero.eu, se.logit.risk.travel.centraleast.entero,

se.logit.cont.inf.saureus.genes, se.logit.cont.inf.vre.Risk, se.logit.fam.pau.genes, se.logit.Fam_col_ecoli_genes, se.logit.fam_col_sau_genes_T, se.logit.fam_col_sau_risk,

se.logit.Family_member_colonised_ecoli_risk, se.logit.mother.to.child,se.logit.model.risk.travel.ecoli.LA,se.logit.mother.to.child.GB,

se.logit.fam_col_sau_genes, se.logit.occu.exp.genes.saureus.pig, se.logit.occu.exp.pig.entero, se.logit.occu.exp.pig.saureus,

se.logithumantonearenv.saureus.risk, se.logitGenes_motheranimal_to_baby, se.logit.occu.exp.staph.risk.pig, se.logit.occu.exp.vet.risk, se.logit.pet.Spseudo,

se.logit.pig_env, se.logit.poultry_env, se.logit.risk.pethuman.saureus,

se.logit.risk.travel.africa.ecoli, se.logithumantonearenv.abau.risk, se.logit.risk.travel.SA.ecoli, se.logit.risk.travel.africa.entero, se.logit.risk.travel.asia.ecoli, se.logit.risk.travel.asia.entero,

```
   Sharing_room, Sharing_room_entero_OR, Sharing_water_source_with_animals, Space_sharing,
Stats, veggies, water_to_plant,

   Bacteria_Animal_to_Air, Bacteria_Animal_to_environment, Bacteria_Breast_feeding,
Bacteria_Contaminated_room, Bacteria_Contact_with_infected, Bacteria_Eating_meat,

   Bacteria_Fam_Occu_Exp, Bacteria_Family_mem_colonised, Bacteria_human_to_nearbyenv,
Bacteria_livestock_to_drinkw, Bacteria_Non_commercial_animal_keeping, Bacteria_Occupational,

   Bacteria_Mother_to_child, Bacteria_Pet_to_human, Bacteria_Travelling,
Bacteria_prior_col_patient, Bacteria_Sharing_water_source_with_animals, Bacteria_space_sharing,

   Countries, Methods, MethodofEstimation, Pathogen, Table_Country, test, weights,
Table_TransmissionRouteGroup, TransmissionRoute)



############################################################## Extra printing we did

Author_title_transg_bact<-Transmission[,c(21,22,2,35,6,34,18,17)]

write.xlsx(Author_title_transg_bact, "PATH TO YOUR PC/file.xlsx")

#REPLACE PATH TO YOUR PC with the location on you pc where you want to save the data

#view(Transmission)


#### Inspect pooled estimates

Pooled_AMS_AMR<-subset(Transmission, Transmission$Defined.as.resistant=="both")

rm(Pooled_AMS_AMR)
```
