# Supplementary material for: Transmission routes of antibiotic resistant bacteria: a systematic review
Source: BMC Infect Dis. 2022 May 20;22:482. doi: 10.1186/s12879-022-07360-z (PMC9123679; doi:10.1186/s12879-022-07360-z)
Supplement: Supplementary file 4 — Additional file 4. R script of the forest plots for the systematic review of Godijk et al. [file 12879_2022_7360_MOESM4_ESM.pdf]

Additional file 4. R script of the forest plots for the systematic review of Godijk et al.

```
#Be sure you first run the file "Script Systematic Review Godijk et al." until line 1924

#rm(list=ls())


library(readxl)

library(metafor)


Forest<- Eating_meat_E.coli_OR [,c(2,21,23, 34, 31, 32)]



par(xpd=FALSE)




# ODDS RATIO's


# Eating meat

forest(Eating_meat_E.coli_OR$Num_Estimate, ci.lb=Eating_meat_E.coli_OR$Num_lb, ci.ub=Eating_meat_E.coli_OR$Num_ub, psize=1, xlab="",

      xlim=c(-10,50), slab=Eating_meat_E.coli_OR$Author, cex=0.95, ylim=c(0,20), rows=c(1:11), refline = 1, alim=c(0, 20))


# Sharing water

forest(Sharing_water_source_with_animals$Num_Estimate, ci.lb=Sharing_water_source_with_animals$Num_lb, ci.ub=Sharing_water_source_with_animals$Num_ub, psize=1, xlab="Odds ratio",

      xlim=c(-5,26),slab=Sharing_water_source_with_animals$Author, cex=0.95, ylim=c(0,8), rows=c(1:5), refline = 1, alim=c(0, 20))

text(18,5.5, "Sharing water source with animals E. coli OR", pos=2,cex=1,font=2)


# Breast feeding

  # E. coli

forest(BreastF_ecoli_OR$Num_Estimate, ci.lb=BreastF_ecoli_OR$Num_lb, ci.ub=BreastF_ecoli_OR$Num_ub, psize=1, xlab="",
```

```
    xlim=c(-5,5),slab=BreastF_ecoli_OR$Author, cex=0.95, ylim=c(0,6), rows=c(1:3), refline = 1,
alim=c(0, 20))

text(18,3.5, "Breast feeding E. Coli OR", pos=2,cex=1,font=2)


  # Enterobacteriae

forest(BreastF_entero_OR$Num_Estimate, ci.lb=BreastF_entero_OR$Num_lb,
ci.ub=BreastF_entero_OR$Num_ub, psize=1, xlab="",

    xlim=c(-5,26),slab=BreastF_entero_OR$Author, cex=0.95, ylim=c(0,5), rows=c(1:2), refline = 1,
alim=c(0, 20))

text(17,2.5, "Breast feeding E. Coli Enterobacteriaceae", pos=2,cex=1,font=2)


  # S pneumonia

forest(BreastF_spneumo_OR$Num_Estimate, ci.lb=BreastF_spneumo_OR$Num_lb,
ci.ub=BreastF_spneumo_OR$Num_ub, psize=1, xlab="",

    xlim=c(-5,26),slab=BreastF_spneumo_OR$Author, cex=0.95, ylim=c(0,7), rows=c(1:3), refline = 1,
alim=c(0, 20))

text(17,4.5, "Breast feeding S. pneumoniae", pos=2,cex=1,font=2)


# Contact with infected person

  # S Aureus

forest(Contact_with_infected_Saurues_OR$Num_Estimate,
ci.lb=Contact_with_infected_Saurues_OR$Num_lb,
ci.ub=Contact_with_infected_Saurues_OR$Num_ub, psize=1, xlab="",

    xlim=c(-5,26),slab=Contact_with_infected_Saurues_OR$Author, cex=0.95, ylim=c(0,5),
rows=c(1:2), refline = 1, alim=c(0, 20))

text(17,2.5, "Contact with infected person S. Aureus OR", pos=2,cex=1,font=2)

  # A baumannii

forest(Contact_with_infected_Abau_OR$Num_Estimate,
ci.lb=Contact_with_infected_Abau_OR$Num_lb, ci.ub=Contact_with_infected_Abau_OR$Num_ub,
psize=1, xlab="",

    xlim=c(-5,26),slab=Contact_with_infected_Abau_OR$Author, cex=0.95, ylim=c(0,5), rows=c(1:2),
refline = 1, alim=c(0, 20))

text(17,2.5, "Contact with infected person A. baumanni OR", pos=2,cex=1,font=2)


# Eating meat
```

```
#Red

redmeat_or$Author<-as.character(redmeat_or$Author)

redmeat_or$Author[1:3]<-"Leistner"

forest(redmeat_or$Num_Estimate, ci.lb=redmeat_or$Num_lb, ci.ub=redmeat_or$Num_ub, psize=1, xlab="",
       xlim=c(-5,26), slab=redmeat_or$Author, cex=0.95, ylim=c(1,10), rows=c(1:6), refline = 1, alim=c(0, 20))

text(17,7, "Eating Red meat E. coli OR", pos=2,cex=1,font=2)

# White

whitemeat_or$Author<-as.character(whitemeat_or$Author)

whitemeat_or$Author[1]<-"Leistner"

forest(whitemeat_or$Num_Estimate, ci.lb=whitemeat_or$Num_lb, ci.ub=whitemeat_or$Num_ub, psize=1, xlab="",
       xlim=c(-5,26), slab=whitemeat_or$Author, cex=0.95, ylim=c(1,5), rows=c(1:2), refline = 1, alim=c(0, 20))

text(17,2.5, "Eating white meat E. coli OR", pos=2,cex=1,font=2)

# general

forest(generalmeat_or$Num_Estimate, ci.lb=generalmeat_or$Num_lb, ci.ub=generalmeat_or$Num_ub, psize=1, xlab="",
       xlim=c(-5,26), slab=generalmeat_or$Author, cex=0.95, ylim=c(0,7), rows=c(1:3), refline = 1, alim=c(0, 20))

text(17,4, "Eating meat in general E. coli OR", pos=2,cex=1,font=2)

# Family member colonised

# E.coli

forest(Family_member_colonised_ecoli_OR$Num_Estimate, ci.lb=Family_member_colonised_ecoli_OR$Num_lb, ci.ub=Family_member_colonised_ecoli_OR$Num_ub, psize=1, xlab="",
       xlim=c(-5,26), slab=Family_member_colonised_ecoli_OR$Author, cex=0.95, ylim=c(0,7), rows=c(1:4), refline = 1, alim=c(0, 20))

text(17,4.5, "Family member colonised E. coli OR", pos=2,cex=1,font=2)
```

```
# S aureus

Family_member_colonised_saureus_OR$Author<-
as.character(Family_member_colonised_saureus_OR$Author)

Family_member_colonised_saureus_OR$Author[1]<-"Ferreira"

forest(Family_member_colonised_saureus_OR$Num_Estimate,
ci.lb=Family_member_colonised_saureus_OR$Num_lb,
ci.ub=Family_member_colonised_saureus_OR$Num_ub, psize=1, xlab="",

      xlim=c(-7,26), slab=Family_member_colonised_saureus_OR$Author, cex=0.95, ylim=c(0.8,10),
rows=c(1:6), refline = 1, alim=c(0, 20))

text(17,7, "Family member colonised S. aureus OR", pos=2,cex=1,font=2)



# Family member occupational exposure

Family_member_occupational_exposure_saureus_farming$Author<-
as.character(Family_member_occupational_exposure_saureus_farming$Author)

Family_member_occupational_exposure_saureus_farming$Author[1:3]<-"Wardyn"

forest(Family_member_occupational_exposure_saureus_farming$Num_Estimate,
ci.lb=Family_member_occupational_exposure_saureus_farming$Num_lb,
ci.ub=Family_member_occupational_exposure_saureus_farming$Num_ub, psize=1, xlab="",

      xlim=c(-5,26), slab=Family_member_occupational_exposure_saureus_farming$Author,
cex=0.95, ylim=c(0.8,7), rows=c(1:4), refline = 1, alim=c(0, 20))

text(23,4.7, "Family member occupational exposure farming S. aureus OR", pos=2,cex=1,font=2)


#hospital

forest(Family_member_occupational_exposure_saureus_hospital$Num_Estimate,
ci.lb=Family_member_occupational_exposure_saureus_hospital$Num_lb,
ci.ub=Family_member_occupational_exposure_saureus_hospital$Num_ub, psize=1, xlab="",

      xlim=c(-5,26), slab=Family_member_occupational_exposure_saureus_hospital$Author,
cex=0.95, ylim=c(0,5), rows=c(1:2), refline = 1, alim=c(0, 20))

text(21,2.7, "Family member occupational exposure hospital S. aureus OR", pos=2,cex=1,font=2)


#Livestock to drinking water

#Cattle
```

forest(Livestock_to_drinking_water_cattle$Num_Estimate, ci.lb=Livestock_to_drinking_water_cattle$Num_lb, ci.ub=Livestock_to_drinking_water_cattle$Num_ub, psize=1, xlab="",

xlim=c(-5,26), slab=Livestock_to_drinking_water_cattle$Author, cex=0.95, ylim=c(0,7), rows=c(1:4), refline = 1, alim=c(0, 20))

text(17,4.7, "Livestock (cattle) to drinking water E. coli OR", pos=2,cex=1,font=2)

#Poultry

forest(Livestock_to_drinking_water_poultry$Num_Estimate, ci.lb=Livestock_to_drinking_water_poultry$Num_lb, ci.ub=Livestock_to_drinking_water_poultry$Num_ub, psize=1, xlab="",

xlim=c(-5,26), slab=Livestock_to_drinking_water_poultry$Author, cex=0.95, ylim=c(0,7), rows=c(1:4), refline = 1, alim=c(0, 20))

text(17,4.7, "Livestock (poultry) to drinking water E. coli OR", pos=2,cex=1,font=2)

#Pig

forest(Livestock_to_drinking_water_pig$Num_Estimate, ci.lb=Livestock_to_drinking_water_pig$Num_lb, ci.ub=Livestock_to_drinking_water_pig$Num_ub, psize=1, xlab="",

xlim=c(-5,26), slab=Livestock_to_drinking_water_pig$Author, cex=0.95, ylim=c(0,7), rows=c(1:4), refline = 1, alim=c(0, 20))

text(17,4.7, "Livestock (pig) to drinking water E. coli OR", pos=2,cex=1,font=2)

Mother to child

forest(Mother_to_child_Aaureus_OR$Num_Estimate, ci.lb=Mother_to_child_Aaureus_OR$Num_lb, ci.ub=Mother_to_child_Aaureus_OR$Num_ub, psize=1, xlab="",

xlim=c(-5,26), slab=Mother_to_child_Aaureus_OR$Author, cex=0.95, ylim=c(0,5), rows=c(1:2), refline = 1, alim=c(0, 20))

text(12,2.7, "Mother to child S. aureus OR", pos=2,cex=1,font=2)

Occupational exposure

#Cattle

forest(Occupational_Exposure_SAureus_OR_cattle$Num_Estimate, ci.lb=Occupational_Exposure_SAureus_OR_cattle$Num_lb, ci.ub=Occupational_Exposure_SAureus_OR_cattle$Num_ub, psize=1, xlab="",

```
    xlim=c(-5,26), slab=Occupational_Exposure_SAureus_OR_cattle$Author, cex=0.95, ylim=c(0,7),
rows=c(1:4), refline = 1, alim=c(0, 20))

text(17,4.7, "Occupational exposure to cattle S. aureus OR", pos=2,cex=1,font=2)


#Pig

forest(Occupational_Exposure_SAureus_OR_pig$Num_Estimate,
ci.lb=Occupational_Exposure_SAureus_OR_pig$Num_lb,
ci.ub=Occupational_Exposure_SAureus_OR_pig$Num_ub, psize=1, xlab="",

    xlim=c(-45,40), slab=Occupational_Exposure_SAureus_OR_pig$Author, cex=0.95, ylim=c(1,20),
rows=c(1:14), refline = 1, alim=c(0, 20))

text(90,16.7, "Occupational exposure to pig S. aureus OR", pos=2,cex=1,font=2)


forest(Occupational_Exposure_Entero_OR_pig$Num_Estimate,
ci.lb=Occupational_Exposure_Entero_OR_pig$Num_lb,
ci.ub=Occupational_Exposure_Entero_OR_pig$Num_ub, psize=1, xlab="",

    xlim=c(-5,26), slab=Occupational_Exposure_Entero_OR_pig$Author, cex=0.95, ylim=c(1,5),
rows=c(1:2), refline = 1, alim=c(0, 20))

text(23,2.7, "Occupational exposure to pig Enterobacteriaceae OR", pos=2,cex=1,font=2)


#Poultry

# E. coli

forest(Occupational_Exposure_E.coli_OR_poultry$Num_Estimate,
ci.lb=Occupational_Exposure_E.coli_OR_poultry$Num_lb,
ci.ub=Occupational_Exposure_E.coli_OR_poultry$Num_ub, psize=1, xlab="",

    xlim=c(-5,26), slab=Occupational_Exposure_E.coli_OR_poultry$Author, cex=0.95, ylim=c(1,17.5),
rows=c(1:14), refline = 1, alim=c(0, 20))

text(23,14.9, "Occupational exposure to poultry E. coli OR", pos=2,cex=1,font=2)


#View(Occupational_Exposure_E.coli_OR_poultry)


# Veterinarian staff

forest(Occupational_Exposure_SAureus_OR_vets$Num_Estimate,
ci.lb=Occupational_Exposure_SAureus_OR_vets$Num_lb,
ci.ub=Occupational_Exposure_SAureus_OR_vets$Num_ub, psize=1, xlab="",

    xlim=c(-5,26), slab=Occupational_Exposure_SAureus_OR_vets$Author, cex=0.95, ylim=c(1,6),
rows=c(1:4), refline = 1, alim=c(0, 20))
```

text(22,3.5, "Occupational exposure veterinarian staff S. aureus OR", pos=2,cex=1,font=2)

Pet to human E. Coli

forest(Pet_to_human_ecoli_OR$Num_Estimate, ci.lb=Pet_to_human_ecoli_OR$Num_lb, ci.ub=Pet_to_human_ecoli_OR$Num_ub, psize=1, xlab="",

xlim=c(-5,26), slab=Pet_to_human_ecoli_OR$Author, cex=0.95, ylim=c(1,5), rows=c(1:2), refline = 1, alim=c(0, 20))

text(11,2.5, "Pet to human E. coli OR", pos=2,cex=1,font=2)

forest(Pet_to_human_staph_OR$Num_Estimate, ci.lb=Pet_to_human_staph_OR$Num_lb, ci.ub=Pet_to_human_staph_OR$Num_ub, psize=1, xlab="",

xlim=c(-10,26), slab=Pet_to_human_staph_OR$Author, cex=0.95, ylim=c(1,5), rows=c(1:2), refline = 1, alim=c(0, 20))

text(16,2.5, "Pet to human Staphylococci OR", pos=2,cex=1,font=2)

View(Pet_to_human_staph_OR)

Sharing a room

Sharing_room_entero_OR$Author<-as.character(Sharing_room_entero_OR$Author)

Sharing_room_entero_OR$Author[2]<-"Schwartz-Neiderman"

forest(Sharing_room_entero_OR$Num_Estimate, ci.lb=Sharing_room_entero_OR$Num_lb, ci.ub=Sharing_room_entero_OR$Num_ub, psize=1, xlab="",

xlim=c(-8,26), slab=Sharing_room_entero_OR$Author, cex=0.95, ylim=c(1,5.5), rows=c(1:3), refline = 1, alim=c(0, 20))

text(18,3.3, "Sharing a room Enterobacteriaceae OR", pos=2,cex=1,font=2)

Travel

South ASia

S aureus

forest(Travelling_OR_saures_South_asia$Num_Estimate, ci.lb=Travelling_OR_saures_South_asia$Num_lb, ci.ub=Travelling_OR_saures_South_asia$Num_ub, psize=1, xlab="",

```
      xlim=c(-8,26), slab=Travelling_OR_saures_South_asia$Author, cex=0.95, ylim=c(1,4.5), rows=c(1:2), refline = 1, alim=c(0, 20))

text(18,2.3, "Travelling to South Asia S. aureus OR", pos=2,cex=1,font=2)


  # E.coli

forest(Travelling_OR_Ecoli_SA$Num_Estimate, ci.lb=Travelling_OR_Ecoli_SA$Num_lb, ci.ub=Travelling_OR_Ecoli_SA$Num_ub, psize=1, xlab="",

      xlim=c(-8,26), slab=Travelling_OR_Ecoli_SA$Author, cex=0.95, ylim=c(1,6), rows=c(1:3), refline = 1, alim=c(0, 20))

text(23,3.5, "Travelling to South Asia E. coli OR", pos=2,cex=1,font=2)


# Enterobacteriacea

forest(Travelling_OR_Entero_SA$Num_Estimate, ci.lb=Travelling_OR_Entero_SA$Num_lb, ci.ub=Travelling_OR_Entero_SA$Num_ub, psize=1, xlab="",

      xlim=c(-8,26), slab=Travelling_OR_Entero_SA$Author, cex=0.95, ylim=c(1,6), rows=c(1:3), refline = 1, alim=c(0, 20))

text(23,3.5, "Travelling to South Asia Enterobacteriacea OR", pos=2,cex=1,font=2)


# South East Asia

  # Enterobacteriaceae

forest(Travelling_OR_Entero_SEA$Num_Estimate, ci.lb=Travelling_OR_Entero_SEA$Num_lb, ci.ub=Travelling_OR_Entero_SEA$Num_ub, psize=1, xlab="",

      xlim=c(-10,40), slab=Travelling_OR_Entero_SEA$Author, cex=0.95, ylim=c(1,8), rows=c(1:4), refline = 1, alim=c(0, 20))

text(25,5, "Travelling to South East Asia Enterobacteriaceae OR", pos=2,cex=1,font=2)


# Western Asia

  # Enterobacteriaceae

forest(Travelling_OR_Entero_WA$Num_Estimate, ci.lb=Travelling_OR_Entero_WA$Num_lb, ci.ub=Travelling_OR_Entero_WA$Num_ub, psize=1, xlab="",

      xlim=c(-8,26), slab=Travelling_OR_Entero_WA$Author, cex=0.95, ylim=c(1,6), rows=c(1:3), refline = 1, alim=c(0, 20))

text(18,3.5, "Travelling to Western Asia Enterobacteriaceae", pos=2,cex=1,font=2)
```

Asia (unspecified)

Enterobacteriaceae

forest(Travelling_OR_Entero_Asia_unsp$Num_Estimate, ci.lb=Travelling_OR_Entero_Asia_unsp$Num_lb, ci.ub=Travelling_OR_Entero_Asia_unsp$Num_ub, psize=1, xlab="",

xlim=c(-8,26), slab=Travelling_OR_Entero_Asia_unsp$Author, cex=0.95, ylim=c(1,6), rows=c(1:3), refline = 1, alim=c(0, 20))

text(18,3.5, "Travelling to Asia Enterobacteriaceae OR", pos=2,cex=1,font=2)

Latin America

Enterobacteriaceae

forest(Travelling_OR_Entero_LatinAmerica$Num_Estimate, ci.lb=Travelling_OR_Entero_LatinAmerica$Num_lb, ci.ub=Travelling_OR_Entero_LatinAmerica$Num_ub, psize=1, xlab="",

xlim=c(-8,26), slab=Travelling_OR_Entero_LatinAmerica$Author, cex=0.95, ylim=c(1,4.5), rows=c(1:2), refline = 1, alim=c(0, 20))

text(18,2.3, "Travelling to Laten America Enterobacteriaceae", pos=2,cex=1,font=2)

America

Enterobacteriaceae

forest(Travelling_OR_Entero_America$Num_Estimate, ci.lb=Travelling_OR_Entero_America$Num_lb, ci.ub=Travelling_OR_Entero_America$Num_ub, psize=1, xlab="",

xlim=c(-8,26), slab=Travelling_OR_Entero_America$Author, cex=0.95, ylim=c(1,4.5), rows=c(1:2), refline = 1, alim=c(0, 20))

text(18,2.3, "Travelling to America Enterobacteriaceae", pos=2,cex=1,font=2)

Africa

Enterobacteriaceae

forest(Travelling_OR_Entero_Africa$Num_Estimate, ci.lb=Travelling_OR_Entero_Africa$Num_lb, ci.ub=Travelling_OR_Entero_Africa$Num_ub, psize=1, xlab="",

xlim=c(-8,26), slab=Travelling_OR_Entero_Africa$Author, cex=0.95, ylim=c(1,8), rows=c(1:4), refline = 1, alim=c(0, 20))

text(18,5, "Travelling to Africa Enterobacteriaceae OR", pos=2,cex=1,font=2)

Europe

Enterobacteriaceae

forest(Travelling_OR_Entero_Europe$Num_Estimate, ci.lb=Travelling_OR_Entero_Europe$Num_lb, ci.ub=Travelling_OR_Entero_Europe$Num_ub, psize=1, xlab="",

xlim=c(-8,26), slab=Travelling_OR_Entero_Europe$Author, cex=0.95, ylim=c(1,6), rows=c(1:3), refline = 1, alim=c(0, 20))

text(18,3.5, "Travelling to Europe Enterobacteriaceae OR", pos=2,cex=1,font=2)

Prevalence Ratio

#Eating red meat

red_pr$Author<-as.character(red_pr$Author)

red_pr$Author[3:4]<-"Samore"

red_pr$Author[1]<-"Samore"

forest(red_pr$Num_Estimate, ci.lb=red_pr$Num_lb, ci.ub=red_pr$Num_ub, psize=1, xlab="",

xlim=c(-5,26), slab=red_pr$Author, cex=0.95, ylim=c(0.8,7), rows=c(1:4), refline = 1, alim=c(0, 20))

text(15,4.5, "Eating red meat E. coli PR", pos=2,cex=1,font=2)

Fam mem colonised

forest(Family_member_colonised_entero_PR$Num_Estimate, ci.lb=Family_member_colonised_entero_PR$Num_lb, ci.ub=Family_member_colonised_entero_PR$Num_ub, psize=1, xlab="",

xlim=c(-5,26), slab=Family_member_colonised_entero_PR$Author, cex=0.95, ylim=c(1,5), rows=c(1:2), refline = 1, alim=c(0, 20))

text(17,2.5, "Family member colonised Enterobacteriaceae PR", pos=2,cex=1,font=2)

Occupational Exposure pig

Occupational_Exposure_SAureus_PR_Pig$Author<-as.character(Occupational_Exposure_SAureus_PR_Pig$Author)

Occupational_Exposure_SAureus_PR_Pig$Author[4:9]<-"Rinsky"

Occupational_Exposure_SAureus_PR_Pig$Author[1:3]<-"Wardyn"

forest(Occupational_Exposure_SAureus_PR_Pig$Num_Estimate, ci.lb=Occupational_Exposure_SAureus_PR_Pig$Num_lb, ci.ub=Occupational_Exposure_SAureus_PR_Pig$Num_ub, psize=1, xlab="",

```
	xlim=c(-5,26), slab=Occupational_Exposure_SAureus_PR_Pig$Author, cex=0.95, ylim=c(1,20), rows=c(1:16), refline = 1, alim=c(0, 20))

text(20,17, "Family member occupational exposure to pig S. aureus PR", pos=2,cex=1,font=2)


forest(Occupational_Exposure_Staph_pig_PR$Num_Estimate, ci.lb=Occupational_Exposure_Staph_pig_PR$Num_lb, ci.ub=Occupational_Exposure_Staph_pig_PR$Num_ub, psize=1, xlab="",

	xlim=c(-4,26), slab=Occupational_Exposure_Staph_pig_PR$Author, cex=0.95, ylim=c(0,8), rows=c(1:5), refline = 1, alim=c(0, 20))

text(20,5.5, "Family member occupational exposure to pig Staphylococci PR", pos=2,cex=1,font=2)


Occupational_Exposure_SAureus_PR_poultry$Author<-as.character(Occupational_Exposure_SAureus_PR_poultry$Author)

Occupational_Exposure_SAureus_PR_poultry$Author[1:2]<-"Rinsky"

Occupational_Exposure_SAureus_PR_poultry$Author[3]<-"Wardyn"

forest(Occupational_Exposure_SAureus_PR_poultry$Num_Estimate, ci.lb=Occupational_Exposure_SAureus_PR_poultry$Num_lb, ci.ub=Occupational_Exposure_SAureus_PR_poultry$Num_ub, psize=1, xlab="",

	xlim=c(-4,26), slab=Occupational_Exposure_SAureus_PR_poultry$Author, cex=0.95, ylim=c(0,6), rows=c(1:3), refline = 1, alim=c(0, 20))

text(20,3.5, "Family member occupational exposure to poultry S. aureus PR", pos=2,cex=1,font=2)


#R0


#Animal to Animal

forest(animal_to_animal_Saurues_r0$Num_Estimate, ci.lb=animal_to_animal_Saurues_r0$Num_lb, ci.ub=animal_to_animal_Saurues_r0$Num_ub, psize=1, xlab="",

	xlim=c(-4,26), slab=animal_to_animal_Saurues_r0$Author, cex=0.95, ylim=c(1,5), rows=c(1:2), refline = 0, alim=c(0, 20))

text(15,2.5, "Animal to animal S. aureus R0", pos=2,cex=1,font=2)


# Contact with infected person

Contact_with_infected_Saurues_r0$Author<-as.character(Contact_with_infected_Saurues_r0$Author)
```

Contact_with_infected_Saurues_r0$Author[1]<-"Christopher"

forest(Contact_with_infected_Saurues_r0$Num_Estimate, ci.lb=Contact_with_infected_Saurues_r0$Num_lb, ci.ub=Contact_with_infected_Saurues_r0$Num_ub, psize=1, xlab="",

xlim=c(-8,26), slab=Contact_with_infected_Saurues_r0$Author, cex=0.95, ylim=c(1,6), rows=c(1:11), refline = 0, alim=c(0, 20))

text(17,3.5, "Contact with infected person S. aureus R0", pos=2,cex=1,font=2)

Risk ratio

forest(Contact_with_infected_Saurues_RR$Num_Estimate, ci.lb=Contact_with_infected_Saurues_RR$Num_lb, ci.ub=Contact_with_infected_Saurues_RR$Num_ub, psize=1, xlab="Risk Ratio",

xlim=c(-6,26), slab=Contact_with_infected_Saurues_RR$Author, cex=0.95, ylim=c(1,5), rows=c(1:2), refline = 0, alim=c(0, 20))

text(15,2.5, "Contact with infected person S. aureus RR", pos=2,cex=1,font=2)

#Risks

Animal to air kan niet want risk heeft geen CIs

forest(pig_to_air_Ecoli_Risk$Num_Estimate, ci.lb=pig_to_air_Ecoli_Risk$Num_lb, ci.ub=pig_to_air_Ecoli_Risk$Num_ub, psize=1, xlab="",

xlim=c(-3,1), slab=pig_to_air_Ecoli_Risk$Author, cex=0.95, ylim=c(0,5), rows=c(1:2), refline = 0, alim=c(0, 2))

forest(Cattle_to_air_Sareus_Risk$Num_Estimate, ci.lb=Cattle_to_air_Sareus_Risk$Num_lb, ci.ub=Cattle_to_air_Sareus_Risk$Num_ub, psize=1, xlab="",

xlim=c(-3,1), slab=Cattle_to_air_Sareus_Risk$Author, cex=0.95, ylim=c(0,5), rows=c(1:22), refline = 0, alim=c(0, 2))
